# Self‐Accelerating Bimetallic Peroxide Nanozymes for Cascade‐Amplified Pyroptosis‐Immunotherapy

**DOI:** 10.1002/advs.75441

**Published:** 2026-04-23

**Authors:** Xuanyi Lu, Liang Li, Siyu Pan, Yuehan Jian, Yuhan Chen, Wenjie Yang, Guang Song, Xueyang Fang, Ping'an Ma, Lijun Jiang

**Affiliations:** ^1^ Hubei Key Laboratory of Genetic Regulation and Integrative Biology School of Life Sciences Key Laboratory of Pesticide & Chemical Biology of Ministry of Education Central China Normal University Wuhan China; ^2^ MOE Key Laboratory of Laser Life Science & Institute of Laser Life Science Guangdong Provincial Key Laboratory of Laser Life Science College of Biophotonics School of Optoelectronic Science and Engineering South China Normal University Guangzhou China; ^3^ Key Laboratory of Superlight Materials and Surface Technology College of Materials Science and Chemical Engineering Ministry of Education Harbin Engineering University Harbin China

**Keywords:** bimetallic peroxides, catalytic therapy, pyroptosis, reactive oxygen species, tumor immunotherapy

## Abstract

Despite the promise of hydrogen peroxide (H_2_O_2_)‐mediated cancer therapy, its efficacy is often constrained by the insufficient endogenous H_2_O_2_ levels and immunosuppressive tumor microenvironment (TME). To address this, we designed a bimetallic peroxide nanosystem (CuZnONPs) that executes a triple‐combination therapeutic strategy. In the weakly acidic TME, CuZnONPs self‐supply H_2_O_2_, exert enzyme‐mimetic activities to catalyze H_2_O_2_ into toxic reactive oxygen species (·OH and ·O_2_^−^) and O_2_, and release Zn^2+^ to activate pyroptosis. Density functional theory calculations reveal that the single Cu atoms in CuZnONPs play a critical role by not only conferring peroxidase‐like activity for ·OH generation but also modulating the electronic structure of adjacent Zn sites to drive cascade catalase‐ and oxidase‐like activities for ·O_2_^−^ production. The resulting reactive oxygen species burst downregulates the GSH/GPX4 axis, disrupts redox homeostasis, and inflicts extensive damage to lipids, mitochondria, and DNA. Furthermore, Zn^2+^‐activated pyroptosis elicits damage‐associated molecular pattern release to promote dendritic cells maturation and remodel the inflammatory tumor microenvironment, ultimately converting cold tumors into hot tumors. This work establishes a TME‐responsive nanoplatform that synergistically integrates catalytic therapy with pyroptosis‐enhanced immunotherapy, offering new insights into the design of nanomedicines for cancer therapy.

## Graphical abstract

Acidic tumor microenvironment‐responsive CuZnONPs enable triple‐combination therapy via self‐supplying H_2_O_2_, cascade nanozyme activities for ROS burst, and Zn^2+^‐activated pyroptosis, synergizing catalytic therapy with immunotherapy to convert cold tumors into hot ones.

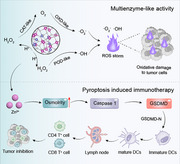

## 1 Introduction

In recent years, nanocatalysts have gained significant attention due to their broad applicability, spanning from energy transformation to the biological field [1, 2]. Particularly in cancer therapy, they enable the in situ generation of therapeutic species by exploiting specific tumor microenvironment (TME) characteristics, such as mild acidity, elevated GSH levels, or accumulated hydrogen peroxide (H_2_O_2_). Such localized catalysis enables selective tumor cell elimination through the production of reactive oxygen species (ROS), nitric oxide, or carbon monoxide [3]. Among various catalytic platforms, nanozymes capable of producing cytotoxic ROS have attracted considerable attention [4, 5], with H_2_O_2_ serving as one of the primary substrates. For instance, we previously reported a dual single‐atom catalyst, Fe/Mn@PSe_3_, which can convert H_2_O_2_ into ·OH for chemodynamic therapy [6]. Although tumor cells exhibit higher basal H_2_O_2_ concentrations (50–100 µM) [7] than normal cells (1–700 nM) [8], the endogenous levels remain inadequate to sustain robust catalytic reactions. Therefore, developing strategies to specifically elevate intratumoral H_2_O_2_ levels is critical for achieving optimal therapeutic outcomes.

Metal peroxides, characterized by labile peroxide bonds, have been extensively investigated as exogenous H_2_O_2_ donors due to their spontaneous decomposition under weakly acidic TME conditions. Common examples include copper peroxide (CuO_2_), zinc peroxide (ZnO_2_), and calcium peroxide (CaO_2_) [9]. Beyond self‐suppling H_2_O_2_, the incorporation of secondary metal ions can introduce additional catalytic and immunomodulatory functionalities [10, 11]. Bimetallic peroxides combine the advantages of distinct metal species to enhance antitumor efficacy. For instance, a recent study on Ca/Cu bimetallic peroxides demonstrated synergistic effects through Cu^2+^‐mediated Fenton‐like reactions and Ca^2+^‐induced mitochondrial dysfunction [12]. Metal ions such as Zn^2+^ and Mn^2+^ can act as immunoadjuvants by activating immune signaling pathways such as cGAS‐STING and promoting T cell‐mediated antitumor immunity [13, 14]. Notably, Zn^2+^ has recently been shown to trigger pyroptosis, a pro‐inflammatory programmed cell death characterized by gasdermin protein cleavage and the release of damage‐associated molecular patterns (DAMPs) [15]. These DAMPs promote dendritic cells (DCs) maturation and subsequent cytotoxic T cell activation, thereby inducing a robust immune response [16, 17, 18]. Consequently, Zn^2+^‐based nanomaterials represent a promising platform for pyroptosis‐mediated immunotherapy [15]. On this basis, we propose that Cu/Zn bimetallic peroxides could synergistically enhance ROS generation and immune activation.

**SCHEME 1:**
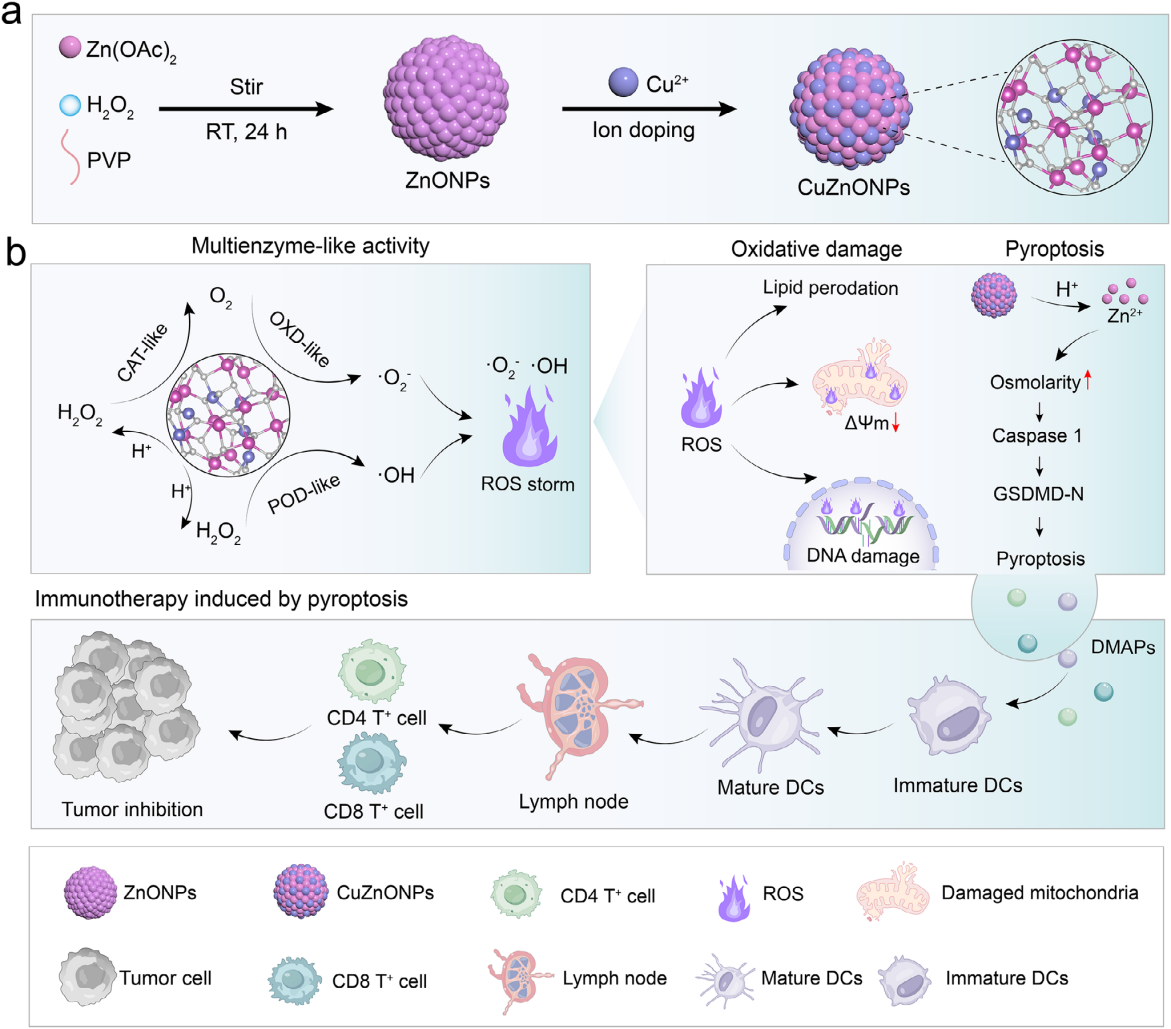
(a) Schematic illustration showing the fabrication of CuZnONPs. (b) The mechanism of CuZnONPs for cancer catalytic therapy and pyroptosis‐induced immunotherapy.

In this work, we designed a bimetallic peroxide nanozyme (CuZnONPs) that combines catalytic therapy with metal ion‐mediated immunotherapy (Scheme 1). By doping Cu single atoms into a ZnO_2_ matrix, the resulting CuZnONPs respond to acidic TME conditions to supply H_2_O_2_ and release Zn^2+^ ions. The nanozyme also exhibits catalase (CAT)‐, peroxidase (POD)‐, and oxidase (OXD)‐like activities, enabling further conversion of H_2_O_2_ into O_2_, ·OH, and ·O_2_^−^ to alleviate hypoxia and induce oxidative damage. Density functional theory (DFT) calculations elucidated the electronic structures and catalytic mechanisms underlying these multi‐enzyme activities, revealing that single Cu atoms not only confer POD‐like activity but also modulate the electronic structure of adjacent Zn sites to drive cascade CAT‐ and OXD‐like activities. Furthermore, the released Zn^2+^ ions synergize with ROS to activate the Caspase‐1/GSDMD‐mediated pyroptosis pathway, promoting DAMPs release and DCs‐mediated T cell activation. Together, this system constitutes an effective strategy for synergistic nanocatalytic therapy and pyroptosis‐enhanced immunotherapy, offering a new multimodal paradigm for cancer treatment.

## 2 Results and Discussion

### 2.1 Preparation and Characterization of CuZnONPs

**FIGURE 1:**
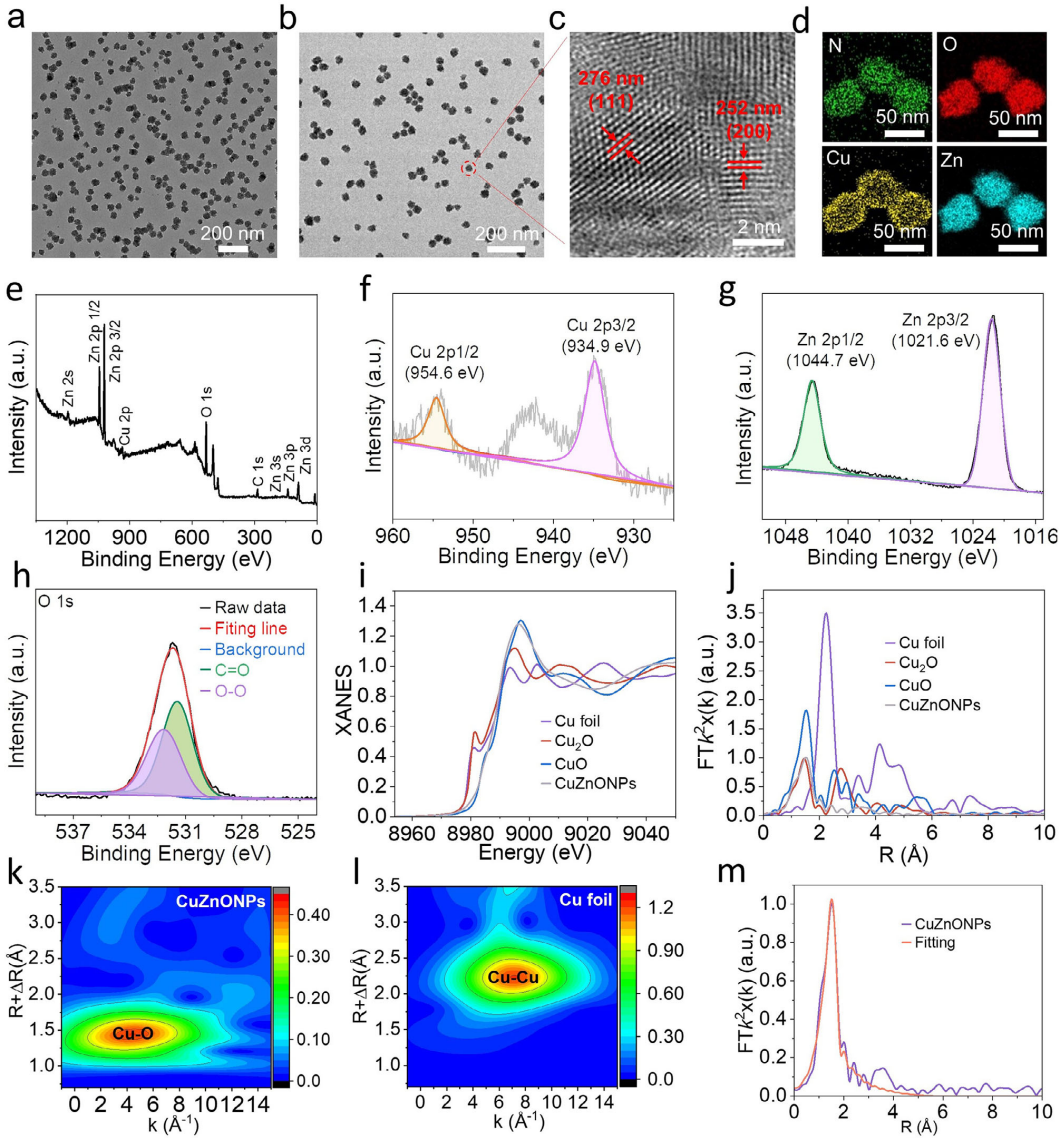
(a) TEM image of ZnONPs. (b) TEM image, (c) high‐resolution TEM image, and (d) elemental mapping of CuZnONPs. (e) XPS spectrum of CuZnONPs. High‐resolution XPS spectra of (f) Cu 2p, (g) Zn 2p, and (h) O 1s of CuZnONPs. (i) XANES spectra at the Cu K‐edge and (j) EXAFS spectra in R space at the Cu K‐edge of Cu foil, Cu_2_O, CuO and CuZnONPs. (k) EXAFS fitting curve of CuZnONPs at R space. Wavelet transform of Cu K‐edge EXAFS of (l) Cu foil and (m) CuZnONPs.

The fabrication procedure of CuZnONPs is illustrated in Scheme 1a. ZnONPs were first synthesized via chemical precipitation using poly(vinylpyrrolidone) (PVP) as a stabilizer. Figure 1a and Figure S1a show a diameter of 49.3 ± 3.9 nm of ZnONPs, as determined by the transmission electron microscopy (TEM) images. Following a Cu‐doping method, CuZnONPs were carefully synthesized based on a controlled cation exchange process. The Cu doping caused an apparent color change from milky white to yellow‐green by the naked eye (Figure S2).

TEM image showed that CuZnONPs exhibited a uniform particle size distribution with an average diameter of 44.4 ± 4.2 nm and showed no apparent aggregation (Figure 1b and Figure S1b). High‐resolution TEM (HRTEM) images further showed clear lattice fringes with spacings of approximately 0.252 and 0.276 nm, corresponding to the (111) and (200) planes, respectively (Figure 1c). Dynamic light scattering (DLS) measurements indicated that CuZnONPs were well dispersed in deionized water, exhibiting a hydrodynamic diameter of 65.5 ± 2.7 nm (Figure S3). Elemental mapping via energy dispersive X‐ray spectroscopy (EDS) confirmed the presence of N, O, Cu, and Zn in CuZnONPs, with Cu predominantly located at the edge of the nanoparticles (Figure 1d). Fourier transform infrared (FTIR) spectroscopy showed a distinctive peak at 1634 cm^−1^, attributed to the C = O stretching vibration of PVP, confirming the successful synthesis of PVP‐stabilized CuZnONPs (Figure S4) [19, 20]. XRD analysis showed that the diffraction peaks of CuZnONPs were almost identical to those of ZnONPs and were consistent with the crystal characteristics of ZnO_2_ reported in the literature (Figure S5) [21, 22]. To assess stability under physiologically relevant conditions, CuZnONPs were incubated in different media for 0, 7, and 30 days. In water and PBS, the hydrodynamic diameter and zeta potential remained stable without significant changes. In medium containing 10% fetal bovine serum, a slight increase in particle size was observed; however, the nanoparticles remained well dispersed with no notable aggregation or precipitation (see photographs in Table S1). These results demonstrate that CuZnONPs possess excellent long‐term stability under physiologically relevant conditions.

We next examined the chemical composition and electronic state of elements in CuZnONPs. X‐ray photoelectron spectroscopy (XPS) spectrum of CuZnONPs showed characteristic peaks of Cu 2p, Zn 2p, O 1s, and C 1s (Figure 1e). To define the oxidation state at the atomic level, we conducted high‐resolution XPS. The Cu 2p spectrum displayed two typical peaks at 953.0 eV and 935.2 eV, attributed to the Cu^2+^ oxidation state (Figure 1f) [23]. The Zn 2p exhibited two distinct peaks at 1044.7 eV and 1021.4 eV, and the O 1s showed a characteristic peak at 532.2 eV (Figure 1g,h), indicating that Zn and O exist as Zn^2+^ and O^−^ species in the CuZnONPs, respectively [24]. To further verify the oxidation state of the doped Cu atoms, X‐ray absorption near‐edge structure (XANES) measurements of CuZnONPs were performed at the Cu K‐edge, using Cu foil, Cu_2_O, and CuO as references [25]. Comparative analysis of the spectral features between CuZnONPs and the reference samples suggested that Cu predominantly exists in the +2 oxidation state, in agreement with the XPS data (Figure 1i). We also examined the local coordination environment of Cu atoms in CuZnONPs using extended X‐ray absorption fine structure (EXAFS). EXAFS spectrum of CuZnONPs exhibited one main peak at 1.50 Å (Figure 1j), corresponding to Cu─O scattering. Compared to the spectrum of Cu foil, the Cu─Cu scattering path at around 2.23 Å was absent in CuZnONPs, suggesting the dispersion of single Cu atoms in CuZnONPs. To more clearly reveal the coordination environment, the wavelet transform (WT) analysis of the EXAFS spectra was performed. The WT contour plot of CuZnONPs presented only one intensity maxima at *R* = 1.5 Å (Figure 1k), which was attributed to the Cu─O bond. While in the plot of Cu foil, Cu_2_O, and CuO, the maximum intensity value assigned to Cu─Cu bond was clearly demonstrated (Figure 1l and Figure S6). To further obtain quantitative information on the Cu coordination from the EXAFS data, the nonlinear least‐squares curve fitting was performed (Figure 1m and Figure S7). The fitting results indicated that each Cu atom is coordinated to 3.4 ± 0.4 O atoms with a bond length of 1.95 ± 0.01 Å (Table S2). Together, these results confirm that Cu is present as single atoms coordinated exclusively with O in CuZnONPs.

### 2.2 Enzyme‐Mimicking Catalytic Activities of CuZnONPs

**FIGURE 2:**
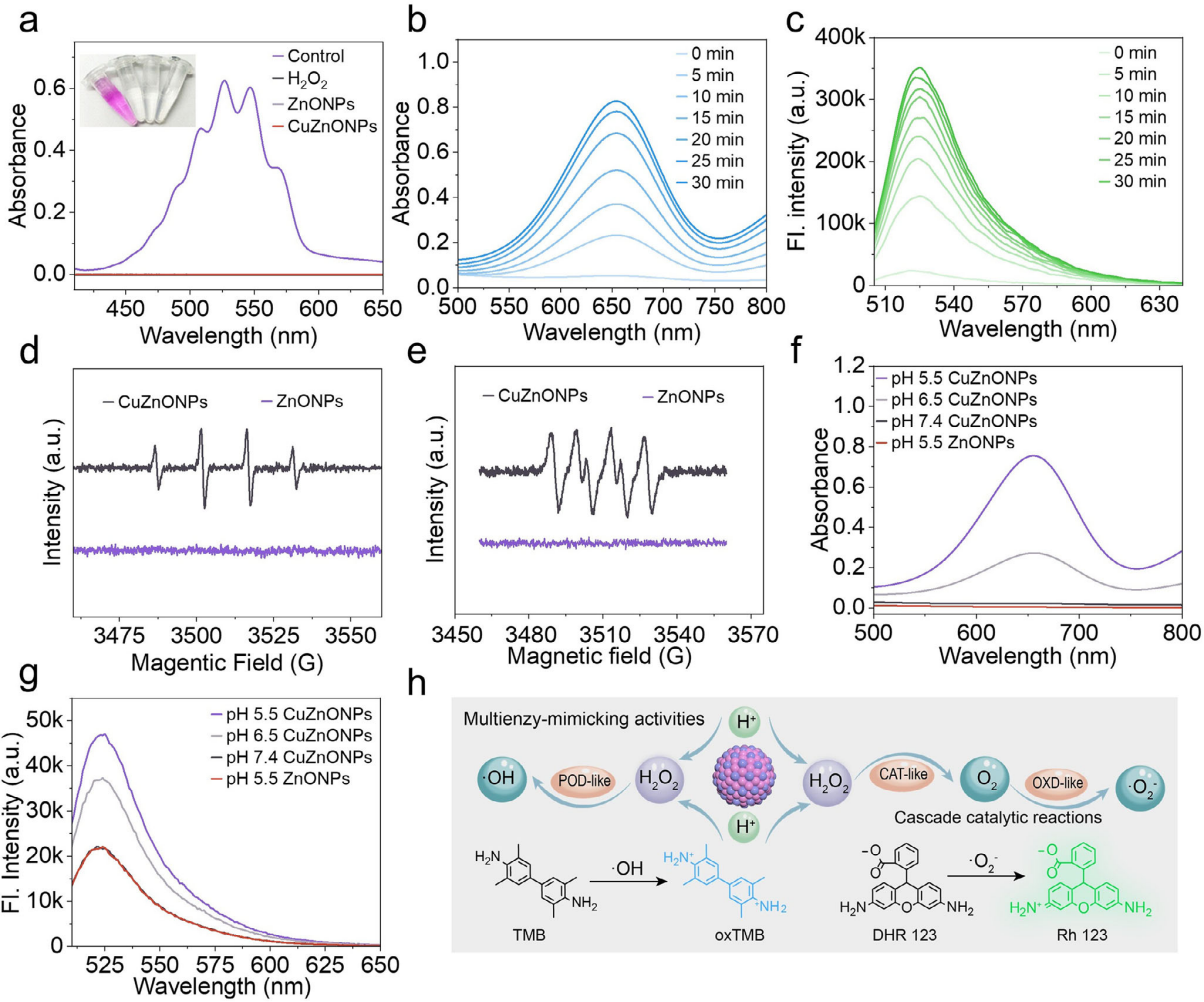
(a) UV–Vis absorption spectra and photographs (inset) of KMnO_4_ solutions treated with control, H_2_O_2_, ZnONPs, and CuZnONPs. Time‐dependent generation of (b) ·OH and (c) ·O_2_^−^ by CuZnONPs, as monitored by the spectral changes of TMB and DHR 123, respectively. Generation of (d) ·OH and (e) ·O_2_^−^ by CuZnONPs, as detected by ESR. Generation of (f)·OH and (g) ·O_2_^−^ in 7.4, 6.5, and 5.5 PBS buffers. (h) Schematic illustration showing the multienzy‐mimicking activities of CuZnONPs.

We first investigated whether the bimetallic peroxide CuZnONPs could self‐decompose under acidic conditions to release H_2_O_2_. As shown in Figure 2a, the presence of CuZnONPs led to the disappearance of both the KMnO_4_ absorption and its purple color, a phenomenon also observed in the presence of H_2_O_2_ or ZnONPs. This is attributed to the reduction of MnO_4_^−^ to Mn^2+^ by the released H_2_O_2_ [26].

Given the presence of atomically dispersed Cu atoms, we further assessed the enzyme‐mimicking activities of CuZnONPs in catalyzing H_2_O_2_. Specifically, we examined three enzyme‐like activities, including CAT‐, POD‐, and OXD‐like behaviours. The CAT‐like activity was evaluated by monitoring dissolved O_2_ generation. As illustrated in Figure S8a, addition of H_2_O_2_ to CuZnONPs triggered a time‐dependent increase in O_2_ level, with significantly higher production at pH 5.5 than at pH 6.5 or 7.4. Michaelis‐Menten analysis yielded *K*_m_ of 13.45 mM and *V*_max_ of 0.58 µM s^−1^ (Figure S8b,c), confirming efficient CAT‐like catalytic performance.

We next evaluated POD‐ and OXD‐like activities by tracking spectral changes of the substrates 3,3’,5,5’‐tetramethylbenzidine (TMB) [27, 28] and Dihydrorhodamine 123 (DHR 123) [29, 30], respectively. CuZnONPs induced time‐dependent increases in TMB absorption (Figure 2b) and DHR 123 fluorescence (Figure 2c). In contrast, no spectral changes for either TMB or DHR 123 were observed with ZnONPs under the same condition (Figures S9 and S10), underscoring the essential role of atomically dispersed Cu in generating ·OH and ·O_2_^−^. The ROS production was further verified to be concentration‐dependent (Figure S11) and corroborated by electron spin resonance (ESR) spectroscopy, where characteristic signals were detected exclusively for CuZnONPs (Figure 2d,e). The catalytic activities exhibited strong pH dependence, with both POD‐ and OXD‐like functions most active at pH 5.5 (Figure 2f,g). This pH‐responsive behavior underscores the TME‐adaptive nature of CuZnONPs. Steady‐state kinetic analyses were then conducted at pH 5.5 PBS buffer. The Michaelis‐Menten curves and Lineweaver‐Burk plots are shown in Figure S12. The *K*_m_ values were calculated to be 50.00 mM for POD and 1.00 mM for OXD, with corresponding *V*_m__a__x_ values of 0.25 µM s^−1^ and 0.48 µM s^−1^, respectively. Taken together, CuZnONPs exhibit multienzy‐mimicking activities that are markedly enhanced in acidic environments, highlighting their potential as a TME‐responsive catalytic platform (Figure 2h).

### 2.3 DFT Studies on the Enzymatic Activities of CuZnONPs

**FIGURE 3:**
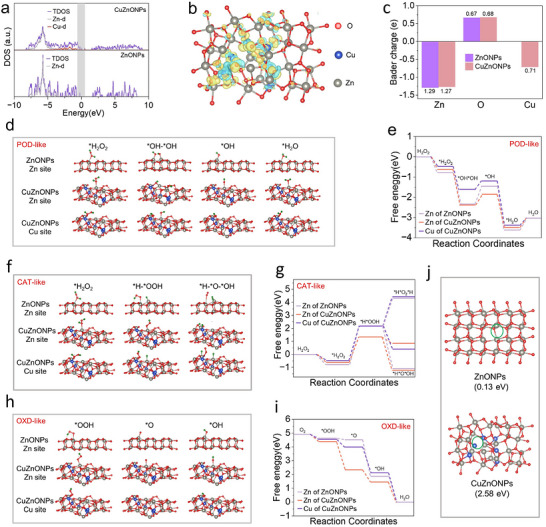
(a) Total DOS spectra of ZnONPs and CuZnONPs. (b) Differential charge density of CuZnONPs. (c) Bader charges of O, Cu, and Zn atoms in ZnONPs and CuZnONPs, respectively. (d) Surface structure models and (e) free energy diagrams of the POD‐like reaction on the three active sites of ZnONPs and CuZnONPs models. (f) Surface structure models and (g) free energy diagrams of the CAT‐like reaction on the three active sites of ZnONPs and CuZnONPs models. (h) Surface structure models and (i) free energy diagrams of the OXD‐like reaction on the three active sites of ZnONPs and CuZnONPs models. (j) Calculated oxygen vacancy formation enthalpy of ZnONPs and CuZnONPs structural models.

To elucidate the atomic structure and catalytic mechanism of CuZnONPs, DFT calculations were systematically performed. Initial structural optimization based on XRD and EXAFS data suggested that Cu preferentially occupies interstitial sites with a formation energy (Ef) of −2.57 eV, significantly lower than that of the substitutional doping model (−0.68 eV; Figure S13). Projected density of states (PDOS) analysis further demonstrated that Cu doping introduces new hybridized electronic states at the edges of the Fermi level in CuZnONPs (Figure 3a), suggesting that electrons can be more readily excited into the conduction band [31], in contrast to the sharp density of states (DOS) in ZnONPs that suggests limited catalytic activity. Moreover, differential charge density and Bader charge analyses confirmed that Cu doping induces pronounced electron delocalization (Figure 3b,c), which strengthens the binding affinity of CuZnONPs towards reaction intermediates [32].

To further unravel the multi‐enzyme‐like catalytic mechanism at the atomic scale, three possible surface models were constructed: ZnONPs (200) with Zn sites as active centers, and CuZnONPs (200) with Zn or Cu sites as active centers. The configuration of the key intermediates involved in the POD‐like activity is illustrated in Figure 3d [4]. Reaction energy profiling revealed that the first two steps towards *H_2_O_2_ and *OH‐*OH proceed without an energy barrier across all models (Figure 3e). However, in the rate‐determining step of *OH‐*OH dissociation into *OH‐·OH, the Cu sites in CuZnONPs exhibited the lowest energy barrier (0.41 eV), highlighting the essential role of Cu sites in facilitating POD‐like activity.

We further simulated the catalytic pathways for CAT‐ and OXD‐like activities (Figure 3f,h). In CAT‐like activities, Zn sites in CuZnONPs showed the lowest energy barrier towards H*OOH and a negative free energy for *H*O_2_*H formation (Figure 3g). For OXD‐like activity, which follows a four‐electron oxygen reduction mechanism analogous to natural oxidases, in which O_2_ is first adsorbed onto metal sites and sequentially reduced to *OOH, *O, *OH, and *H_2_O (Figure 3i) [33]. The Zn sites in CuZnONPs exhibited the most negative free energy towards *OOH, reflecting superior O_2_ binding that weakens the O─O bond and induces greater O─O bond elongation. The Zn sites also showed the most favorable energetics for *O and *OH intermediates. These results indicate that Cu doping effectively modulates the electronic structure of Zn sites, significantly boosting their ability to catalyze H_2_O_2_ toward ·O_2_^−^ generation. Importantly, the oxygen vacancy formation energy of CuZnONPs was calculated to be 2.58 eV, markedly higher than that of ZnONPs (0.13 eV; Figure 3j), indicating a concurrent enhancement in structural stability upon Cu doping. Taken together, the Cu doping not only confers intrinsic POD‐like activity to CuZnONPs, enabling efficient ·OH generation from H_2_O_2_, but also modulates the electronic structure of adjacent Zn sites, driving cascade CAT‐ and OXD‐like activities for O_2_^−^ production.

### 2.4 In Vitro Cytotoxic Effect of CuZnONPs

**FIGURE 4:**
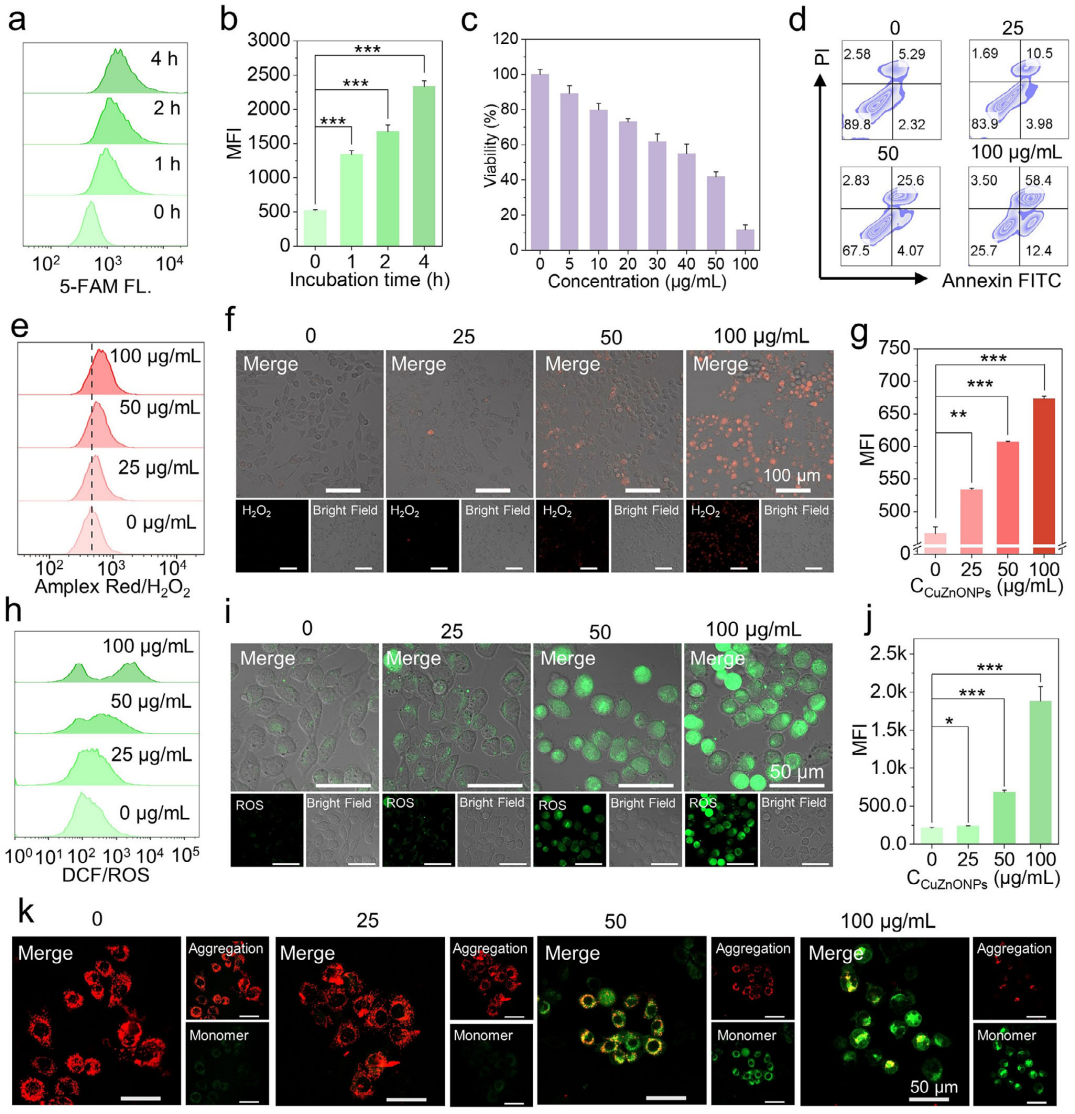
(a) Flow cytometric and (b) corresponding quantitative analysis of 4T1 cells treated with PBS or 5‐FAM‐CuZnONPs. (c) Cytotoxic effect of CuZnONPs on 4T1 cells. (d) Annexin V and PI co‐staining of 4T1 cells treated with CuZnONPs. (e) Amplex Red fluorescence and (g) the corresponding quantitative analysis of 4T1 cells treated with CuZnONPs, measured by flow cytometry. (f) CLSM images of 4T1 cells treated with varying concentrations of CuZnONPs, and stained with Amplex Red. (h) DCF fluorescence and (j) the corresponding quantitative analysis of 4T1 cells treated with CuZnONPs, measured by flow cytometry. (i) CLSM images of 4T1 cells treated with varying concentrations of CuZnONPs, and stained with DCFH‐DA. (k) CLSM images of 4T1 cells treated with varying concentrations of CuZnONPs, and stained with JC‐1. Data are presented as mean ± SD (*n* = 3), and statistical significance was assessed by a one‐way ANOVA. ^*^ *p* < 0.05, ^**^ *p* < 0.01, ^***^ *p* < 0.001.

Prior to investigating the cellular behaviour of CuZnONPs, we first examined their cellular uptake using 5‐Carboxyfluorescein (5‐FAM) as a labeling agent. As shown in Figure S14, 4T1 cells treated with 5‐FAM‐labeled CuZnONPs (termed as 5‐FAM‐CuZnONPs) displayed distinct green fluorescence compared to the control group, confirming successful internalization. Flow cytometry analysis further verified the time‐dependent cellular uptake of CuZnONPs, as evidenced by a gradual increase in the fluorescence intensity of 5‐FAM‐CuZnONPs in 4T1 cells with prolonged incubation time (Figure 4a,b).

The methyl thiazolyl tetrazolium (MTT) assay was then performed to assess the ROS effect of CuZnONPs on cell viability. As shown in Figure 4c, CuZnONPs exhibited a concentration‐dependent cytotoxic effect on 4T1 cells, with an inhibition rate approaching 90% at the concentration of 100 µg/mL. Furthermore, apoptosis analysis by Annexin V‐FITC/PI staining revealed that the rates of cell apoptosis were 14.9%, 33.8%, and 73.2% after treatment with 25, 50, and 100 µg/mL of CuZnONPs, respectively (Figure 4d and Figure S15). To visually confirm the cytotoxic effect, we performed calcein acetoxymethyl (Calcein AM) and propidium iodide (PI) staining. As observed in Figure S16, PI‐positive cells (red fluorescence, indicating dead cells) were readily detected at the concentration of 25 µg/mL, and the proportion of these cells increased with higher concentrations of CuZnONPs.

### 2.5 In Vitro ROS Production and Oxidative Damage Induced by CuZnONPs

Encouraged by the excellent cytotoxicity of CuZnONPs, we proceeded to evaluate their capacity to induce cellular ROS and the subsequent oxidative injury. We first assessed H_2_O_2_ generation using the Amplex Red/horseradish peroxidase (AR/HRP) assay [34]. A concentration‐dependent increase in red fluorescence was observed (Figure 4f). The observation was quantitatively corroborated by flow cytometry, which showed a progressive increase in fluorescence intensity as CuZnONPs concentrations increased from 0 to 100 µg/mL (Figure 4e,g). We next evaluated total cellular ROS levels using the fluorescent probe 2,7‐Dichlorodihydrofluorescein diacetate (DCFH‐DA) [35]. Treatment of 4T1 cells with 50 or 100 µg/mL CuZnONPs resulted in intense green fluorescence and notable morphological alterations (Figure 4i). Flow cytometry further confirmed a dose‐dependent escalation in total ROS (Figure 4h,j). To further evaluate the in vitro enzyme‐mimetic properties of CuZnONPs, we monitored cellular O_2_, ·OH, and ·O_2_^−^ dynamics using the hypoxia‐sensitive probe Ru(dpp)_3_Cl_2_ [36], hydroxyphenyl fluorescein (HPF) [37], and DHR 123, respectively. Confocal imaging of Ru(dpp)_3_Cl_2_ revealed a rapid fluorescence increase, peaking at 0.5 h after CuZnONPs treatment (Figure S17a), a pattern quantitatively supported by flow cytometry (Figure S17b,c). Concurrently, HPF and DHR 123 staining indicated a dose‐responsive increase in ·OH and ·O_2_^−^ levels (Figures S18 and S19). Together, these data substantiate the capacity of CuZnONPs to induce multiple forms of ROS in a cellular context.

We next asked whether the ROS burst triggered by CuZnONPs could disrupt the adaptive redox homeostasis in cancer cells. Since tumors often upregulate the GSH/GPX4 axis to maintain redox balance under high metabolic and oxidative stress [38], we targeted this pathway for investigation. Exposure to CuZnONPs led to an obvious decline in cellular GSH, as quantified using the GSH indicator 5, 5‐dithiobis‐2‐nitrobenzoic acid (DTNB), with levels dropping to about 60% of the control at 100 µg/mL of CuZnONPs (Figure S20). Concurrently, immunofluorescence staining and enzymatic assays revealed a concentration‐dependent downregulation of GPX4 expression and activity (Figures S21 and S22). These findings confirm that CuZnONPs impair key antioxidant defenses, leaving cells more vulnerable to oxidative damage.

Subsequently, we assessed the consequences of the redox imbalance triggered by CuZnONPs. Staining with the lipophilic dye DIO indicated progressive disruption of cell membrane integrity, as shown by the concentration‐dependent reduction in fluorescence (Figure S23). This was corroborated by a marked increase in malondialdehyde (MDA), a marker of lipid peroxidation, with levels rising to 1.3‐, 2.4‐, and 4.8‐fold of control levels when cells were treated with 25, 50, and 100 µg/mL of CuZnONPs, respectively (Figure S24). Mitochondrial integrity was also comprised, as evidenced by a decrease in red JC‐1 aggregates and a concomitant increase in green monomeric fluorescence, indicating depolarization of the mitochondrial membrane potential (Figure 4k) [39]. Finally, immunofluorescence staining of nuclear DNA showed intense green fluorescence in cells exposed to 50 and 100 µg/mL of CuZnONPs, signifying severe DNA damage (Figure S25). All these results demonstrated that CuZnONPs elicit robust ROS production, disrupt cellular redox homeostasis, and induce extensive damage to lipids, mitochondria, and DNA, collectively contributing to the observed cytotoxicity of CuZnONPs.

### 2.6 In Vitro Zn^2+^ Release and Pyroptosis Induced by CuZnONPs

The generation of H_2_O_2_ under acidic conditions is often accompanied by the gradual decomposition of metal peroxide, leading to the release of metal ions and potential intracellular metal overload [40]. To investigate this, we first quantified the release of Cu^2+^ and Zn^2+^ from CuZnONPs using inductively coupled plasma mass spectrometry (ICP‐MS). As shown in Figure S26, after 48 h of incubation at pH 5.5, 83.2% of Cu^2+^ and 54.6% of Zn^2+^ were released, significantly higher than the 16.7% and 3.0% observed at the neutral condition (pH 7.4), confirming an acidic‐triggered ion release profile.

**FIGURE 5:**
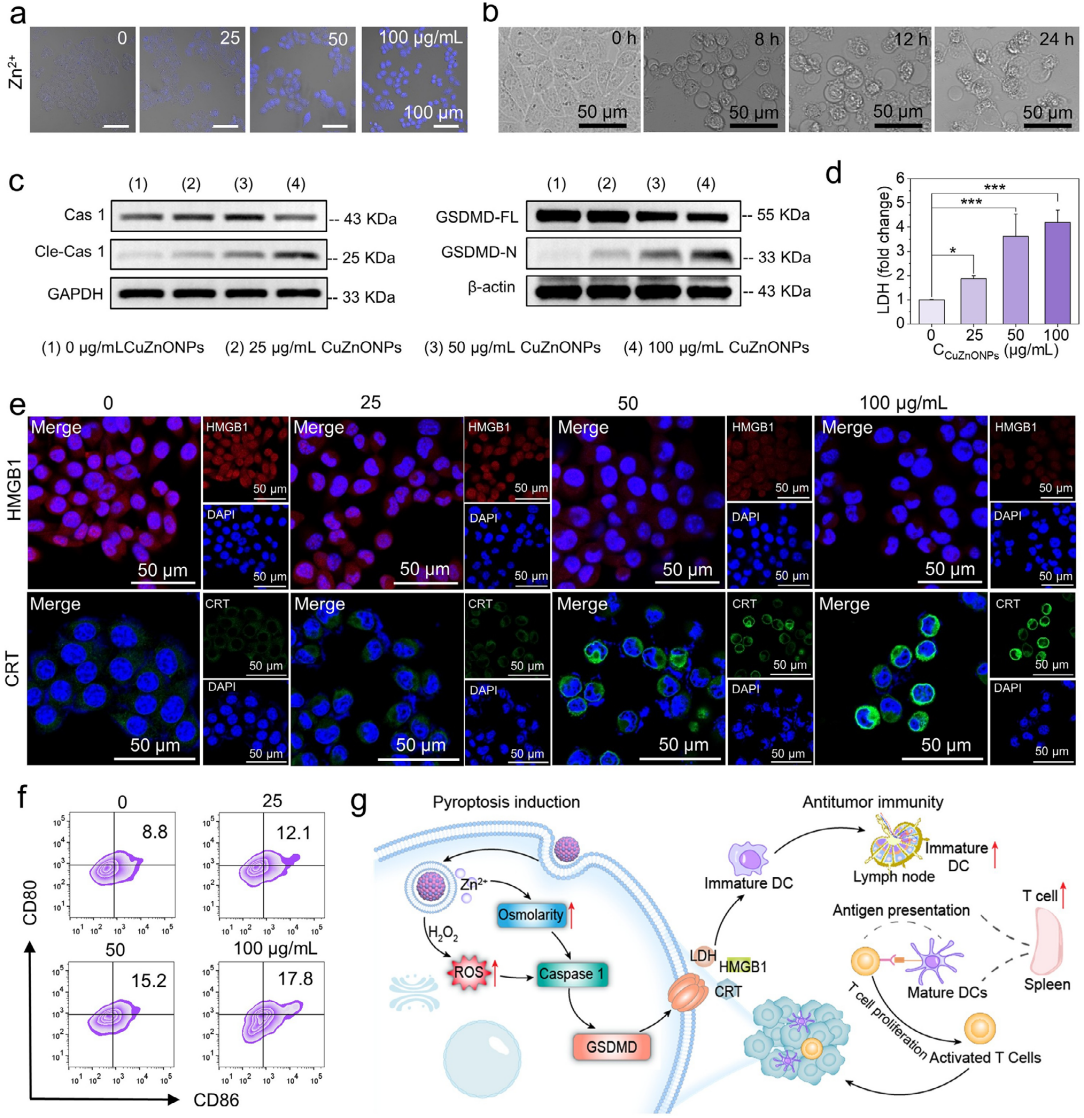
(a) CLSM images of 4T1 cells treated with CuZnONPs and stained with zinquin ethyl ester. (b) The cell morphology of 4T1 cells treated with CuZnONPs. (c) Western blot of Caspase 1, Cle‐Caspase 1, GSDMD, and GSDMD‐N in 4T1 cells treated with CuZnONPs. (d) The released LDH from 4T1 cells after treatment with CuZnONPs. (e) CLSM images of HMGB1 and CRT in 4T1 cells treated with CuZnONPs. (f) Flow cytometric analysis of BMDCs maturation (CD80^+^ CD86^+^ in CD11c^+^ cells) in supernatants of 4T1 cells incubating with CuZnONPs for 24 h. (g) Schematic illustration showing pyroptosis‐induced immunotherapy by CuZnONPs. Data are presented as mean ± SD (*n* = 3), and statistical significance was assessed by a one‐way ANOVA. ^*^ *p* < 0.05, ^**^ *p* < 0.01, and ^***^ *p* < 0.001.

Although Zn^2+^ is an essential nutrient, its elevated levels can induce pyroptosis. We therefore examined intracellular Zn^2+^ level upon CuZnONPs treatment using a Zn^2+^‐specific indicator, zinquin ethyl ester [41]. As shown in Figure 5a, CLSM images of treated 4T1 cells exhibited a concentration‐dependent increase in zinquin ethyl ester fluorescence, indicating a substantial increase in Zn^2+^ level. Accordingly, characteristic pyroptotic morphology was observed. Cells treated with 100 µg/mL CuZnONPs for 8 h began to display membrane blebbing, which enlarged over time, with most bubbles rupturing by 24 h (Figure 5b). Given that pyroptosis can be mediated by either the Caspase‐1/GSDMD or Caspase‐3/GSDME pathways, and considering the low expression of GSDME in 4T1 cells [42], we hypothesized the involvement of the Caspase‐1/GSDMD axis. Western blot analysis confirmed this, showing increased levels of cleaved Caspase‐1 (Cle‐Cas 1) and the N‐terminal fragment of GSDMD (GSDMD‐N) following CuZnONPs treatment (Figure 5c). To further ascertain the role of Zn^2+^ in pyroptosis induction, we compared the morphological effects of CuCl_2_, ZnCl_2_, and ZnONPs. After 24 h, cells treated with ZnCl_2_ or ZnONPs exhibited pronounced pyroptotic blebbing, whereas CuCl_2_ caused no significant morphological changes (Figure S27). These results collectively demonstrate that CuZnONPs‐induced pyroptosis is primarily mediated by the released Zn^2+^, consistent with reported mechanisms wherein Zn^2+^ elevates intracellular osmolarity and activates the Caspase‐1/GSDMD pathway [15].

Pyroptosis is known to promote the release of DAMPs, such as LDH, HMGB1, and calreticulin (CRT), which facilitate DCs maturation and subsequent T cell activation [43, 44]. As expected, we observed a concentration‐dependent increase in LDH release into the culture medium, reaching 4.2‐fold of the control at 100 µg/mL CuZnONPs (Figure 5d). Similarly, the concentrations of ATP (284.0 nM) and IL‐1β (62.1 pg/mL) in the culture medium at 100 µg/mL CuZnONPs were markedly higher than those in the control group (49.8 nM for ATP and 21.7 pg/mL for IL‐1β) (Figure S28). Immunofluorescence staining further revealed the translocation of HMGB1 from the nucleus to the extracellular space, with nearly complete loss of nuclear signal at 100 µg/mL of CuZnONPs. And the CRT expression showed a concentration‐dependent upregulation (Figure 5e). To evaluate the ensuing immunostimulatory outcome, we treated immature bone marrow‐derived dendritic cells (BMDCs) with conditioned medium from CuZnONPs‐treated 4T1 cells. Flow cytometry analysis showed that the maturation rate of BMDCs increased from 7.7% (control) to 11.7%, 14.0%, and 17.9% upon exposure to medium from cells treated with 25, 50, and 100 µg/mL CuZnONPs, respectively (Figure 5f and Figure S29). Together, these findings illustrate that the acidic TME triggers Zn^2+^ release from CuZnONPs, which activates the Caspase‐1/GSDMD‐mediated pyroptotic pathway, leading to DAMPs release and ultimately promoting DCs maturation and anti‐tumor immunity (Figure 5g).

### 2.7 In Vivo Therapeutic Effect by CuZnONPs

Given that pyroptosis‐induced immune activation can potentiate checkpoint blockade therapy [45], we evaluated the antitumor effect of CuZnONPs combined with the immune checkpoint inhibitor *α*PD‐L1 in a murine 4T1 breast cancer model. Tumor‐bearing mice were randomized into four groups (*n* = 5): (I) PBS, (II) αPD‐L1, (III) CuZnONPs, and (IV) CuZnONPs + αPD‐L1. As outlined in Figure S30a, mice received intravenous injections of CuZnONPs (10 mg/kg) on days 0 and 3, and αPD‐L1 (1 mg/kg) on days 1 and 4. The steady increase in body weight throughout the treatment period indicated good biosafety of CuZnONPs (Figure S30b).

Tumor volume measurements showed rapid growth in the PBS control group, which reached approximately 1200 mm^3^ by day 14. Monotherapy with αPD‐L1 only modestly suppressed tumor progression, whereas CuZnONPs alone or CuZnONPs + *α*PD‐L1 strongly inhibited tumor growth, with the combination group achieving the most profound inhibitory effect (Figure S30c,d). On day 14, tumors were resected and weighed (Figure S30e). The tumor inhibition rates were calculated as 32.8% for αPD‐L1, 61.0% for CuZnONPs, and 79.1% for the combination group (Figure S30f). To further evaluate the treatment effects at the histological level, tumor sections were stained with H&E, TUNEL, and Ki67 (Figure S30g). Ki67 staining revealed the highest extent of apoptosis in the combination group, followed by the CuZnONPs group, with αPD‐L1 alone showing minimal effect. Similarly, TUNEL assays confirmed the strongest suppression of tumor proliferation in the CuZnONPs + αPD‐L1 group. These results underscore the superior antitumor efficacy of CuZnONPs + αPD‐L1, highlighting the potential of CuZnONPs to synergize with immune checkpoint blockade.

### 2.8 In Vivo Immune Activation and Abscopal Effect

**FIGURE 6:**
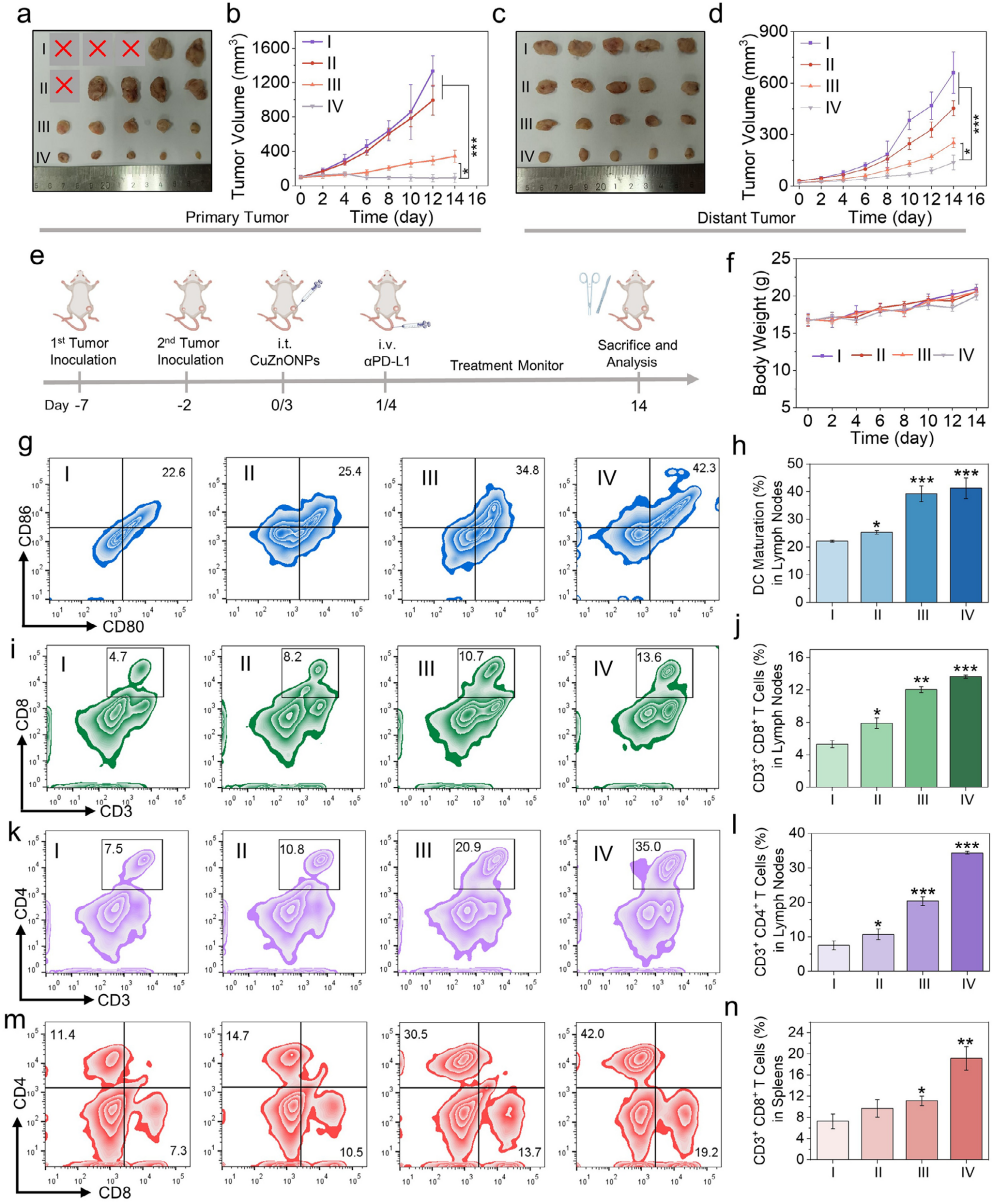
Digital photos and volume curves of (a, b) primary tumors and (c, d) distant tumors of mice receiving (I) PBS, (II) *α*PD‐L1, (III) CuZnONPs, and (IV) *α*PD‐L1 + CuZnONPs. Data are presented as mean ± SD (*n* = 5). (e) Schematic illustration showing the experimental procedure of the in vivo abscopal effect of CuZnONPs in a bilateral tumor model. (f) Body weights of mice during treatments. (g) Flow cytometric plots and (h) the corresponding quantitative analysis of DCs in lymph nodes. (i) Flow cytometric plots and (j) the corresponding quantitative analysis of CD8^+^ T cells in lymph nodes. (k) Flow cytometric plots and (l) the corresponding quantitative analysis of CD4^+^ T cells in lymph nodes. (m) Flow cytometric plots and (n) the corresponding quantitative analysis of CD4^+^ T and CD8^+^ T cells in spleens. Data are presented as mean ± SD (*n* = 3), and statistical significance was assessed by a one‐way ANOVA. ^*^ *p* < 0.05, ^**^ *p* < 0.01, and ^***^ *p* < 0.001.

Encouraged by the remarkable therapeutic efficacy of CuZnONPs + αPD‐L1, we established a bilateral tumor‐bearing model to investigate whether the combined treatment could elicit a systemic anti‐tumor immune response capable of controlling untreated distant tumors (Figure 6e). Mice were randomly assigned to four groups (*n* = 5) receiving intratumoral injections into the primary tumor as follows: (I) PBS, (II) αPD‐L1 (1 mg/kg), (III) CuZnONPs (10 mg/kg), and (IV) CuZnONPs + αPD‐L1. All treatments were well‐tolerated, as indicated by a steady increase in body weight throughout the study (Figure 6f). As expected, intratumoral administration more effectively suppressed primary tumor growth than intravenous injection, which is attributed to the localized delivery of a higher drug concentration (Figure 6a,b, Figure S30c,d). Importantly, significant growth inhibition was also observed in the distant, untreated tumor (Figure 6c,d, and Figure S31), suggesting that the local treatment triggered a systemic anti‐tumor immune response, manifested as an abscopal effect.

To elucidate the underlying immune mechanisms, the primary immunological organs, including lymph nodes and spleens from the treated mice, were collected and analyzed by flow cytometry. The results revealed a substantial increase in the proportion of DCs in the lymph nodes compared to the PBS control (Figure 6g,h). The average mature DC ratios in groups II, III, and IV were 25.3%, 39.2%, and 41.2%, respectively (Figure 6h). We next evaluated the activation of adaptive immunity. Both CD4^+^ T cells, which orchestrate immune responses [46], and CD8^+^ T cells, which directly mediate cytotoxicity [47], are essential for effective anti‐tumor immunity. Analysis of lymph nodes showed a considerable increase in the proportions of CD8^+^ T cells (Figure 6i,j) and CD4^+^ T cells (Figure 6k,l) across all treatment groups, with the most pronounced enhancement in the combination group (IV). Consistent with the observations in lymph nodes, a similar pattern of lymphocyte activation was observed in the spleens (Figure 6m,n, and Figure S32). The expression levels of the pyroptosis‐related cytokines IL‐1β and IFN‐γ in tumors were significantly elevated in groups III and IV (Figure S33). Additionally, the release of HMGB1 was observed in these two groups, confirming the immunogenic cell death induction by CuZnONPs (Figure S34). Cell pyroptosis, a typical form of inflammatory cell death, involves the release of large quantities of inflammatory cytokines. CuZnONPs induced pyroptosis in tumor cells, thereby activating immune responses and remodeling the tumor immune microenvironment. When combined with αPD‐L1 immunotherapy, these effects were further amplified, enhancing the therapeutic efficacy and promoting inflammatory responses. In summary, the combination of CuZnONPs and αPD‐L1 not only effectively inhibits primary tumor growth but also initiates a robust systemic immune response, leading to the suppression of distant tumors.

### 2.9 The Biosafety of CuZnONPs

We first assessed the biocompatibility of CuZnONPs using a hemolysis assay. The results showed that even at a high concentration of 400 µg/mL, the hemolysis rate remained as low as 3.5% (well below the 5% safety threshold), indicating the excellent hemocompatibility (Figure S35). To further evaluate the in vivo biosafety of CuZnONPs, we performed hematological analysis and histological examination. Blood biochemical indexes of mice treated with CuZnONPs (10 mg/kg) for 3 days all fell within normal limits (Figure S36). Consistent with this, H&E staining of main organs (heart, liver, spleen, lungs, and kidneys) showed no apparent morphological abnormalities across all treatment groups (Figure S37). These results jointly verify the high biocompatibility and favorable safety profile of CuZnONPs for therapeutic applications.

## 3 Conclusions

In conclusion, this study demonstrates the successful development of Cu‐doped ZnO_2_ nanoparticles (CuZnONPs) as a versatile nanoplatform for synergistic catalytic therapy and pyroptosis‐induced immunotherapy. The key mechanism involves a TME‐triggered self‐sustaining therapeutic cascade, where CuZnONPs autonomously generate H_2_O_2_ and release Zn^2+^ ions within the weakly acidic TME. DFT calculations and in vitro enzyme‐activity experiments confirmed that Cu doping played a pivotal role in conferring triple enzyme‐mimetic activities (CAT‐, POD‐, and OXD‐like), which collectively drove the catalytic conversion of H_2_O_2_ into O_2_, ·OH, and ·O_2_^−^. The resulting ROS storm followed concentration‐ and time‐dependent kinetics and effectively disrupted cellular redox homeostasis through GSH depletion and GPX4 suppression, ultimately causing comprehensive oxidative damage to lipids, mitochondria, and DNA. Importantly, the released Zn^2+^ ions specifically activated the Caspase‐1/GSDMD pyroptosis pathway, leading to the release of immunogenic DAMPs, including LDH, CRT, and HMGB1, which subsequently promoted DCs maturation. In vivo validation confirmed that CuZnONPs, synergized with αPD‐L1, not only suppress primary tumor growth but also elicited robust systemic anti‐tumor immunity through DCs maturation and T cell activation, resulting in effective control of distant tumors. This work presents a new bimetallic peroxide as a promising agent for cancer therapy and provides valuable design principles for developing metal peroxide‐based nanomedicines.

## Conflicts of Interest

The authors declare no conflicts of interest.

## Supporting information




**Supporting File**: advs75441‐sup‐0001‐SuppMat.docx.

## Data Availability

The data that support the findings of this study are available from the corresponding author upon reasonable request.
