# Supplementary material for: Self‐Accelerating Bimetallic Peroxide Nanozymes for Cascade‐Amplified Pyroptosis‐Immunotherapy
Source: Adv Sci (Weinh). 2026 Apr 23;13(40):e75441. doi: 10.1002/advs.75441 (PMC13335655; doi:10.1002/advs.75441)
Supplement: Supplementary file 1 — Supporting File: advs75441‐sup‐0001‐SuppMat.docx. [file ADVS-13-e75441-s001.docx]

Supporting Information

**Self-Accelerating Bimetallic Peroxide Nanozymes for Cascade-Amplified Pyroptosis-Immunotherapy**

Xuanyi Lu,^[a]^ Liang Li,^[a]^ Siyu Pan,^[a]^ Yuehan Jian,^[a]^ Yuhan Chen,^[a]^ Wenjie Yang, ^[a]^ Guang Song,*^[a]^ Xueyang Fang,*^[b]^ Ping’an Ma,*^[c]^ and Lijun Jiang*^[a]^

[a] Key Laboratory of Pesticide & Chemical Biology of Ministry of Education, Hubei Key Laboratory of Genetic Regulation and Integrative Biology, School of Life Sciences, Central China Normal University, Wuhan 430079, China

[b] MOE Key Laboratory of Laser Life Science & Institute of Laser Life Science, Guangdong Provincial Key Laboratory of Laser Life Science, College of Biophotonics, School of Optoelectronic Science and Engineering, South China Normal University, Guangzhou 510631, China

[c] Key Laboratory of Superlight Materials and Surface Technology, Ministry of Education, College of Materials Science and Chemical Engineering, Harbin Engineering University, Harbin 150001, China

E-mails: [lijunjiang@ccnu.edu.cn](mailto:lijunjiang@ccnu.edu.cn) (L. Jiang); [mapa675@ciac.ac.cn](mailto:mapa675@ciac.ac.cn) (P. Ma); [fangxueyang@scnu.edu.cn](mailto:fangxueyang@scnu.edu.cn) (X. Fang); [guangsong@ccnu.edu.cn](mailto:guangsong@ccnu.edu.cn) (G. Song)

EXPERIMENTAL SECTION

Materials and **Reagents.** Poly (vinylpyrrolidone) (PVP, Mw = 10 kDa), copric chloride dehydrate, 3,3’,5,5’-tetramethylbenzidine (TMB), zinc chloride, dihydrorhodamine 123 (DHR 123), skim milk, ammonium persulfate (APS), thiazolyl tetrazolium (MTT), and 5-carboxyfluorescein were purchased from Shanghai Macklin Biochemical Co., Ltd. Hydrogen peroxide (H_2_O_2_, 30%) was purchased from Sinopharm. Horseradish peroxidase (HRP), tris (4,7-diphenyl-1,10-phenanthroline) ruthenium(II) dichloride complex (Ru(dpp)_3_Cl_2_) zinc acetate and amplex red were purchased from Aladdin (China). 5,5’-dithiobis-2-(nitrobenzoicacid) (DTNB) was purchased from Energy Chemical (China). Singlet oxygen sensor green (SOSG) was purchased from Meilunbio (Dalian, China). 30% acrylamide-bisacrylamide solution (29:1) and 4% paraformaldehyde fix solution were purchased from Biosharp (China). HMGB1 rabbit monoclonal antibody, calreticulin rabbit monoclonal antibody, BCA protein assay kit, lipid peroxidation MDA assay kit, DNA damage assay kit, LDH cytotoxicity assay kit, actin-tracker red-rhodamine, 2-(4-Amidinophenyl)-6-indolecarbamidine dihydrochloride (DAPI), 3,3’-Dioctadecyloxacarbocyanine perchlorate (DIO), calcein acetoxymethyl ester (Calcein-AM) assay kit, glutathione peroxidase assay kit and annexin V-FITC/propidium iodide (PI) apoptosis detection kit were purchased from Beyotime Biotechnology Co., Ltd. (Shanghai, China). 2’,7’-dichlorofluorescein diacetate (DCFH-DA) and 5,5’,6,6’-tetrachloro-1,1’,3,3’-tetraethylbenzimidazolylcarbocyanine iodide (JC-1) were purchased from Adamas life (Shanghai, China). 1,2-bis(dimethylamino)ethane (TEMED), 5% BSA blocking buffer and triton X-100 were purchased form Solarbio (Beijing, China). Sodium dodecyl sulfate (SDS) was purchased from BioFroxx (Germany). Calreticulin polyclonal antibody, HRP-conjugated goat anti-rabbit IgG (H+L) and beta actin recombinant antibody were purchased from Proteintech (USA). GSDMD antibody and cleaved-caspase 1 antibody were purchased from Abmart (Shanghai, China). Animal-free recombinant murine GM-CSF and recombinant mutine IL-4 were purchased from Pepro Tech (USA). FITC anti-mouse CD3, APC anti-mouse CD4, PE anti-mouse CD8a, FITC anti-mouse CD11c, APC anti-mouse CD80 and PE anti-mouse CD86 were purchased from Biolegend (USA). Durvalumab (αPD-L1) was purchased from Bidepharm (China).

**Synthesis of ZnONPs.** Briefly, 0.2 g of PVP and 0.2 g of Zn(OAc)_2_ were premixed with 10 mL of Milli-Q water. Then, 1 mL of 30% H_2_O_2_ solution was added. The solution was then stirred at room temperature for 24 h. The ZnONPs product was collected through centrifugation (10000 × g, 10 min) and washed with Milli-Q water for several times. Finally, the product was redispersed in ethanol absolute.

**Synthesis of CuZnONPs****.** Cu-doping was carried out by a cation exchange method. Briefly, 10 mL of ethanol absolute solution of ZnONPs (25 mM of Zn) was mixed with 10 mL of ethanol absolute solution of CuCl_2_ (20 mM). Then, the reaction lasted for 4 h at room temperature under magnetic stirring. The CuZnONPs product was collected through centrifugation (10000 × g, 10 min) and washed with ethanol absolute for several times. Finally, the product was redispersed in Milli-Q water.

**Characterizations.** The nanoparticle morphologies and corresponding Energy Dispersive Spectroscopy (EDS) were acquired from a transmission electron microscope (TEM) (HT7800, HITACHI, Japan). The size of nanoparticles was measured using an instrument of dynamic light scattering (DLS) (Zetasizer Pro, Malvern, UK). UV-vis spectra were measured using a spectrophotometer (UV-2700i, Shimadzu, Japan). Fourier transforms infrared (FT-IR) spectra were recorded using a spectrometer (iS5, Thermo Fisher Scientific, America). X-ray photoelectron spectroscopy (XPS) spectra were obtained using a XPS equipment (ESCALAB Xi+, Thermo, America). The X-ray absorption of fine structures (XAFS) spectra at the Cu K-edge were measured at BL14W1 station in Shanghai Synchrotron Radiation Facility (SSRF). The Cu K-edge XAFS data were recorded in an X-ray mode. Electron spin resonance (ESR) measurements were carried out using a ESR spectrometer (EmxPlus, Bruker, Germany) at ambient temperature. Fluorescence spectrum was measured on a fluorescence spectrometer (F4700, HITACHI, Japan). Cytotoxicity assay was measured by an automated microplate reader (SynergyH1, Biotek, America). The confocal laser scanning microscope (CLSM) images of the samples were observed by CLSM imaging system (LSM 980, ZEISS, Germany). The cells after treatment were measured by flow Cytometry (BD FACSAria III, BD Biosciences, America). The Cu or Zn ions concentration was detected by inductively coupled plasma mass spectrometry (ICAP RQ, Thermo Fisher scientific, America). The X-ray diffraction (XRD) patterns of the powders were recorded on an X-ray powder diffractometer (D8 Advance, Bruker, Germany).

**Detection of H_2_O_2_ generation.** KMnO_4_ solution was exploited as a H_2_O_2_ indicator whose color changed from purplish red to colorless after specifically reacting with H_2_O_2_. Briefly, H_2_O_2_ (150 μL, 100 mM), ZnONPs (150 μL, 1.0 mg/mL) or CuZnONPs (150 μL, 1.0 mg/mL) was added into 1.35 mL of 0.1 M HCl solution. After 1.0 h of incubation at room temperature, the supernatants were collected by centrifugation (10000 × g, 10 min) and mixed with KMnO_4_ solution (75 μL, 1.0 mg/mL). Afterward, the UV-vis measurements at 400 to 700 nm were applied to confirm the generation of H_2_O_2_.

**pH-responsive release of Cu^2+^ or Zn^2+^ from CuZnONPs.** To detect the acid-induced release of Cu^2+^ and Zn^2+^, the CuZnONPs were dialyzed against PBS buffer solutions at a specified pH (7.4 or 5.5). The dialysates were collected at predetermined time points and the released Cu^2+^ or Zn^2+^ was detected by ICP-MS.

**Measurement of POD-like activity and kinetic assay of CuZnONPs.** To determine the generation of ·OH, the aqueous solution of CuZnONPs containing H_2_O_2_ was mixed with TMB solution. The absorbance change of TMB at approximately 652 nm was recorded using a UV-vis spectrophotometer. ·OH was also detected by ESR spectrometer. The steadystate kinetic analysis was examined based on the Michaelis-Menten curves at room temperature in PBS solution (pH 5.5) by monitoring the absorbance value at 652 nm every 10 s with CuZnONPs (100 μg/mL) as catalyst, TMB (0.1 mg/mL) as chromogenic agent and different concentrations of H_2_O_2_ (18.8, 31.3, 62.5, 125, and 250 mM) as substrate. The *K*_m_ and *V*_max_ were determined using the Lineweaver-Burk plot according to equations 1-3.

$A=\varepsilon lc$ (1)

$v_{0}=\frac{V_{max}[S]}{K_{m}+ [S]}$ (2)

$\frac{1}{v_{0}}=\frac{K_{m}}{V_{max}}\cdot\frac{1}{\left[ S \right]}+ \frac{1}{V_{max}}$ (3)

In these equations, ε is the molar absorption coefficient of oxTMB (39,000 M^−1^ cm^−1^), υ_0_ is the rate of conversion, *V*_max_ is the maximum rate of conversion, [S] is the concentration of substrate and *Κ*_m_ is the Michaelis constant. The Michaelis constant is equivalent to the substrate concentration at which the rate of conversion is half of *V*_max_. *K*_m_ indicates the affinity of the enzyme for the substrate: a lower *K*_m_ value means an excellent affinity.

**Measurement of OXD-like activity and kinetic assay of CuZnONPs.** The DHR 123 was used to determine the generation of ·O_2_^-^. The fluorescence of DHR 123 at 526 nm can be activated upon reacting with ·O_2_^-^. The aqueous solution of CuZnONPs containing H_2_O_2_ was mixed with DHR 123 solution. The change of fluorescence intensity of DHR 123 was observed in the presence of CuZnONPs. ·O_2_^-^ was also detected by ESR spectrometer. The steady-state kinetic analysis was examined via monitoring the absorbance value at 652 nm every 10 s with CuZnONPs (100 μg/mL) as catalyst and different concentrations of TMB (0.1, 0.2, 0.4, 0.6, 0.8 and 1.0 mM) as substrate at room temperature in PBS solution (pH 5.5) in the presence of H_2_O_2_ (100 mM).

**Measurement of CAT-like activity and kinetic assay of CuZnONPs.** To determine the generation of O_2_, dissolved O_2_ concentration was measured in aqueous solution of CuZnONPs containing H_2_O_2_ using an oxygen detector in 0.01 M PBS buffer (pH 5.5, 6.5, or 7.4). The kinetic assays of CuZnONPs with H_2_O_2_ as the substrate were performed at room temperature in PBS solution (pH 5.5) by adding CuZnONPs (final concentration of 100 μg/mL) and different amounts of H_2_O_2_ solution (final concentrations of 9.4, 18.8, 37.5, 75, and 150 mM). The O_2_ generation was recorded every 30 s.

**Cell culture.** Mouse breast cancer (4T1) cells were purchased from Wuhan Pricella Biotechnology Co., Ltd. And cultured in Roswell Park Memorial Institute (RPMI)-1640 culture medium (Procell) supplied with 10% fetal bovine serum (FBS) and 1.0% penicillin/streptomycin. The cells were incubated at 37°C in a humidified atmosphere containing 5% of CO_2_. The femurs and tibias were removed from 4 to 6 weeks old Balb/c mice and the bone-narrow cells were isolated. The bones marrow derived dendric cells (BMDCs) were incubated in 1640 medium containing 20 ng/mL GM-CSF and 10 ng/mL IL-4 for 6 days after lysis of erythrocytes, during which half of the media was replaced with fresh 1640 medium twice and cytokines were supplemented, then the BMDCs were harvested at the 7th day for further study.

**Cellular uptake of CuZnONPs**. 5-FAM (30 μL, 100 mM) was added into a dispersion of CuZnONPs (4 mL, 1.0 mg mL^-1^) and the mixtures were stirred for 24 h in the dark. Then the 5-FAM-labeled CuZnONPs (5-FAM-CuZnONPs) were obtained by centrifugation (10000 × g, 10 min) and washed with deionized water for further use. Uptake of the nanoparticles by 4T1 cells was evaluated via CLSM and flow cytometry, respectively.

For CLSM analysis, 4T1 cells (2.0 × 10^4^ cells) were first seeded into the confocal dishes and allowed to adhere overnight at 37°C. Next, the growth medium were replaced with a fresh one (1.0 mL) containing 5-FAM-CuZnONPs (50.0 μg/mL), and the cells were incubated in an incubator for 2.0 h. The treated cells were washed with PBS, fixed with 4.0% of paraformaldehyde for 15 min, permeabilized with 0.1% Triton X-100 for 5 min, blocked with 1.0% BSA for 30 min and then stained with DAPI for 5 min, followed by treatment with actin-tracker red-rhodamine solution for 20 min. Finally, the cells were observed by CLSM.

For flow cytometry analysis, 4T1 cells were first seeded into 12-well plates (5.0 × 10^4^ cells/well), and allowed to adhere overnight at 37°C in the normal incubator. The culture medium was then replaced with a fresh one (1.0 mL) containing 5-FAM-CuZnONPs (50.0 μg/mL). After incubation for 0.5, 1.0, 2.0 or 4.0 h, the cells were washed twice with PBS and harvested by trypsinization, followed by centrifugation (800 × g, 3 min). The obtained cells were re-suspended in PBS and analyzed using flow cytometry.

**Intracellular release of Zn^2+^.** A Zn^2+^-specific indicator, zinquin ethyl ester, was used to evaluate intracellular release of Zn^2+^. 4T1 cells (2.0 × 10^4^ cells) were first seeded into the confocal dishes and allowed to adhere overnight at 37°C. After incubation with CuZnONPs at the concentrations of 0, 25, 50, or 100 μg/mL for 4 h, the cells were washed with PBS, and then stained with 50 μM zinquin ethyl ester for 30 min. Finally, the cells were observed by CLSM.

**Cytotoxicity assay.** 4T1 cells were seeded into 96-well plates (3.0 × 10^3^ cells/well) and incubated for 24 h to adhere. The cells were treated with CuZnONPs at different concentrations (0, 5, 10, 20, 30, 40 50 or 100 μg/mL) for 24 h. Then, 10 μL of MTT solution (5.0 mg/mL) was added to each well. After an additional 4.0 h of incubation, the culture medium was removed, and then 100 μL of DMSO was added to each well. The absorbance was recorded at a wavelength of 490 nm using a microplate reader.

**Live/Dead cell staining.** 4T1 cells were seeded into confocal dishes and incubated for 24 h to adhere. Then, the cells were treated with CuZnONPs at different concentrations (0, 25, 50, or 100 μg/mL) for 12 h. Thereafter, the cells were co-stained with calcein-AM (5.0 μg/mL) and PI (10.0 μg/mL) for 30 min. The treated cells were rinsed with PBS and observed by CLSM.

**Flow cytometry analysis of the cell apoptosis.** 4T1 cells were seeded into 12-well plates and cultured for 24 h to adhere. Then, the cells were treated with CuZnONPs at different concentrations (0, 25, 50, or 100 μg/mL) for 24 h. The cells were then digested, and stained with 5.0 μL of PI and 10 μL of Annexin V-FITC in a binding buffer for 30 min. The apoptotic cells were quantified by flow cytometry, and analyzed using FlowJo software.

**The generation of intracellular H_2_O_2_.** The intracellular generation of H_2_O_2_ was determined by Amplex Red. When horseradish peroxidase (HRP) is present, Amplex Red reacts with H_2_O_2_ in a 1:1 chemical quantitative ratio to form highly fluorescent resorufin.

For CLSM analysis, 4T1 cells were seeded into confocal dishes (2.0 × 10^4^ cells/well), and incubated overnight. After that, the cells were treated with CuZnONPs at different concentrations (0, 25, 50, or 100 μg/mL) for 6 h. The cells were then washed with PBS, and treated with 20 μM Amplex Red and 20 μg/mL HRP for 30 min. Finally, the cells were observed by CLSM.

For flow cytometry analysis, 4T1 cells were first seeded into 12-well plates (5.0 × 10^4^ cells/well), and allowed to adhere overnight at 37°C in the normal incubator. The culture medium was then replaced with a fresh one (1.0 mL) containing CuZnONPs (0, 25, 50, or 100 μg/mL). After incubation for 6.0 h, the cells were washed twice with PBS, and treated with 20 μM Amplex Red and 20 μg/mL HRP for 30 min. The cells were harvested and re-suspended in PBS and analyzed using flow cytometry.

**The generation of intracellular ROS.** The intracellular generation of ROS was determined by a fluorogenic reagent DCFH-DA, which could be oxidized to the highly fluorescent dichlorofluorescein (DCF) by ROS.

For CLSM analysis, 4T1 cells were seeded into confocal dishes (2.0 × 10^4^ cells/well), and incubated overnight. Then the cells were treated with CuZnONPs at different concentrations (0, 25, 50, or 100 μg/mL) for 6 h. After that, the cells were washed with PBS, and then incubated with DCFH-DA for 30 min. The cells were washed three times with PBS, and then observed by CLSM.

For flow cytometry analysis, 4T1 cells were first seeded into 12-well plates (5.0 × 10^4^ cells/well), and allowed to adhere overnight at 37°C in the normal incubator. The culture medium was then replaced with a fresh one (1.0 mL) containing CuZnONPs (0, 25, 50, or 100 μg/mL). After incubation for 6.0 h, the cells were washed three times with PBS and treated with DCFH-DA for 30 min. The cells were harvested by trypsinization, followed by centrifugation (800 × g, 3 min). The obtained cells were re-suspended in PBS and analyzed using flow cytometry.

**Monitoring the CuZnONPs-induced changes of intracellular O_2_.** The intracellular O_2_ was determined by a fluorogenic reagfent, Ru(dpp)_3_Cl_2_. The fluorescence of the dye can be strongly reduced by molecular oxygen due to dynamic quenching

For CLSM analysis, 4T1 cells were seeded into confocal dishes (2.0 × 10^4^ cells/well), and incubated overnight. Then the cells were treated with CuZnONPs (100 μg/mL) for 0, 0.5, 1.0, or 2.0 h. After that, the cells were washed with PBS, and then incubated with Ru(dpp)_3_Cl_2_ (10 μM) for 20 min. The cells were washed three times with PBS, and then observed by CLSM.

For flow cytometry analysis, 4T1 cells were first seeded into 12-well plates (5.0 × 10^4^ cells/well), and allowed to adhere overnight at 37°C in the normal incubator. The culture medium was then replaced with a fresh one (1.0 mL) containing CuZnONPs (100 μg/mL). After incubation for 0, 0.5, 1.0 or 2.0 h, the cells were washed twice with PBS and treated with Ru(dpp)_3_Cl_2_ (10 μM) for 20 min. The cells were harvested by trypsinization, followed by centrifugation (800 × g, 3 min). The obtained cells were re-suspended in PBS and analyzed using flow cytometry.

**Monitoring the CuZnONPs-induced changes of intracellular ·OH.** 4T1 cells were seeded into confocal dishes (2.0 × 10^4^ cells/well), and incubated overnight. Then the cells were treated with CuZnONPs at different concentrations (0, 25, 50, or 100 μg/mL) for 2 h. After that, the cells were washed with PBS, and then incubated with HPF (10 μM) for 30 min. The cells were washed three times with PBS, and then observed by CLSM.

**Monitoring the CuZnONPs-induced changes of intracellular ·O_2_^-^.** 4T1 cells were seeded into confocal dishes (2.0 × 10^4^ cells/well), and incubated overnight. Then the cells were treated with CuZnONPs at different concentrations (0, 25, 50, or 100 μg/mL) for 6 h. After that, the cells were washed with PBS, and then incubated with DHR 123 (10 μM) for 30 min. The cells were washed three times with PBS, and then observed by CLSM.

**Detection of DNA Damage.** γ-H2AX immunofluorescence analysis was used for the detection of DNA damage. Typically, 4T1 cells were seeded into confocal dishes and incubated for 24 h to adhere. The cells were then treated with CuZnONPs (0, 25, 50, or 100 μg/mL) for 12 h. The cells were washed with PBS, fixed with 4.0% paraformaldehyde for 20 min, permeabilized with 0.1% Triton X-100 for 5 min, and blocked with 1.0% BSA for 30 min in sequence. The obtained cells were incubated with γ-H2AX rabbit monoclonal antibody (dilution 1: 500) overnight at 4°C, and further stained with FITC-conjugated goat anti-rabbit secondary antibody for 1.0 h at room temperature. After washing for several times with PBS, the cell nucleus was stained with DAPI for 5 min. Finally, the fluorescence images were taken by the CLSM.

**Monitoring the changes of mitochondrial membrane potential.** 4T1 cells were seeded into confocal dishes and incubated for 24 h to adhere. The cells were then treated with CuZnONPs (0, 25, 50, or 100 μg/mL) for 6 h, respectively. Afterwards, the cells were stained by JC-1 (10 μg mL^-1^) and imaged via CLSM (JC-1 monomers, Ex = 514 nm, Em = 529 nm; JC-1 aggregates, Ex = 585 nm, Em = 590 nm).

**Observation of cell membrane morphology.** 4T1 cells were pre-seeded into confocal dishes and cultured for 24 h. After that, the cells were treated with CuZnONPs (0, 25, 50, or 100 μg/mL) for 6 h, respectively. The treated cells were washed with PBS, fixed with 4.0% paraformaldehyde for 15 min, permeabilized with 0.1% Triton X-100 for 5 min, blocked with 1.0% BSA for 30 min and then stained with DAPI for 5 min, followed by treatment with DIO (10 μg/mL) for 20 min. Finally, the cells were observed by CLSM.

**Immunofluorescence analysis of GPX4 protein in cells.** 4T1 cells were seeded into confocal dishes and cultured for 24 h. Next, the cells were treated with CuZnONPs (0, 25, 50, or 100 μg/mL) for 12 h. Then, the cells were fixed with 4.0% polyoxymethylene for 20 min, permeabilized with 0.1% Triton X-100 for 5 min,1% BSA for 30 min, and then incubated with Cy3-labled GPX4 rabbit monoclonal antibody (dilution 1: 500) at 4°C overnight. The nucleus was stained by DAPI for 15 min. Finally, the samples were observed by CLSM.

**Assay of intracellular GSH**. 4T1 cells were seeded into 6-well plates at a density of 2×10^5^ cells per well, and cultured for 24 h. Then, the cells were treated with CuZnONPs at different concentrations (0, 25, 50, or 100 μg/mL) for 6.0 h, respectively. After 6.0 h of incubation at 37°C, the culture media was removed, and the cells were washed three times with PBS. Subsequently, the cells were lysed with 40 μL of RIPA Lysis Buffer, and centrifuged (12000 × g, 5 min). The supernatant (100 μL) of centrifuged lysis buffer was then mixed with 100 μL of DTNB (400 μM). After 30 min of incubation at room temperature, the content of cellular GSH was measured at 412 nm by a multi-mode microplate reader. The percentage content of GSH was calculated based on the comparison to the GSH content of untreated cells.

**Assay of intracellular MDA and GPX4**. 4T1 cells were seeded into 6-well plates and cultured for 24 h. Then, the cells were treated with CuZnONPs at different concentrations (0, 25, 50, or 100 μg/mL) for 12 h, respectively. The GSH level and GPX4 activity were respectively detected using an MDA Assay Kit and a Glutathione Peroxidase Assay Kit according to the manufacturer’s instructions.

**Cell morphological change.** To evaluate whether CuZnONPs can induce cell swelling with large bubbles, 4T1 cells were seeded into a 12-well plate at a density of 5.0×10^5^ cells per well in 1.0 mL of medium and cultured for 24 h. Then, the medium was replaced with 1.0 mL of fresh medium containing CuCl_2_ (87.1 μM), ZnCl_2_ (271.1 μM), ZnONPs (50 μg/mL) or CuZnONPs (50 μg/mL). After incubation for 8, 12 or 24 h, the cells were imaged by microscope.

**Immunofluorescence analysis of HMGB1 in cells.** 4T1 cells were seeded into confocal dishes and cultured for 24 h. Next, the cells were treated with CuZnONPs (0, 25, 50, or 100 μg/mL) for 12 h. Then, the cells were fixed with 4.0% polyoxymethylene for 20 min, permeabilized with 0.1% Triton X-100 for 5 min, 1% BSA for 30 min, incubated with HMGB1 rabbit monoclonal antibody (dilution 1: 500) at 4°C overnight and further stained with Cy3-conjugated goat anti-rabbit secondary antibody for 1.0 h at room temperature. The nucleus was stained by DAPI for 5 min. Finally, the samples were observed by CLSM.

**Immunofluorescence analysis of CRT in cells.** 4T1 cells were seeded into confocal dishes and cultured for 24 h. Next, the cells were treated with CuZnONPs (0, 25, 50, or 100 μg/mL) for 12 h. Then, the cells were fixed with 4.0% polyoxymethylene for 15 min, permeabilized with 0.1% Triton X-100 for 5 min, 1% BSA for 30 min, and then incubated with calreticulin rabbit monoclonal antibody solution (dilution 1: 500) at 4°C overnight and further stained with FITC-conjugated goat anti-rabbit secondary antibody for 1.0 h at room temperature. The nucleus was stained by DAPI for 15 min. Finally, the samples were observed by CLSM.

**Determination of extracellular LDH, ATP, or IL-1β.** 4T1 cells were seeded into 12-well plates and cultured for 24 h. Then, the cells were treated with CuZnONPs at different concentrations (0, 25, 50, or 100 μg/mL) for 12 h, respectively. The LDH, ATP**,** or IL-1β levels in supernatant of culture medium were detected using Assay Kits according to the manufacturer’s instructions.

**FC analysis of the maturation of BMDCs.** 2.0 mL of 4T1 cells in complete medium were seeded into 6 well-plates at a density of 2.0 × 10^6^ cells/mL, and allowed to adhere at 37°C for 24 h. After that, the culture media was replaced with 2.0 mL of fresh one containing CuZnONPs (0, 25, 50, or 100 μg/mL). After 4.0 h of incubation, the culture medium containing nanoparticles was replaced with 2.0 mL of complete 1640 medium. After 24 h incubation at 37°C, the supernatant of 4T1 cells in each group was used as the conditioned medium. The BMDCs were seeded in 6 well-plates at a density of 2.0 × 10^6^ cells per well. After 24 h incubation at 37°C, they were cultured with 50% conditioned medium and 50% completed 1640 medium for another 24 h at 37°C. After that, the BMDCs were collected, stained with FITC anti-mouse CD11c, APC anti-mouse CD80 and PE anti-mouse CD86 and measured by flow cytometry.

**Western blot.** 4T1 cells were seeded into 6-well plates at a density of 2.0 × 10^5^ cells per well in complete growth medium, and allowed to adhere at 37°C for 24 h. The growth medium was replaced with fresh one (2.0 mL, without FBS) containing CuZnONPs (0, 25, 50, or 100 μg/mL). After 24 h of incubation, the cells in all dishes were washed with cold PBS. After that, the cells in each well were treated with 100 μL of RIPA lysis buffer (with PMSF) at 0°C for 30 min, and the lysate was collected and centrifuged (12000 × g, 20 min, 4.0°C). The supernatant after centrifugation was quantified for protein concentration by a BCA assay Kit. RIPA lysis buffer and loading buffer were then added to dilute the protein of each group to a uniform concentration, followed by boiling in a metal bath (100°C, 10 min). SDS-PAGE gel (12%) was taken advantage for electrophoretic separation of various protein samples. The separated protein samples on the gel were then transferred to a PVDF membrane (0.22 μm). Subsequently, the membrane was blocked with 5.0% of skim milk solution for 1.0 h. The membrane was washed three times with tris-buffered saline with tween 20 (TBST), and incubated overnight at 4.0°C with the corresponding mouse primary antibody (Cle-Caspase 1, GSDMD, GAPDH, or Beta Actin) on a shaker, then washed by TBST. After that, the membrane was incubated with a goat anti-mouse secondary antibody at room temperature for 1.0 h, then washed with TBST. Finally, the protein bands were visualized by an enhanced chemiluminescence (ECL) detection reagent in a darkroom.

**Tumor model.** All animal experiments were performed in compliance with the relevant laws and institutional guidelines and were approved by the Animal Ethical Experimentation Committee of Central China Normal University (approval No. CCNU-IACUC-2023-018). Female Balb/c mice (5 weeks old, 15 ~ 20 g) were purchased from the Experimental Animal Center of Hubei Provincial Center for Disease Control and Prevention (Wuhan, China). For subcutaneous tumors: the 4T1 tumor-bearing mice were prepared by subcutaneous inoculation of 4T1 cells (2.0 × 10^6^ cells in 100 μL PBS) into the right leg of each mouse.

**Subcutaneous tumor therapeutic performance.** When the tumor volume reached about 50-100 mm^3^, 4T1 tumor-bearing mice were randomly divided into four groups for treatments. 100 μL of PBS, αPD-L1 (dosage: 1.0 mg/kg), CuZnONPs (dosage: 10 mg/kg), or αPD-L1 + CuZnONPs (αPD-L1 dosage: 1.0 mg/kg, CuZnONPs dosage: 10 mg/kg) were i.v. injected into the 4T1 tumor-bearing mice at the day 0, or 3, respectively. Body weights and tumor volumes of mice in each group were recorded every other day during the treatment (the total tumor volume should be smaller than 1500 mm^3^). The tumor volume was calculated referring to the equation: Volume = (length × width^2^)/2. At the end of the study, tumors were excised and weighed to calculate the tumor inhibition rate, determined as (W_0_ – W_1_) / W_0_ × 100%. W_0_ and W_1_ represent the mean tumor weights of the control and treatment groups, respectively.

For H&E, TUNEL and Ki67 analysis: tumor masses were weighed, photographed, fixed in 4.0% paraformaldehyde, embedded in paraffin, sectioned, stained with hematoxylin and eosin (H&E), ki67, and terminal deoxynucleotidyl transferase (TdT)-mediated deoxyuridine triphosphate (dUTP)-biotin nick-end labeling (TUNEL).

**In vivo antitumor therapeutic effect in double tumor-bearing mouse model.** The 4T1 double tumor-bearing animal model was established by subcutaneously inoculating 2 × 10^6^ 4T1 cells per mouse onto the right flank of Balb/c mice on day -7 and onto the left flank on day -2, respectively. The tumor on the right side was used as a primary tumor for therapeutics treatment, and the left tumor was designed as a distant tumor to evaluate the antitumor immunologic effect. When the size of right tumors reached about 100 mm^3^, the mice were randomly divided into 4 groups (n = 5): (1) PBS, (2) αPD-L1 (dosage: 1.0 mg/kg), (3) CuZnONPs (dosage: 10 mg/kg) and (4) CuZnONPs + αPD-L1 (αPD-L1 dosage: 1.0 mg/kg, CuZnONPs dosage: 10 mg/kg). Then 100 μL of the formulations were intratumorally injected into the primary tumor, followed by intravenous injection of αPD-L1 (1 mg/kg). The tumor sizes and mice’s body weights were recorded every other day. At the end of treatment, mice organs were used for other relevant experiments.

**Anti-tumor immune responses.** For the lymph nodes: the lymph nodes were ground and filtered through a 70 μm of filter membrane, and washed with PBS by centrifugation (150 × g, 5 min). Subsequently, the cells were stained with fluorescent antibodies (FITC anti-CD11c, APC anti-CD80, PE anti-CD86 for DCs; FITC anti-CD3, APC anti-CD4, PE anti-CD8a for T cells), and flow cytometry was utilized to analyze the percentage of immune cells within the lymph nodes, including dendritic cells (CD11c^+^, CD80^+^, CD86^+^), cytotoxic T cells (CD8^+^ T cells, CD3^+^, CD8^+^), and helper T cells (CD4^+^ T cells, CD3^+^, CD4^+^).

For spleen tissues: the spleen tissues were lysed with RBC lysis buffer, filtered through a 70 μm of filter membrane, and washed with Hanks’ solution by centrifugation (150 × g, 5 min). The cells were then stained with fluorescent antibodies (FITC anti-CD3, APC anti-CD4, PE anti-CD8a) and analyzed by flow cytometry to determine the percentages of CD4^+^ (CD3^+^, CD4^+^) T cells and CD8^+^ (CD3^+^, CD8^+^) T cells for assessment of the systemic immune level of splenic infiltrating T cells.

For tumor tissues: tumor tissues stored in liquid nitrogen were placed in pre‑chilled PBS containing protease inhibitors and thoroughly homogenized. The homogenate was centrifuged at 5000 × g for 10 min at 4°C, and the supernatant was collected. Concentrations of IL-1β and IFN-γ in the tumor tissue supernatants were quantified using ELISA kits according to the manufacturer’s instructions. A portion of the tumor tissues were fixed in 4% paraformaldehyde solution for paraffin sectioning, followed by immunofluorescence staining for HMGB1 antibody and imaging by CLSM.

**Hemolysis assay.** Fresh blood collected from BALB/c mice was centrifuged to isolate red blood cells (RBCs) (250 × g, 10 min). The supernatant was carefully removed, and the RBC pellet was washed with PBS until the supernatant became clear. The obtained RBCs were then diluted with PBS to a final concentration of 4.0% (v/v). CuZnONPs were prepared at concentrations of 100, 200, 400, and 800 μg/mL in PBS. Subsequently, 500 μL of each CuZnONPs suspension was mixed with 500 μL of the diluted RBCs, and the mixtures were incubated in a water bath at 37 ± 0.5 °C for 4 h. RBCs treated with H_2_O and PBS (pH 7.4) under the same conditions served as the positive and negative controls, respectively. After incubation, the samples were centrifuged to remove intact RBCs (250 × g, 10 min). Then, 100 μL of the supernatant from each sample was transferred to a 96-well plate, and the absorbance was measured at 540 nm using a microplate reader. The hemolysis percentage was calculated using the following formula: Hemolysis (%) = (A_sample_ − A_negative control_) / (A_positive control_ − A_negative control_) × 100%. All experiments were performed in triplicate for each concentration.

**Biosafety evaluation.** 100 μL of PBS, or CuZnONPs (dosage: 10 mg/kg) was i.v. injected into the healthy Balb/c mice. After 3 days, the blood samples were collected and centrifuged at 3000 × g for 15 min. 300 μL of the upper serum was taken out to determine the hematology indicators, i.e., white blood cell count (WBC), red blood cell count (RBC), hematocrit (HCT), hemoglobin concentration (MCHC), lymphocyte percentage (Lym), and mean corpuscular volume (MCV).

For the major organs toxicity detection, the mice were sacrificed at 3 days post-injection, and the major organs (heart, liver, spleen, lung, and kidney) were collected. Then, the H&E staining method was used to observe and analyze the sections of major organs.

**Statistical analysis.** For the statistical analysis, we used SPSS 25.0 (SPSS, Chicago, USA). All experiments were performed at least in triplicate and all experimental data are presented as Mean ± SD. Two groups were compared using Student’s t-test, and more than two groups were compared using one-way analysis of variance (ANOVA) followed by a post hoc Tukey’s test. The significance level was fixed as **P* < 0.05, ***P* < 0.01, or ****P* < 0.001.


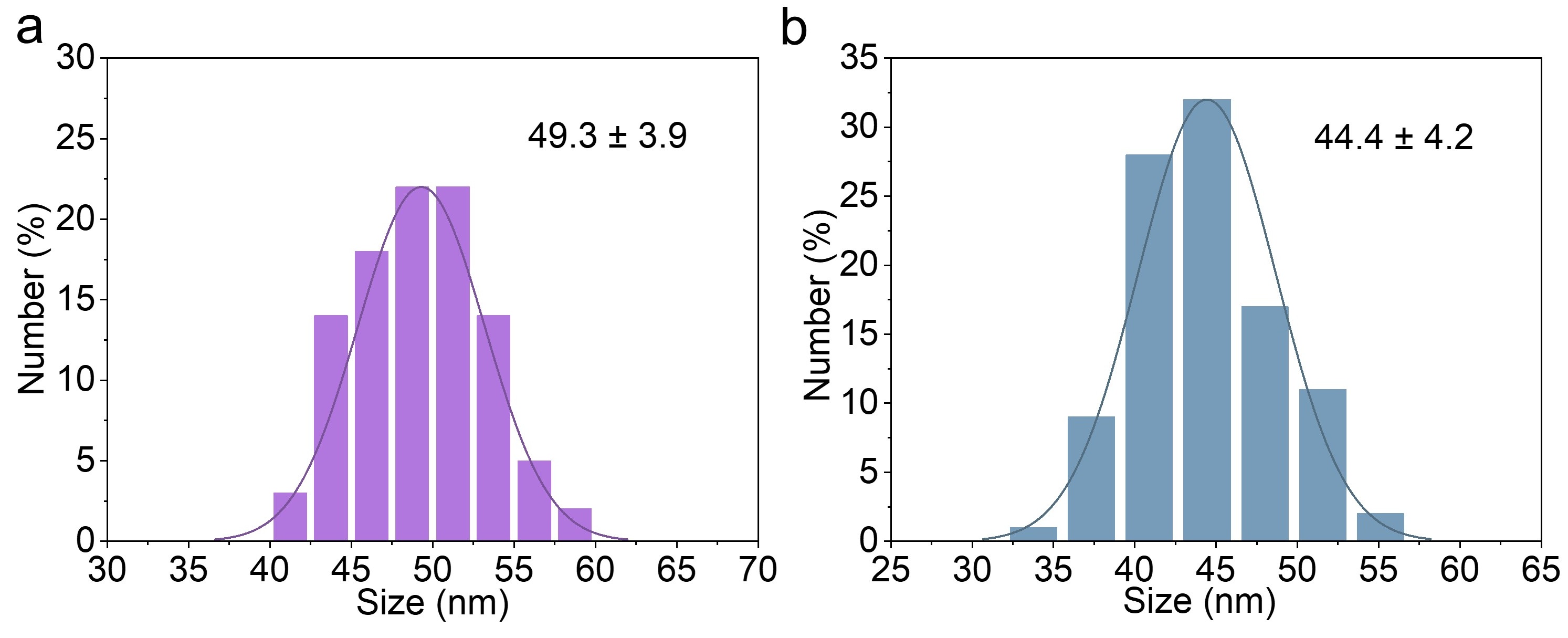


**Figure S1.** Size distributions of (a) ZnONPs and (b) CuZnONPs from TEM images in Figure 1a and Figure 1b, respectively.


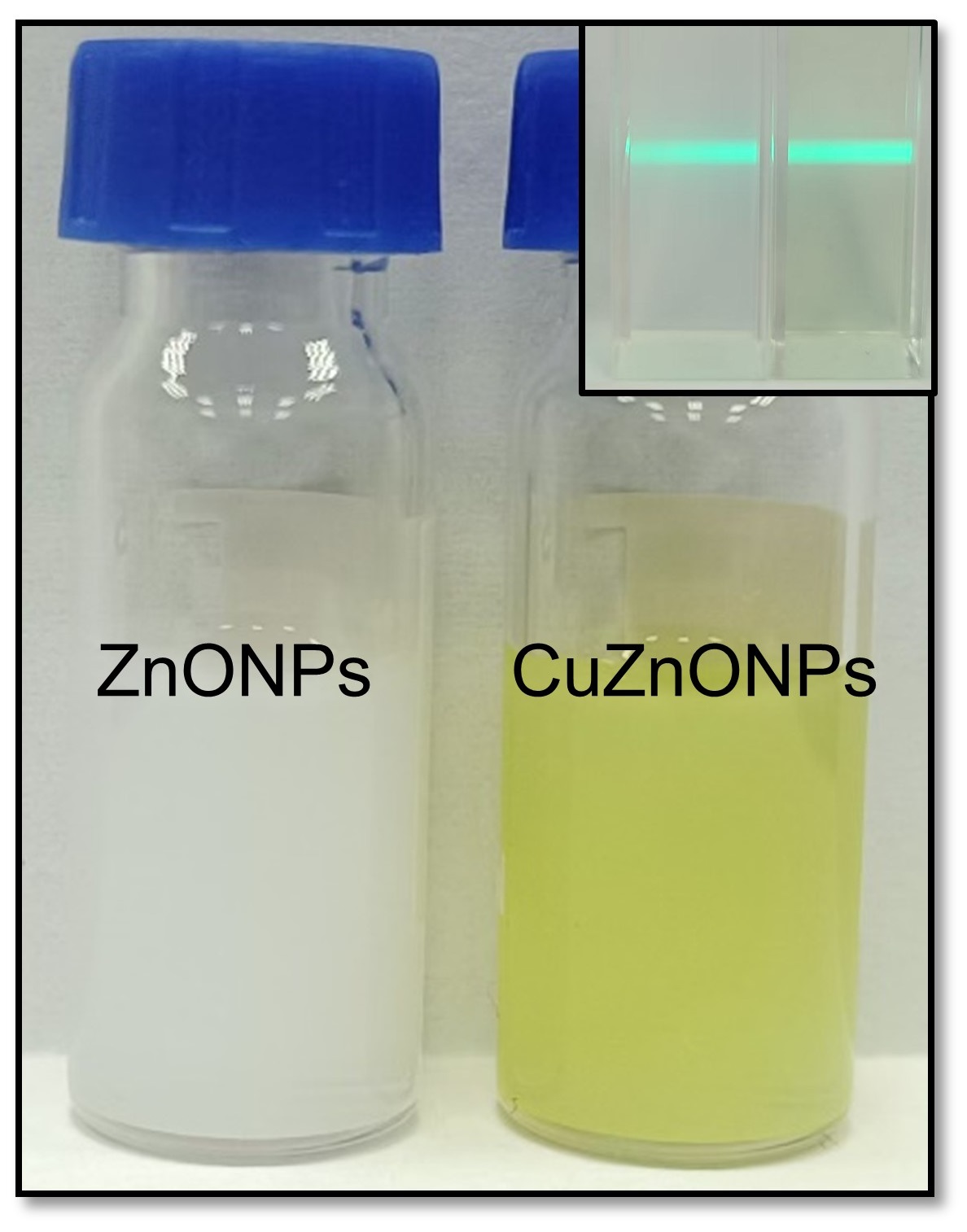


**Figure S2.** Photos of ZnONPs and CuZnONPs solutions. Inset showing the Tyndall effect of ZnONPs and CuZnONPs dispersions.


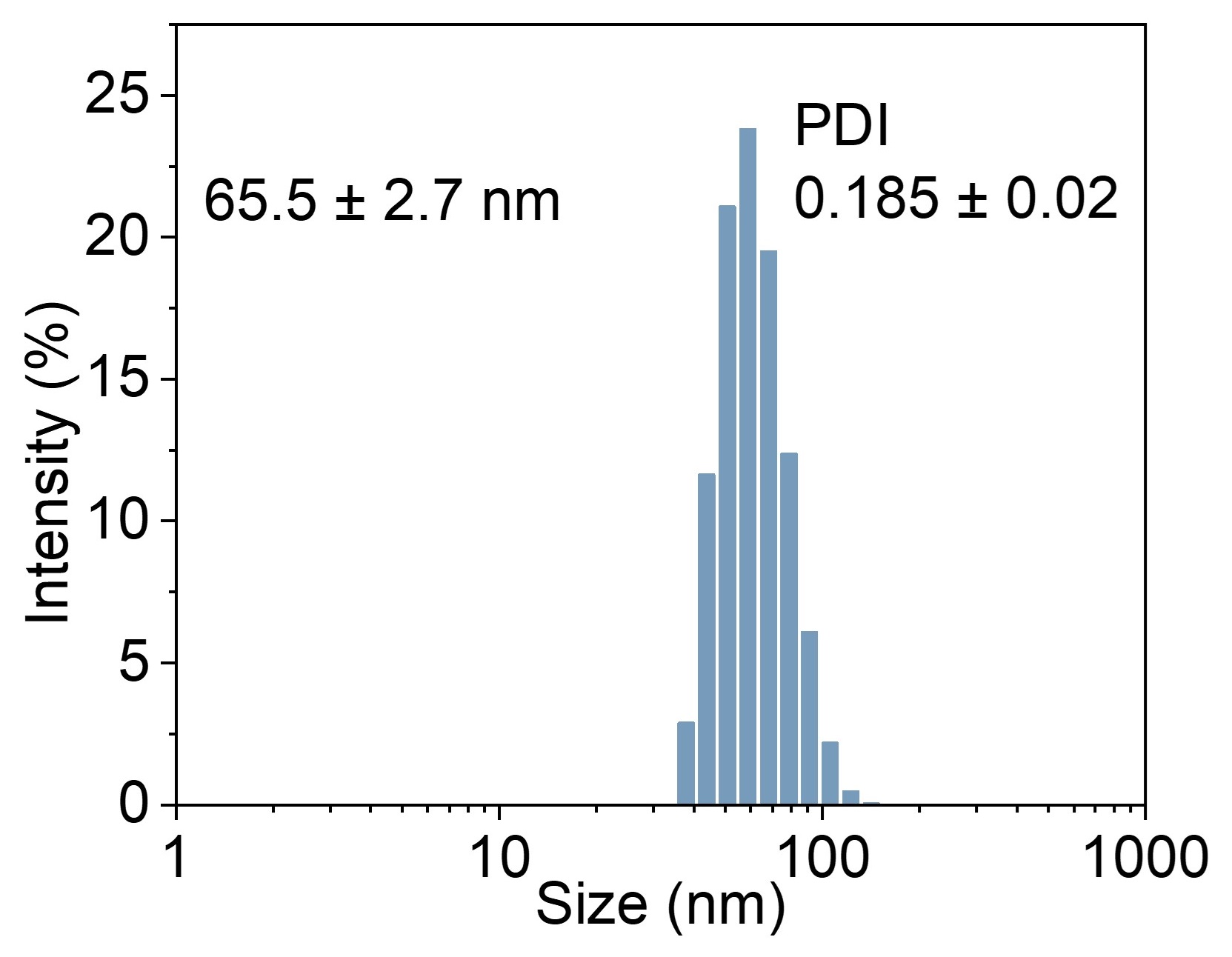


**Figure S3.** Dynamic light scattering size distributions of CuZnONPs.


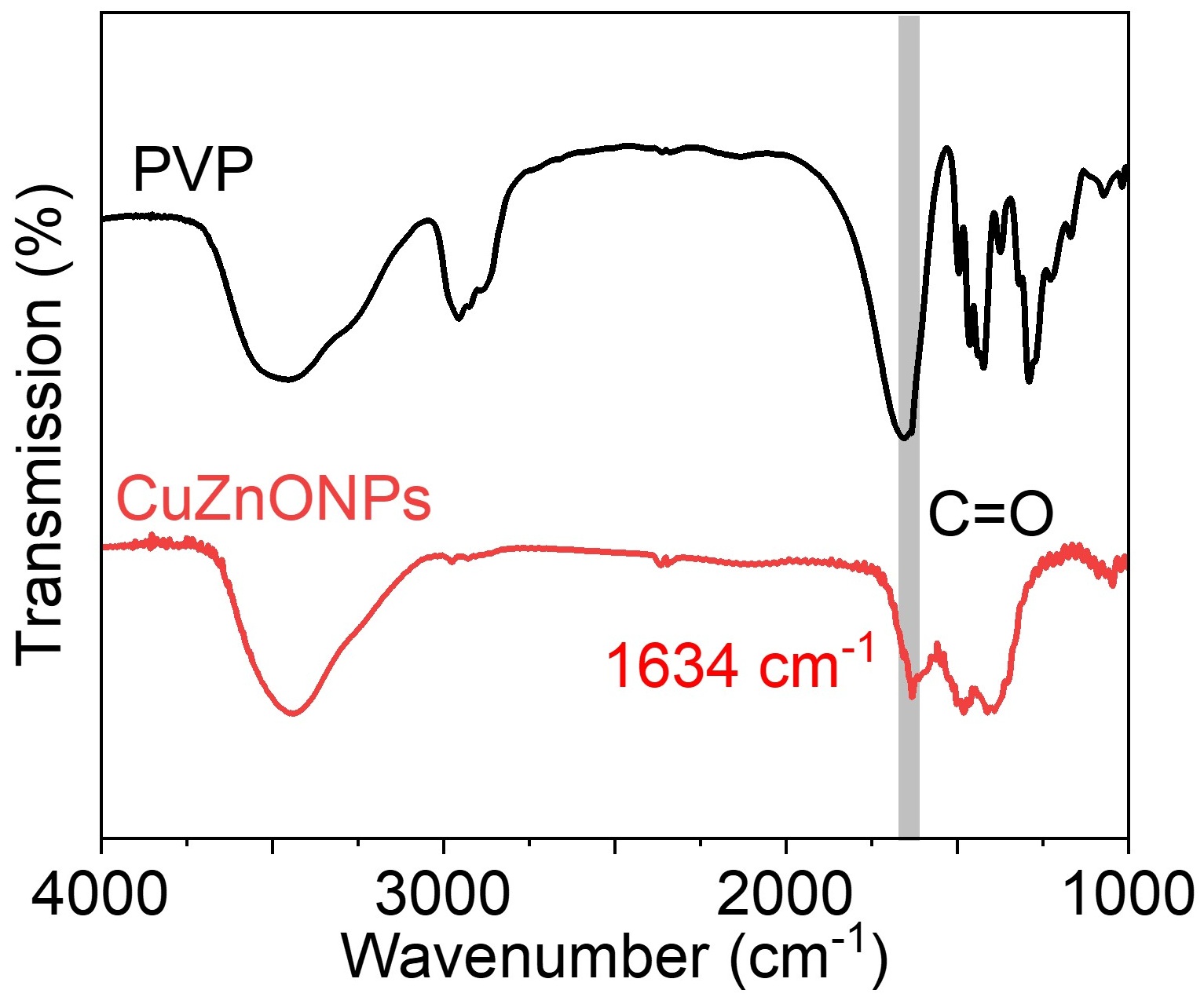


**Figure S4.** FTIR spectra of PVP and CuZnONPs.


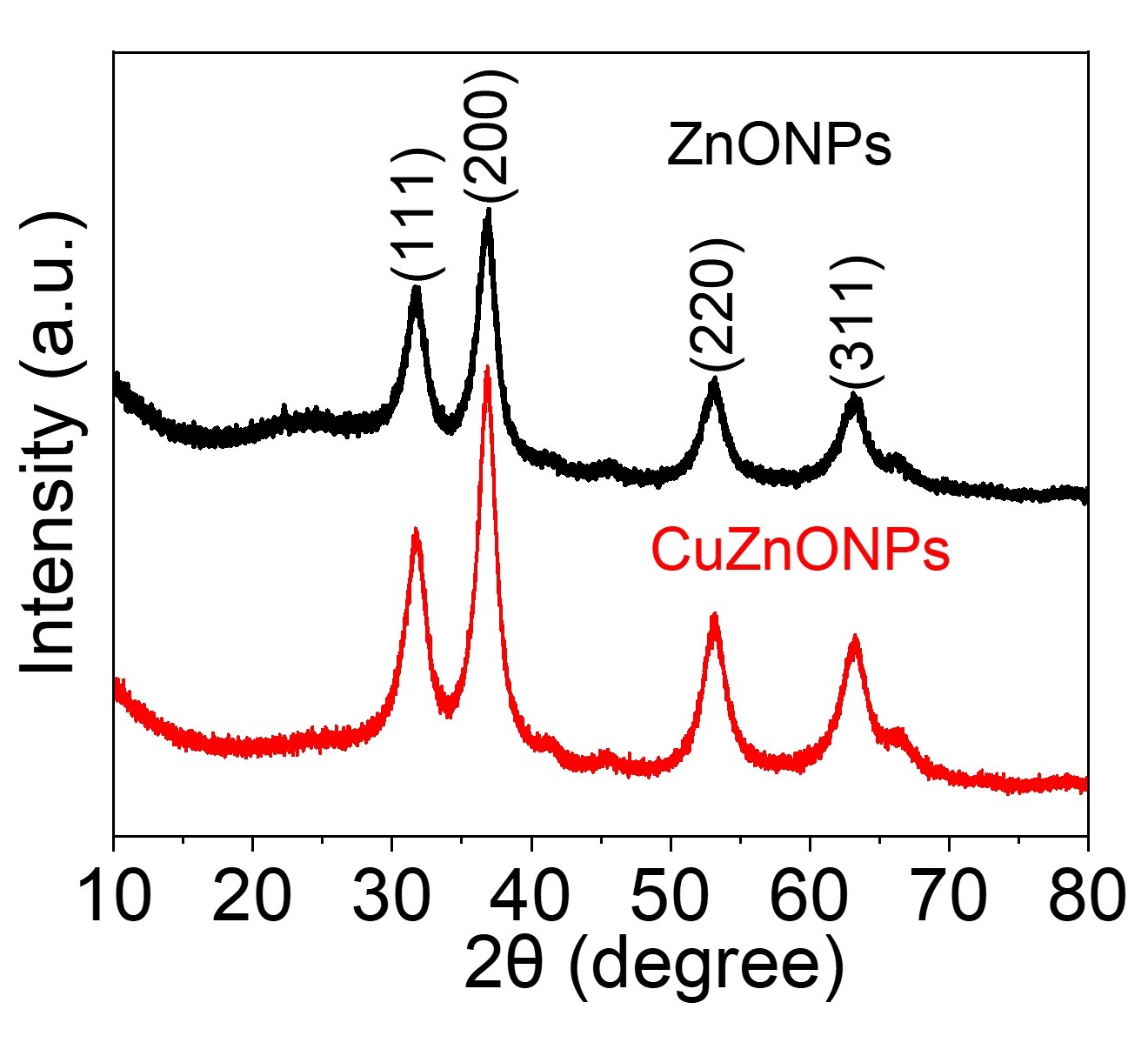


**Figure S5.** XRD spectra of ZnONPs and CuZnONPs.

**Table S1.** Hydrodynamic diameter and zeta potential of CuZnONPs stored in D. I. water, PBS, and cell culture medium with 10% FBS at 4°C for 0, 7, and 30 days, respectively.

| Solution | Storage (days) | Photo | Size (nm) | Zeta Potential |
| --- | --- | --- | --- | --- |
| D. I. Water | 0 | 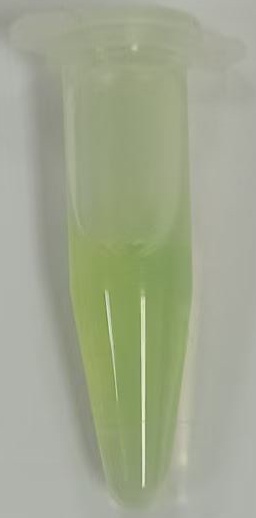 | 62.3 ± 1.1 | 31.0 ± 1.1 |
|  | 7 | 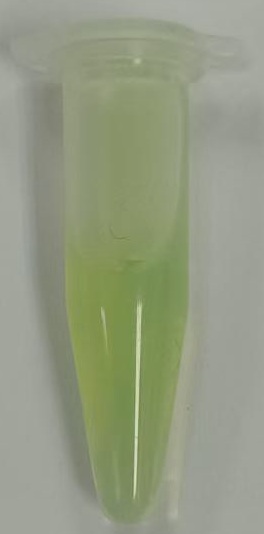 | 62.0 ± 0.9 | 30.2 ± 1.5 |
|  | 30 | 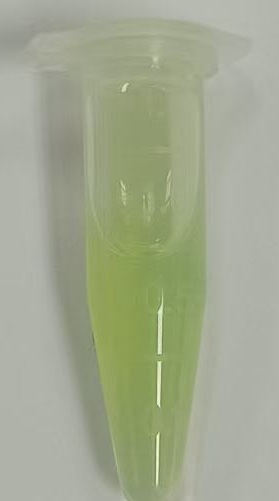 | 61.6 ± 0.3 | 32.1 ± 1.4 |
| PBS | 0 | 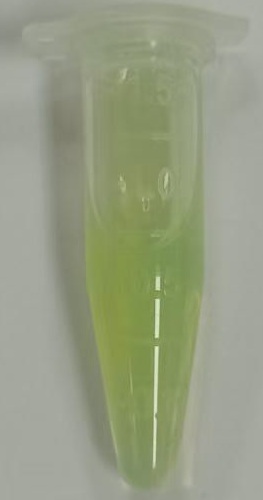 | 67.0 ± 0.4 | 31.1 ± 0.4 |
|  | 7 | 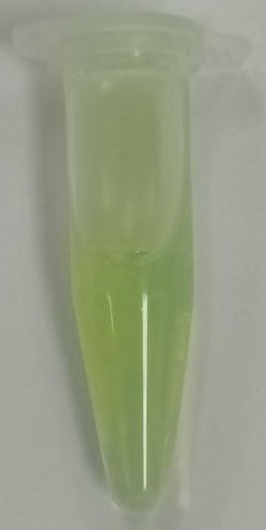 | 62.7 ± 4.0 | 32.5 ± 1.2 |
|  | 30 | 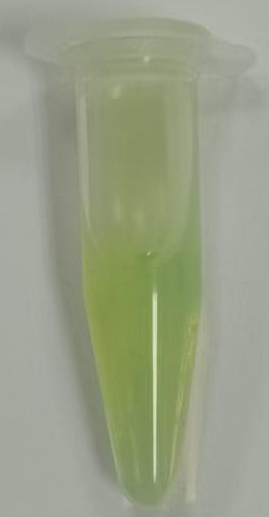 | 65.5 ± 2.7 | 31.2 ± 2.6 |
| Media with  10% FBS | 0 | 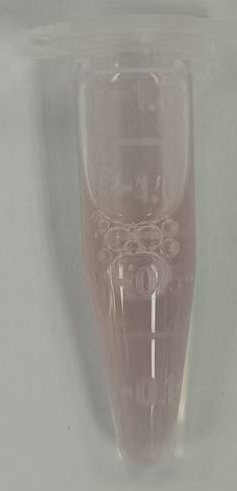 | 66.9 ± 0.1 | 28.9 ± 1.3 |
|  | 7 | 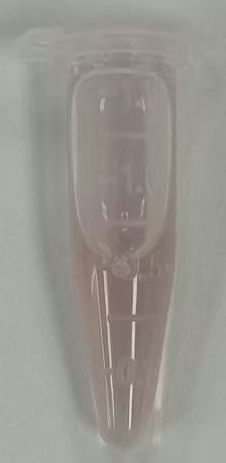 | 73.2 ± 3.2 | 27.2 ± 0.5 |
|  | 30 | 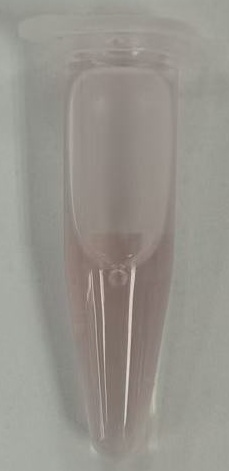 | 75.1 ± 0.8 | 26.7 ± 0.8 |


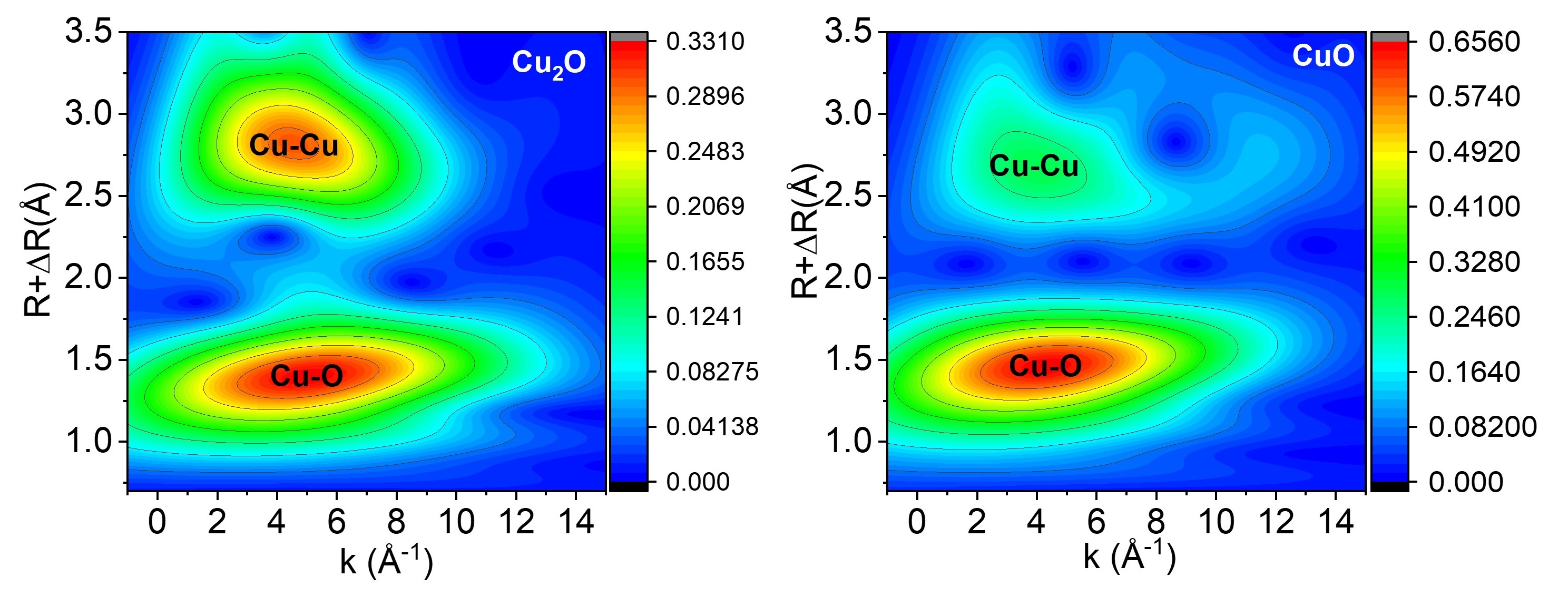


**Figure S6.** Wavelet transform of Cu K-edge EXAFS of Cu_2_O and CuO.


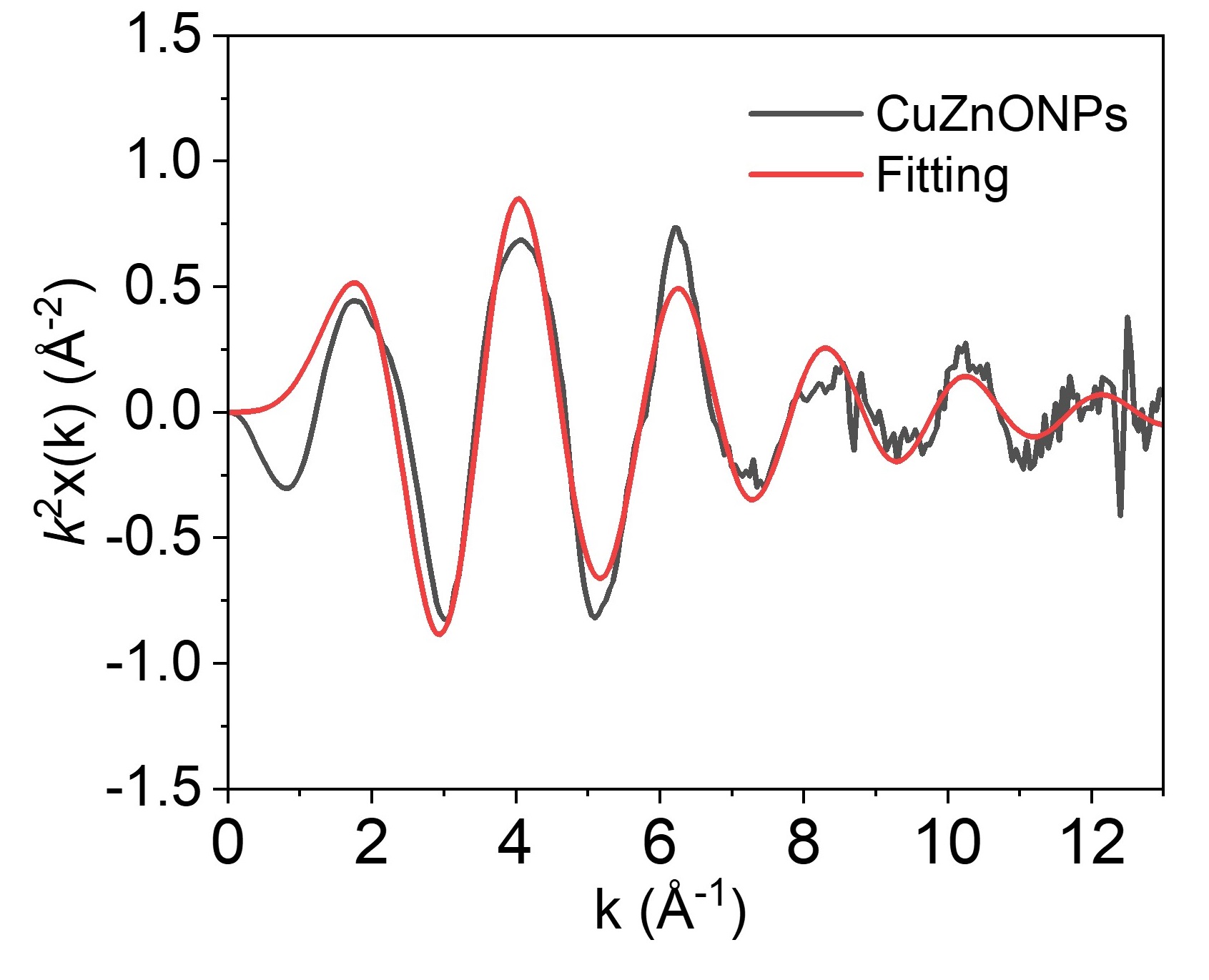


**Figure S7.** EXAFS fitting curve of CuZnONPs at k space.

**Table S2. EXAFS fitting parameters at the Cu K-edge for CuZnONPs (S_0_^2^ = 0.87).**

| Sample | Path | N | R(Å) | σ^2^×10^3^(Å^2^) | ΔE(eV) | R factor |
| --- | --- | --- | --- | --- | --- | --- |
| CuZnONPs | Cu-O | 3.4 ± 0.4 | 1.95 ± 0.010 | 5.9 ± 1.7 | -2.9 ± 1.6 | 0.008 |
| Cu foil | Cu-Cu | 12 | 2.54 ± 0.003 | 8.6 ± 0.4 | 4.5 ± 0.5 | 0.003 |
| CuO | Cu-O | 4.3 ± 0.4 | 1.95 ± 0.008 | 5.9 ± 1.7 | 5.0 ± 1.3 | 0.010 |
|  | Cu-Cu | 4.4 ± 2.2 | 2.92 ± 0.017 | 8.6 ± 0.4 | 6.6 ± 1.0 |  |
| Cu_2_O | Cu-O | 1.8 ± 0.2 | 1.85 ± 0.010 | 2.8 ± 1.1 | 9.8 ± 1.2 | 0.010 |
|  | Cu-Cu | 18.6 ± 4.8 | 3.03 ± 0.012 | 25.3 ± 3.3 | 9.6 ± 0.8 |  |

N: coordination numbers; R: bond distance; σ^2^: Debye-Waller factors; ΔE: the inner potential correction. R factor: goodness of fit. * fitting with fixed parameter.


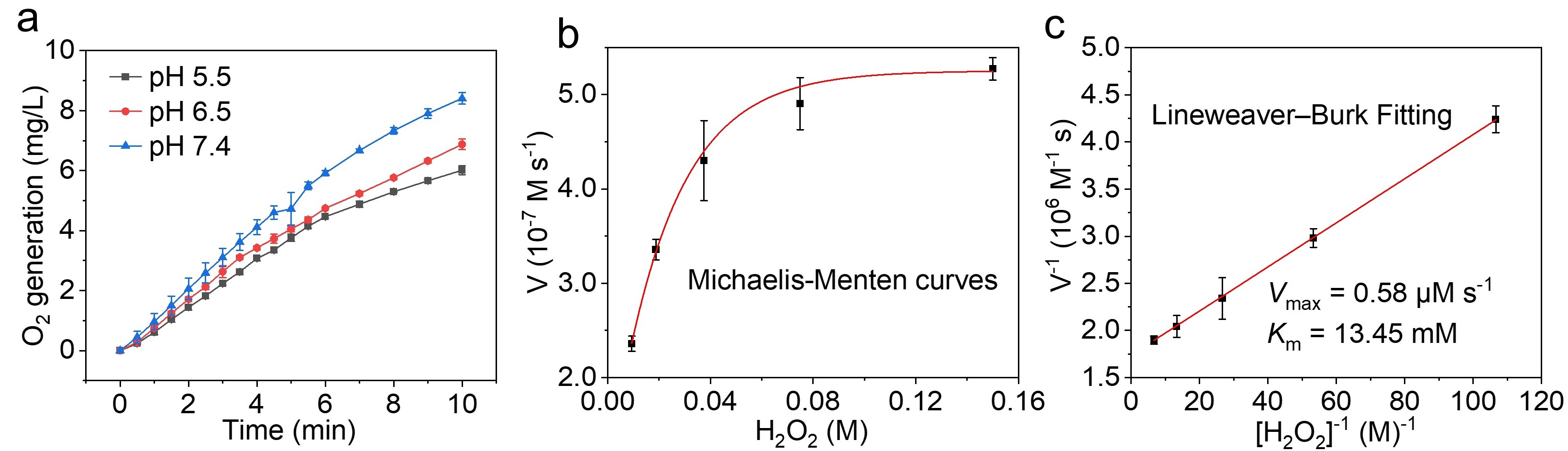


**Figure S8.** (a) Time-dependent O_2_ generation by CuZnONPs at pH = 5.5, 6.5, and 7.4. (b) Michaelis-Menten curves and (c) Lineweaver-Burk plotting of CAT-like activity of CuZnONPs. Data are presented as mean ± SD (n = 3).


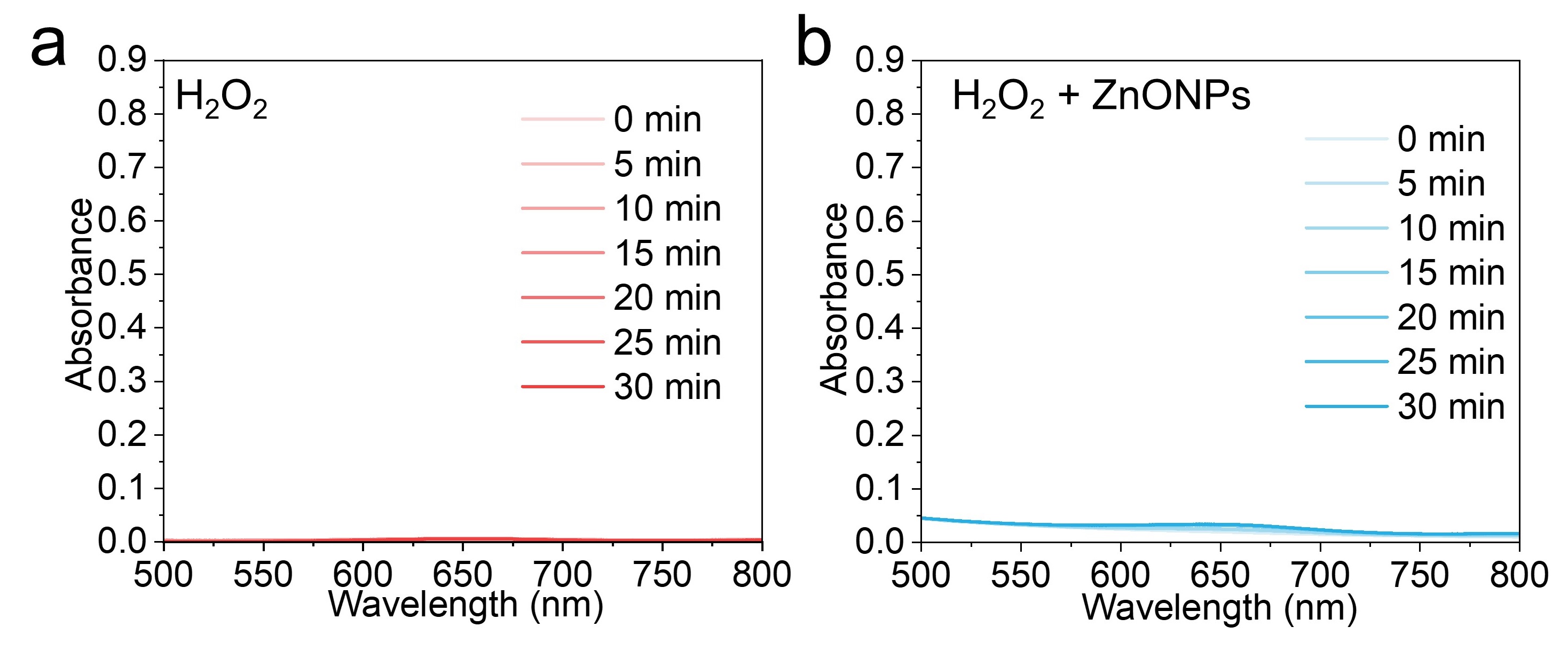


**Figure S9.** Time-dependent absorption of TMB incubated with (a) H_2_O_2_ and (b) H_2_O_2_ + ZnONPs for the determination of ·OH generation.


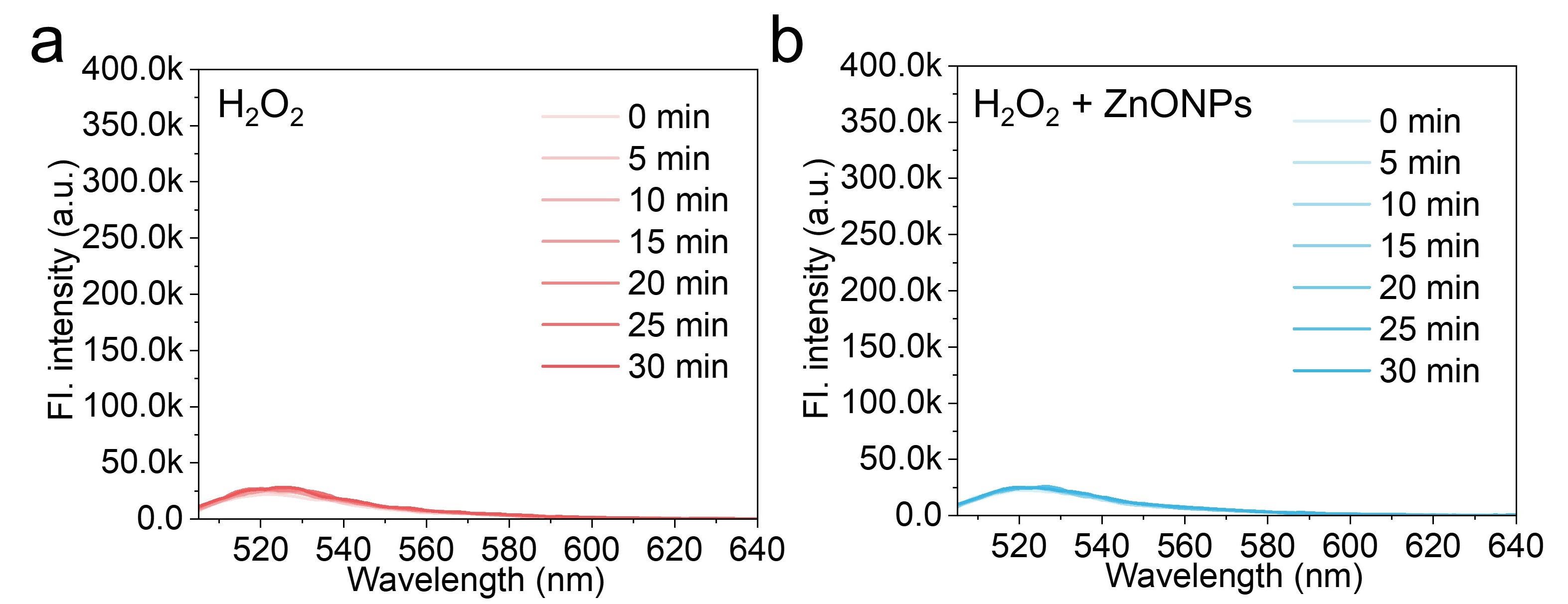


**Figure S10.** Time-dependent emission of DHR 123 incubated with (a) H_2_O_2_ and (b) H_2_O_2_ + ZnONPs for the determination of ·O_2_^-^ generation.


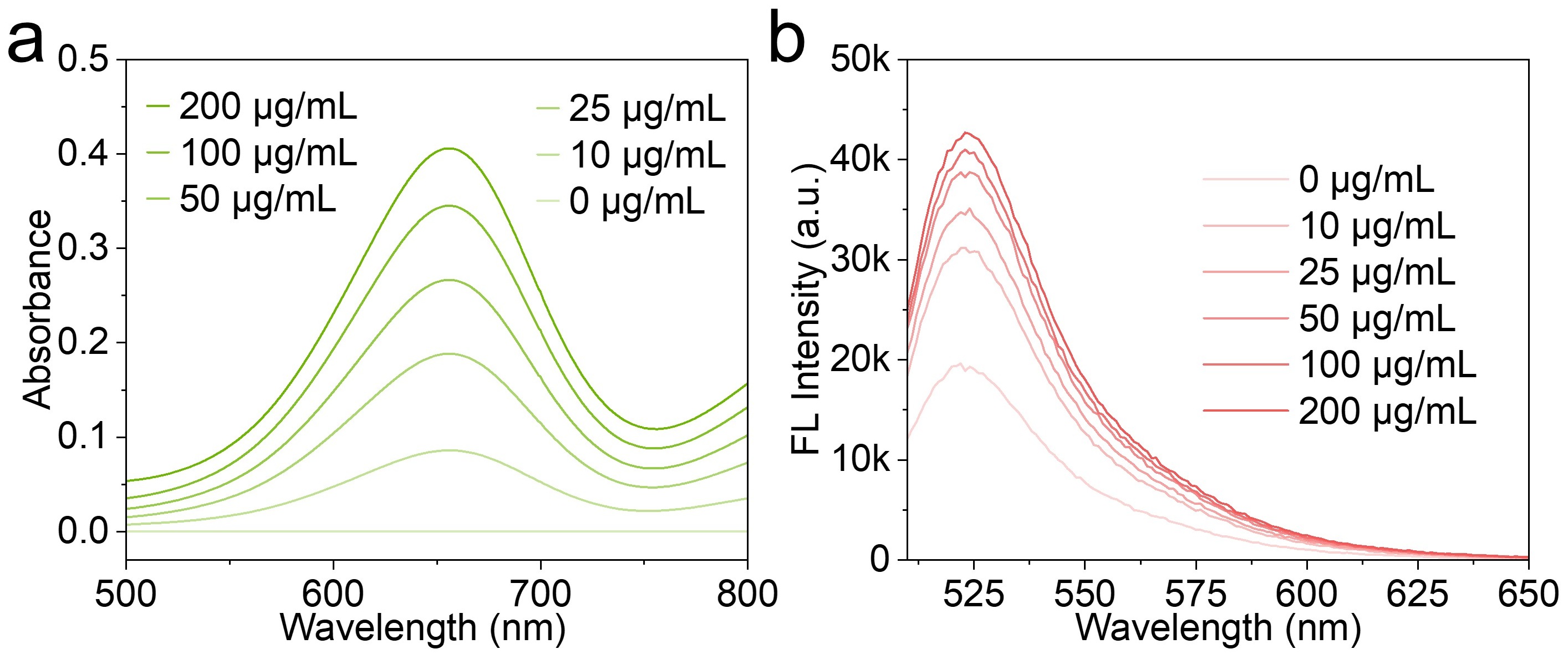


**Figure S11.** Concentration-dependent generation of (a) ·OH and (b) ·O_2_^-^ by CuZnONPs, as monitored by the spectral changes of TMB and DHR 123, respectively.


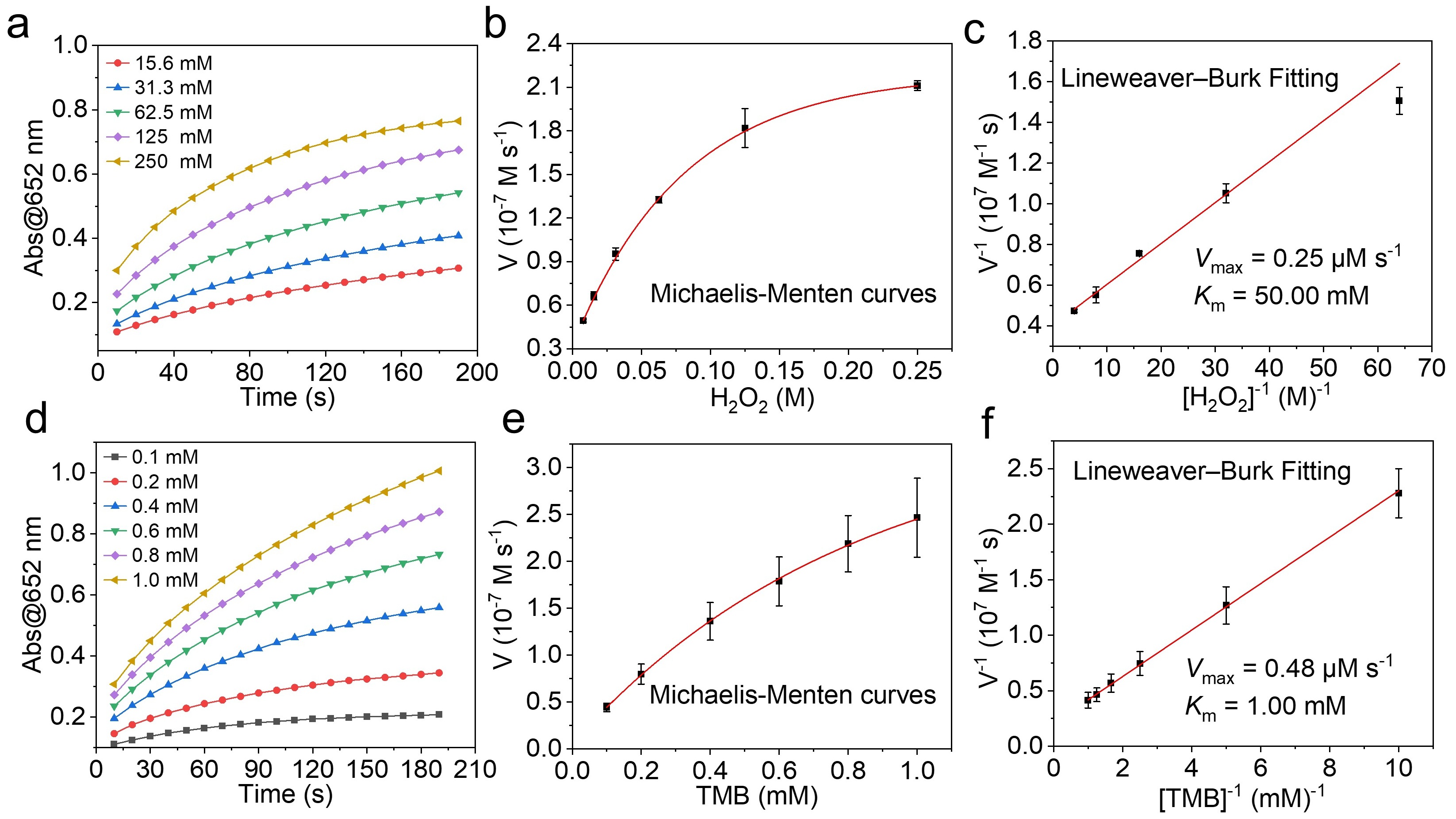


**Figure S12.** The absorbance change at 652 nm was measured to assess (a) POD-like and (d) OXD-like activities of CuZnONPs in the presence of H_2_O_2_. (b) Michaelis-Menten curves and (c) Lineweaver-Burk plotting of POD-like activity of CuZnONPs. (e) Michaelis-Menten curves and (f) Lineweaver-Burk plotting of OXD-like activity of CuZnONPs. Data are presented as mean ± SD (n = 3).


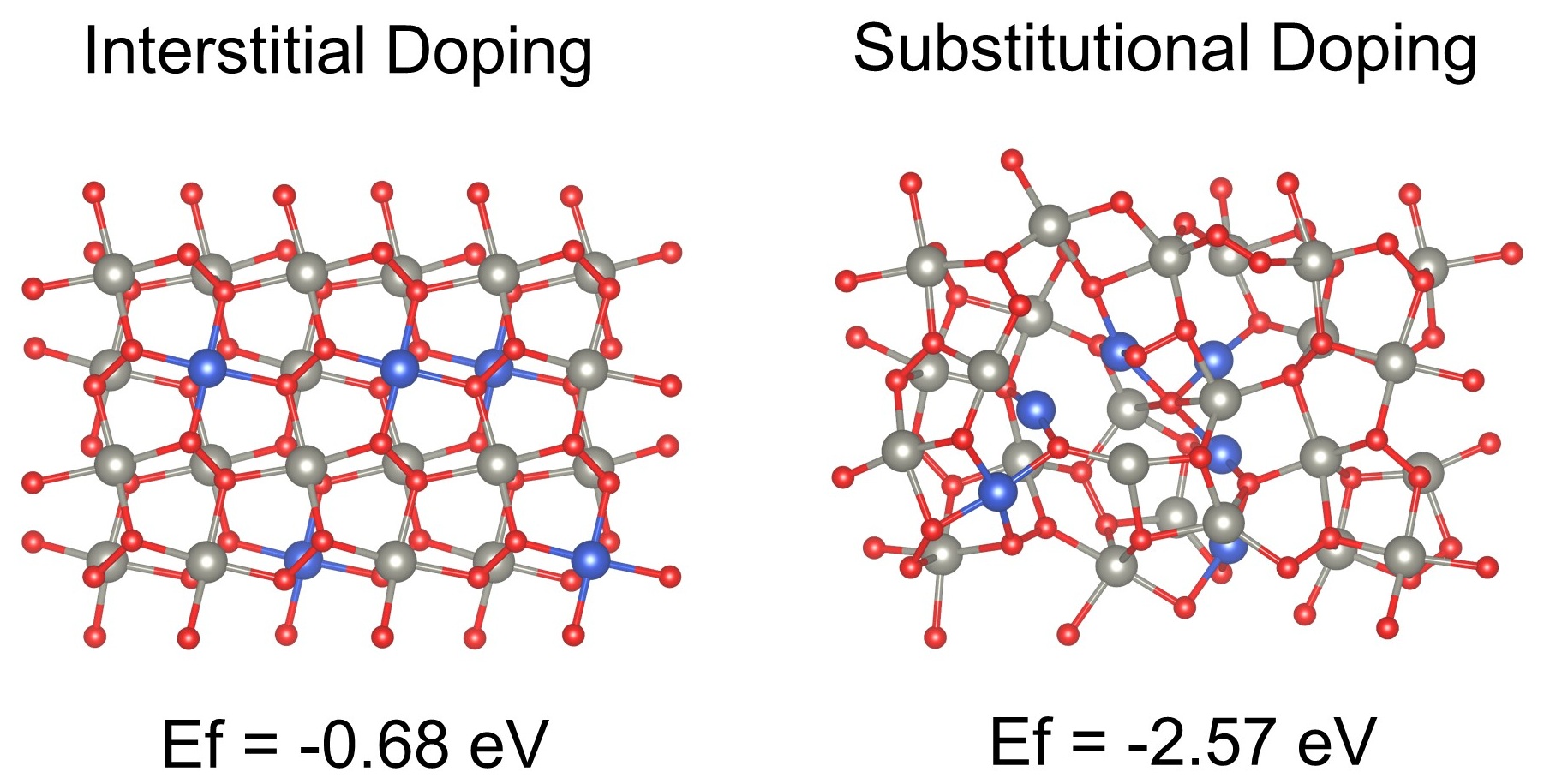


**Figure S13.** Calculated structure models of CuZnONPs.


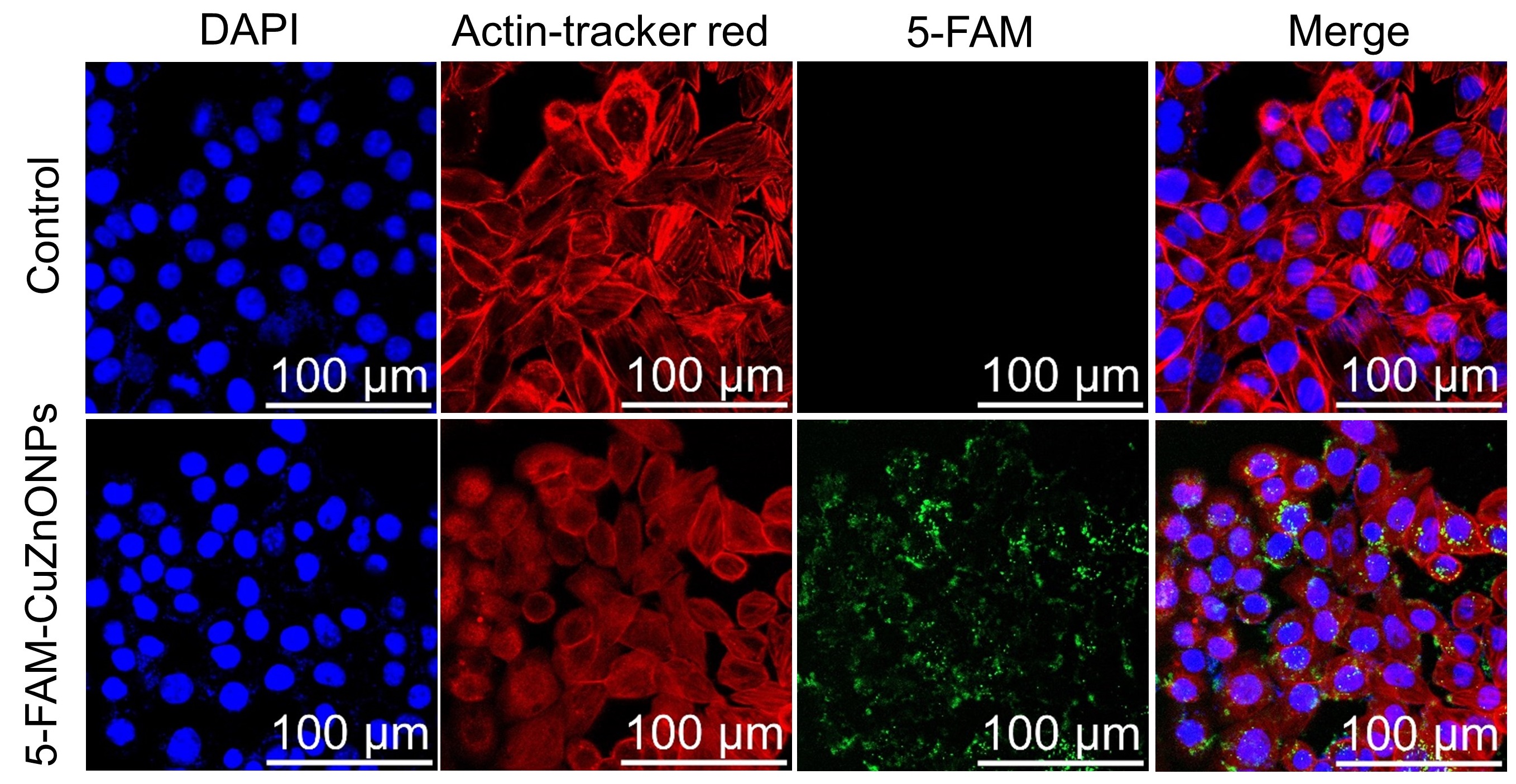


**Figure S14.** CLSM images of 4T1 cells after incubation with or without 5-FAM labeled CuZnONPs (5-FAM-CuZnONPs). Blue fluorescence: DAPI for nuclei; red fluorescence: Actin-Tracker for cytoskeleton; green fluorescence: 5-FAM for nanoparticles.


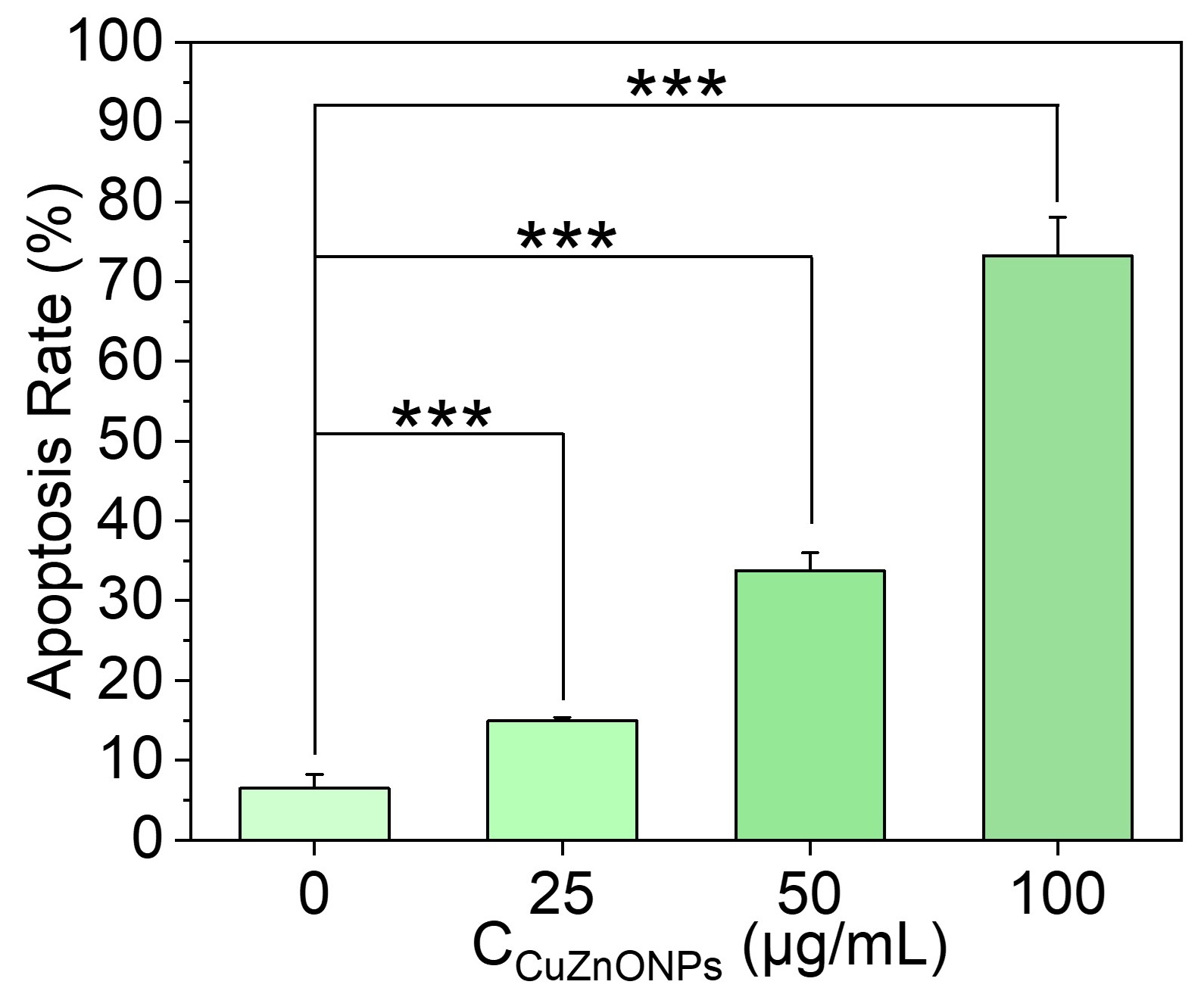


**Figure S15.** Apoptosis analysis of 4T1 cells determined by flow cytometry and the corresponding quantitative results after incubation with different concentrations of CuZnONPs. Data are presented as mean ± SD (n = 3), and statistical significance was assessed by a one-way ANOVA. ****P* < 0.001.


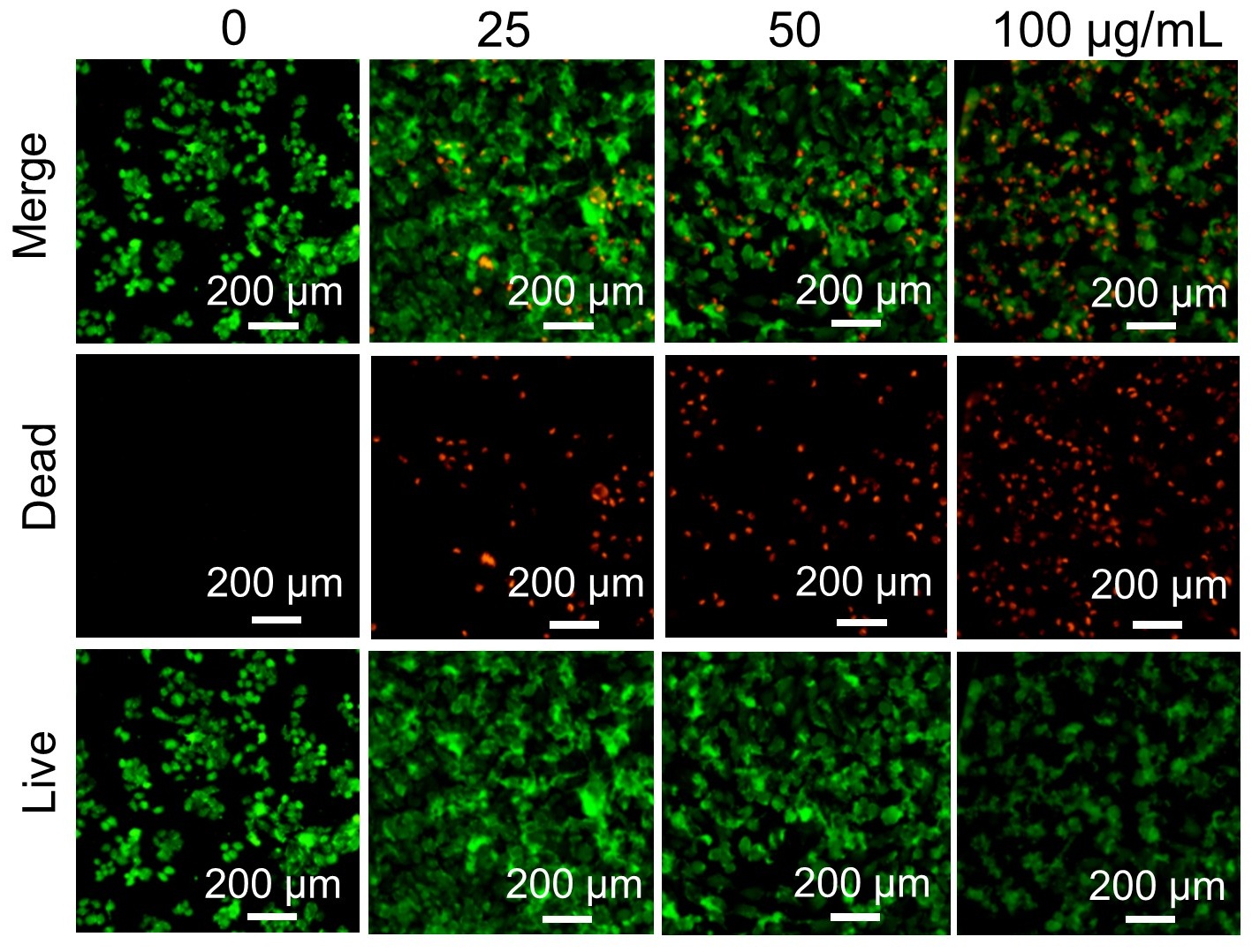


**Figure S16.** Live-dead staining of 4T1 cells after treatment with CuZnONPs. Green fluorescence: calcium-AM for living cells, red fluorescence: PI for dead cells.


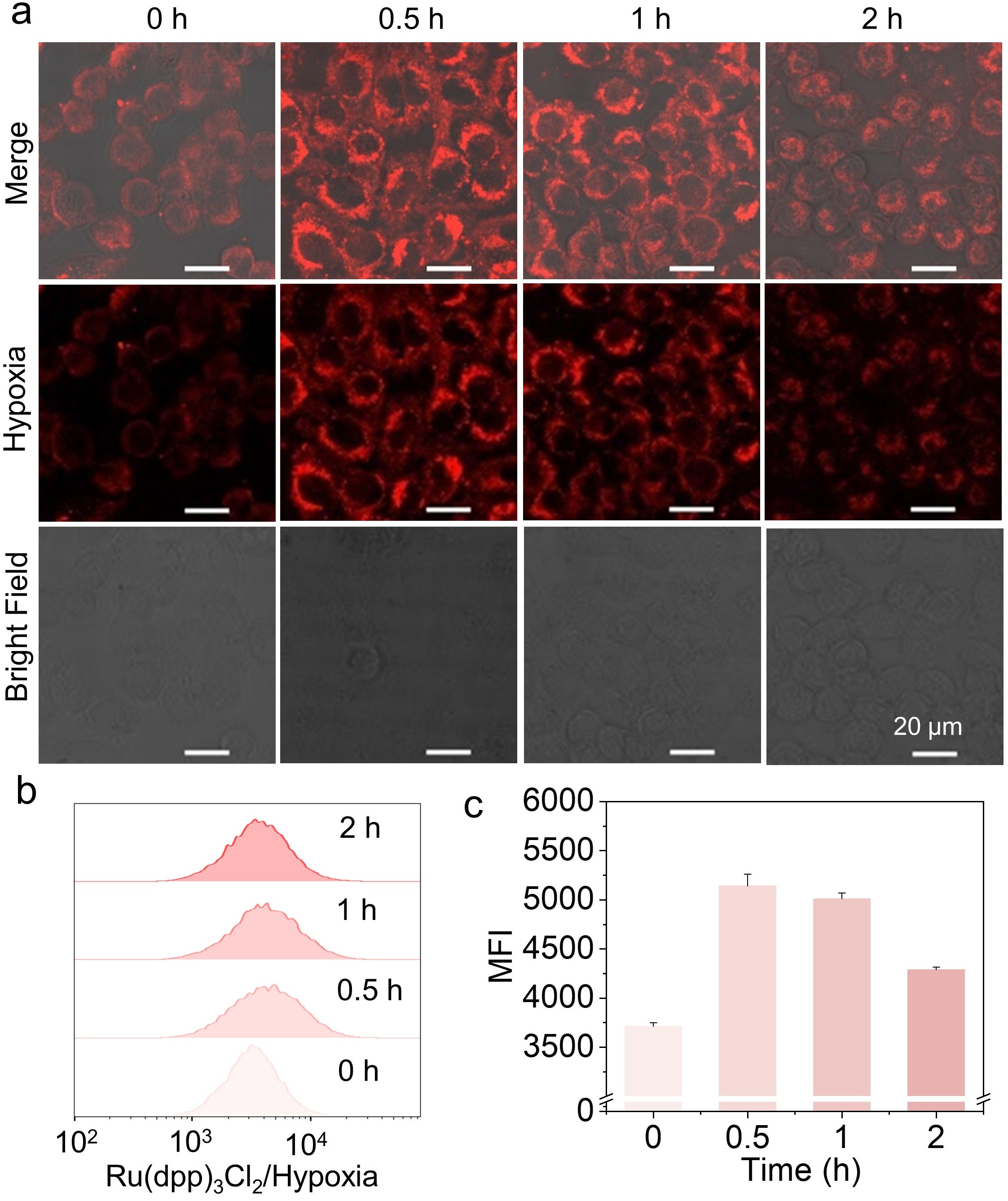


**Figure S17.** (a) Intracellular O_2_ detection using Ru(dpp)_3_Cl_2_ after CuZnONPs treatment with different incubation time. (b) Flow cytometric analysis and (c) statistical results of intracellular O_2_ in 4T1 cells after treatment with CuZnONPs.


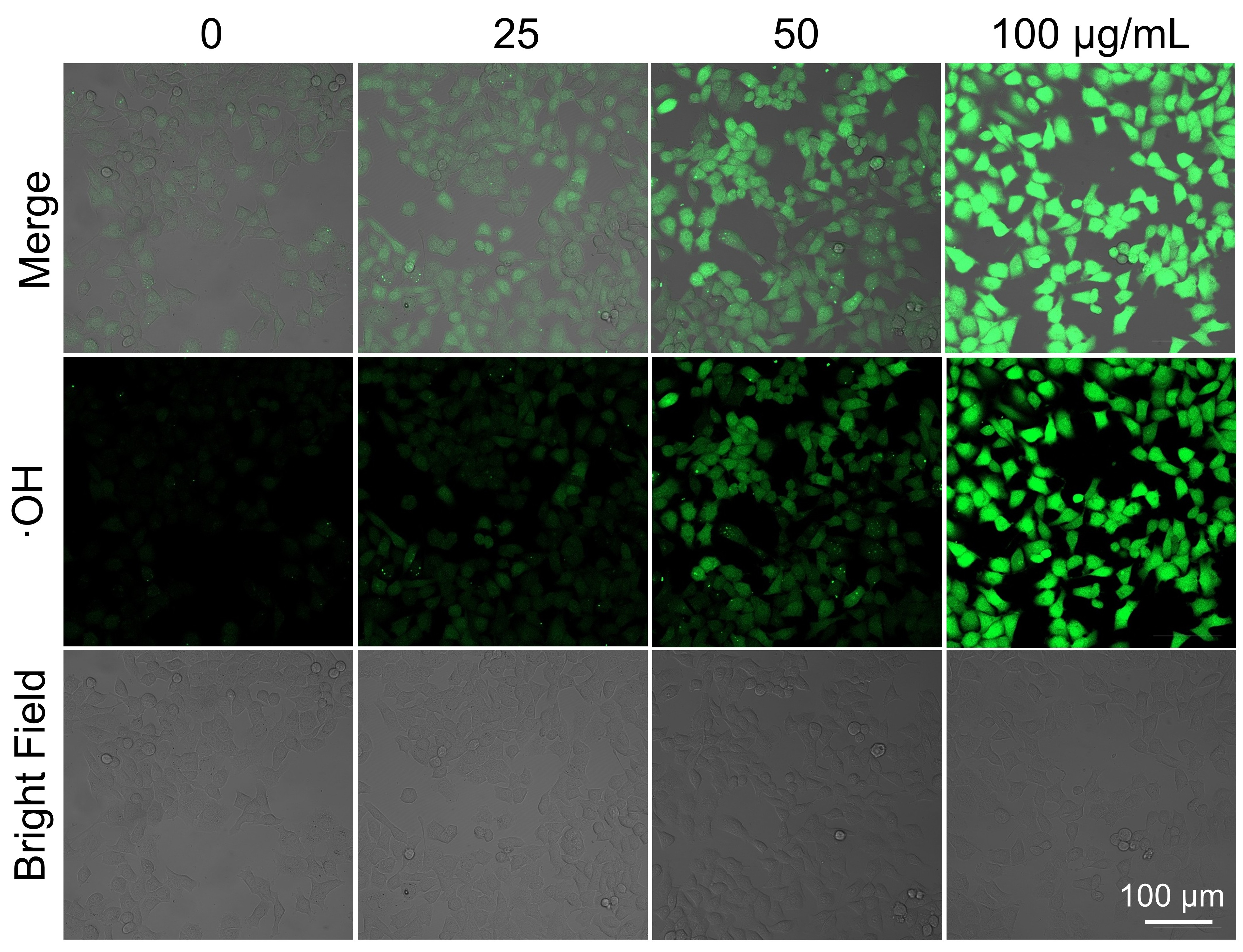


**Figure S18.** CLSM images of intracellular ·OH stained with HPF after treating with different concentrations of CuZnONPs.


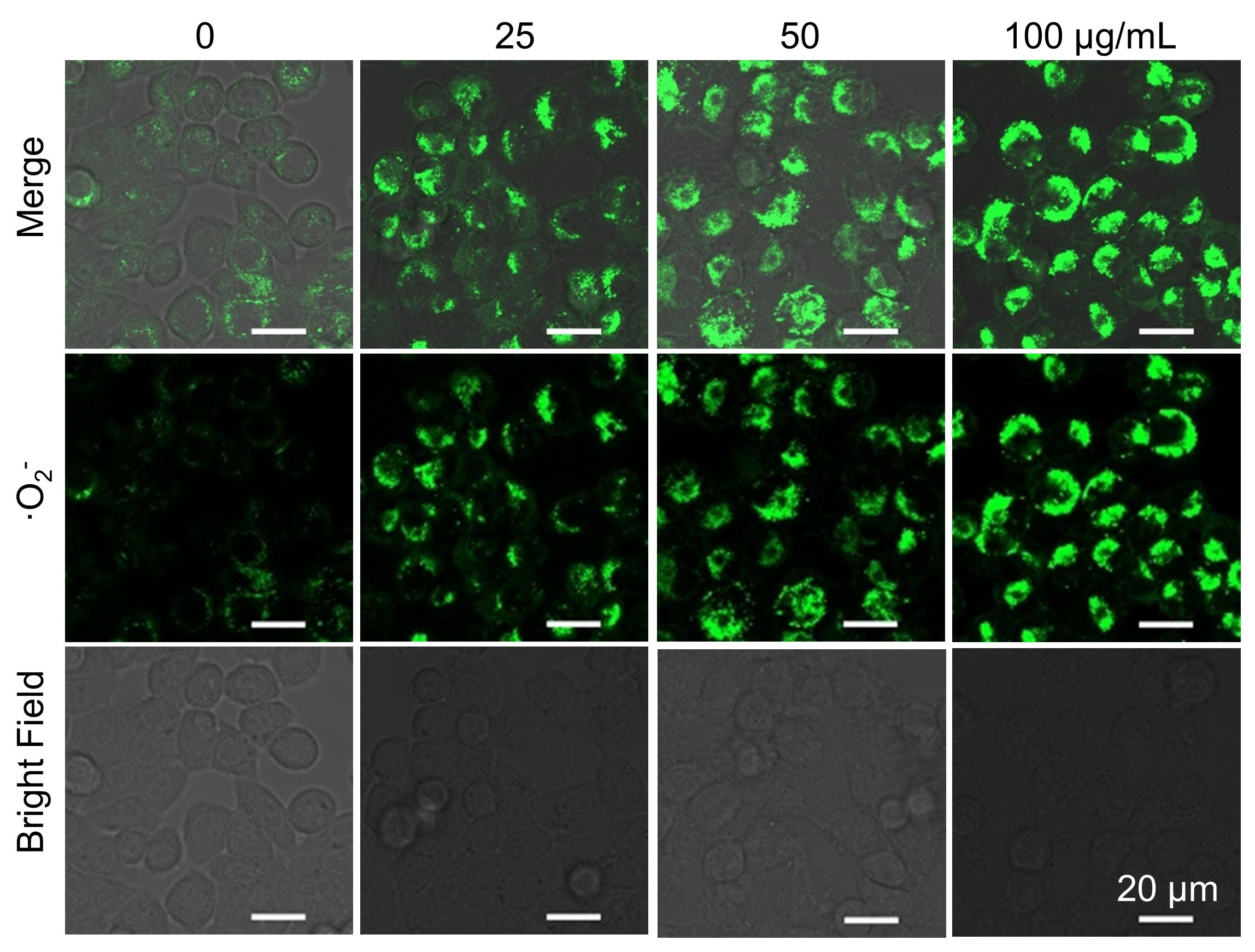


**Figure S19.** CLSM images of intracellular ·O_2_^-^ stained with DHR 123 after treating with different concentrations of CuZnONPs.


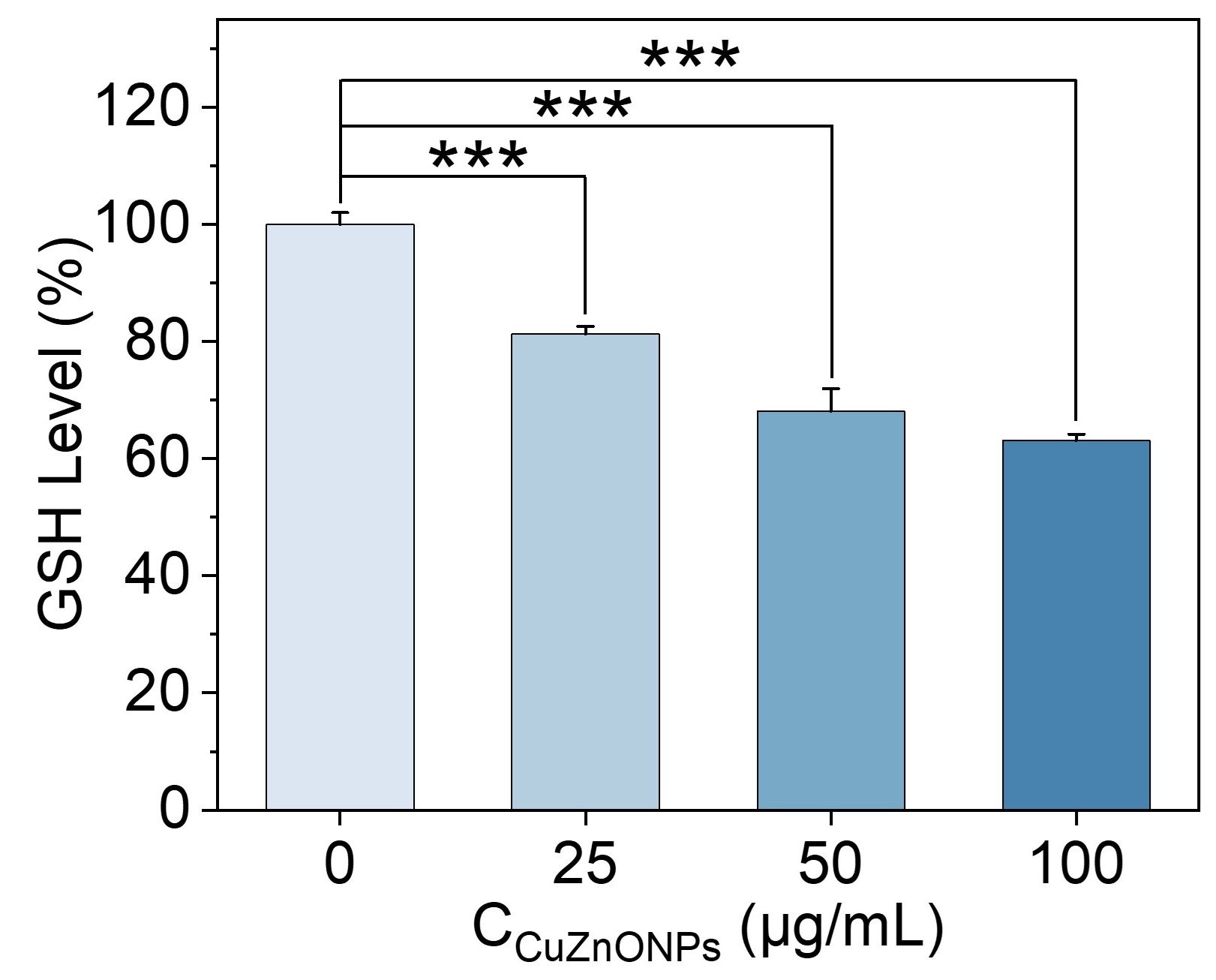


**Figure S20.** GSH levels in 4T1 cells measured by DTNB after treating with different concentrations of CuZnONPs. Data are presented as mean ± SD (n = 3), and statistical significance was assessed by a one-way ANOVA. ****P* < 0.001.


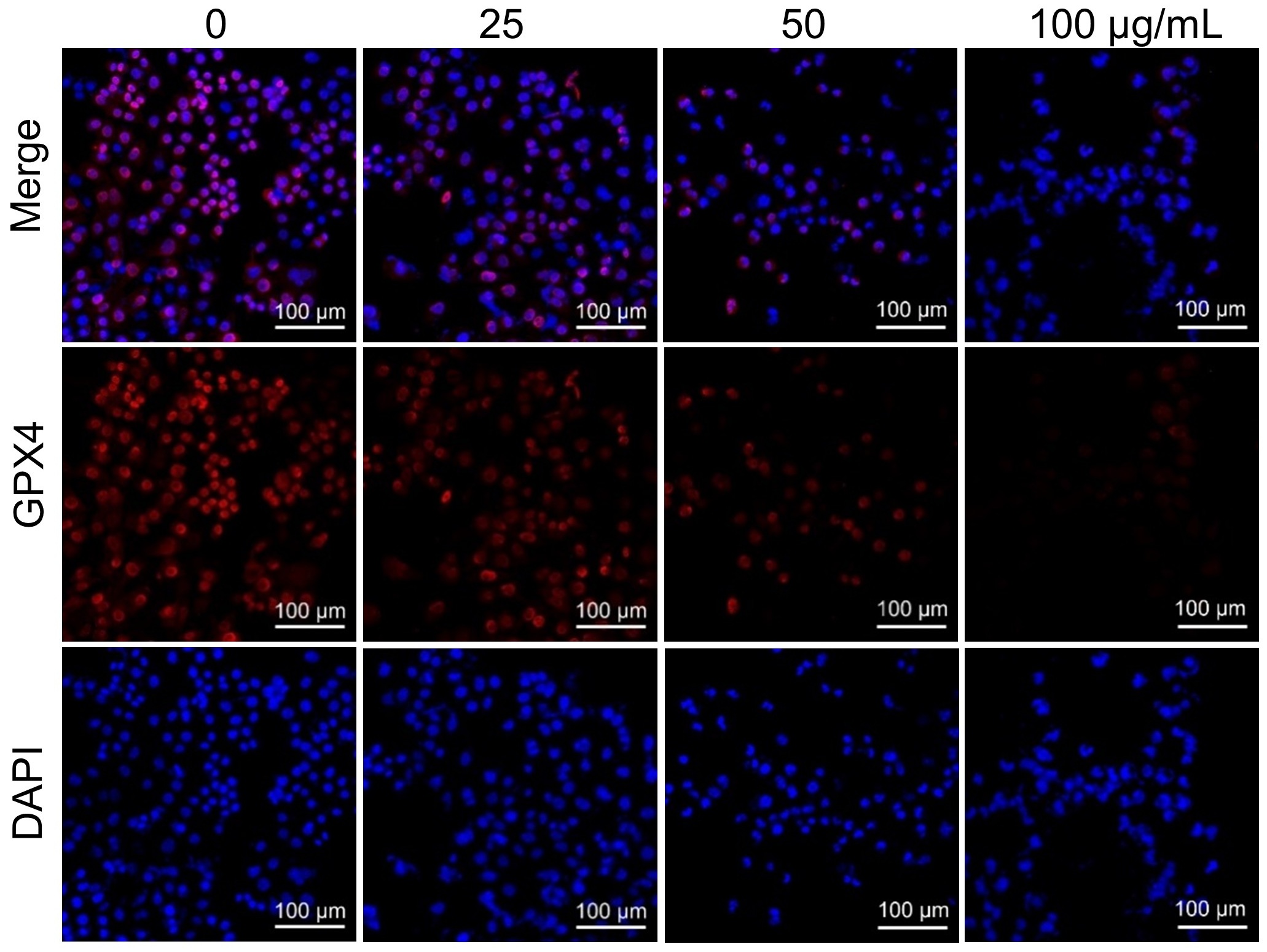


**Figure S21.** Immunofluorescence images of GPX4 in 4T1 cells after treatment with different concentrations of CuZnONPs. GPX4 was stained with anti-rabbit GPX4 (red) and Cy3-labeled goat anti-rabbit IgG, and the nucleus was stained with DAPI (blue), respectively.


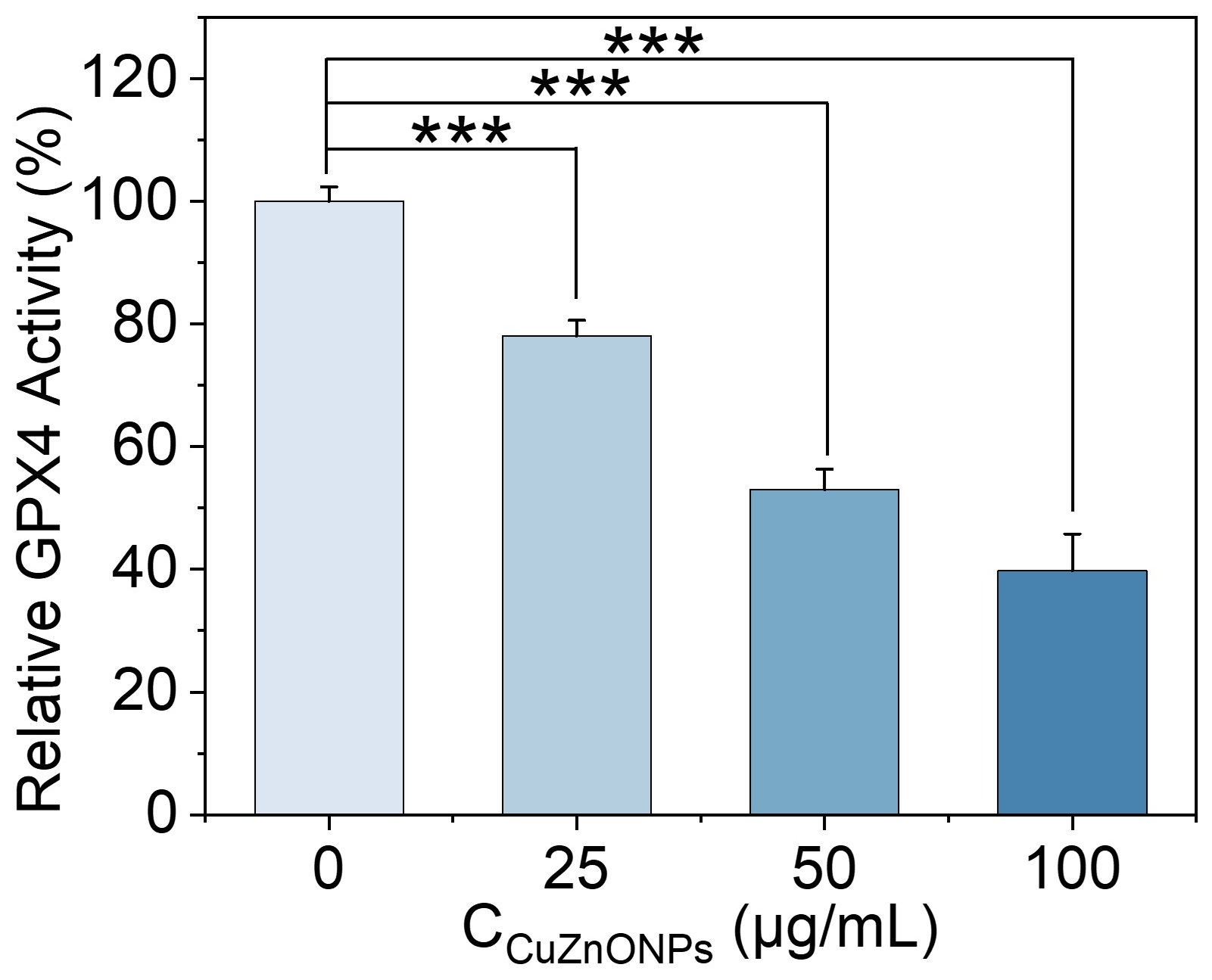


**Figure S22.** GPX4 activity in 4T1 cells treated with different concentrations of CuZnONPs, measured by a GPX4 assay kit. Data are presented as mean ± SD (n = 3), and statistical significance was assessed by a one-way ANOVA. ****P* < 0.001.


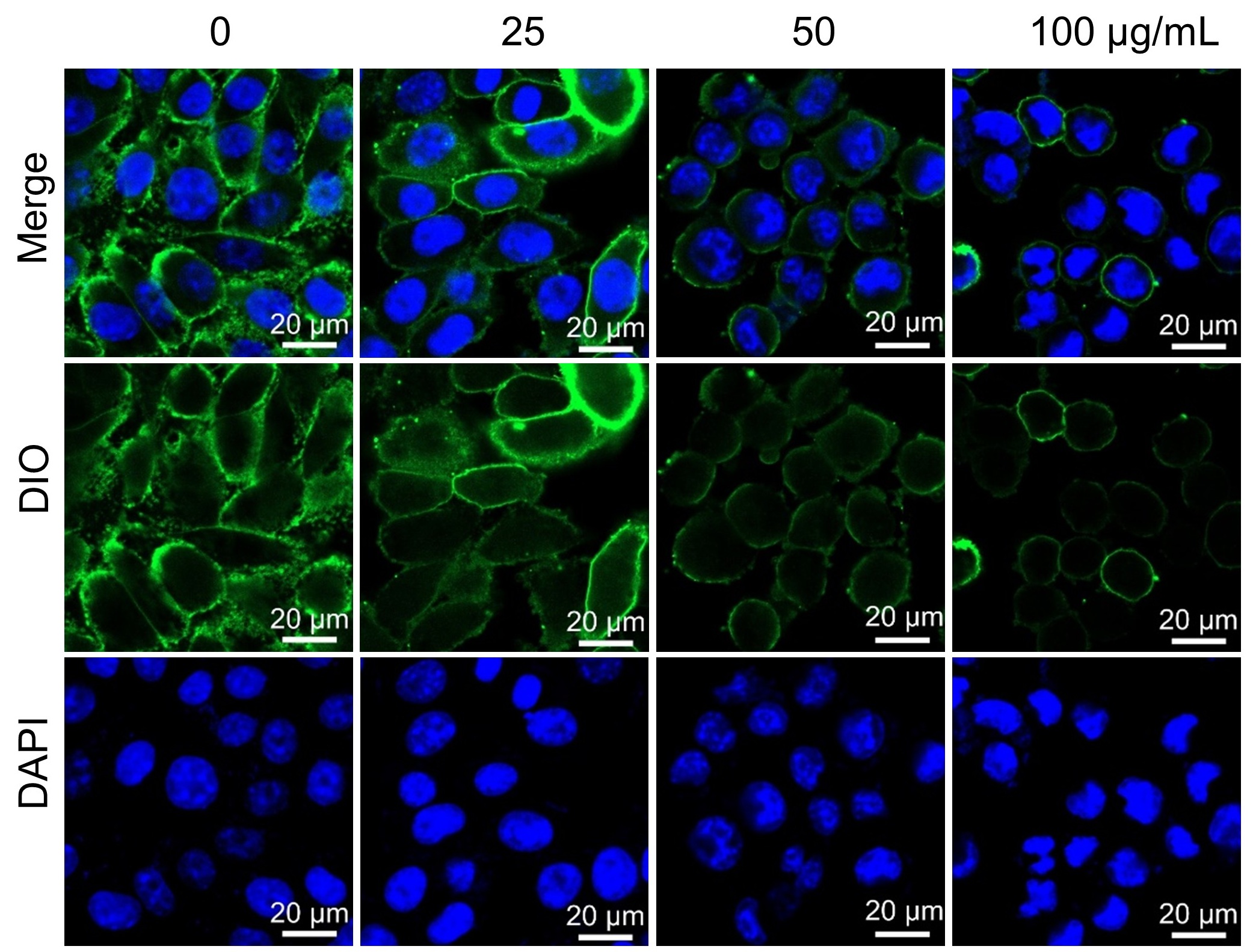


**Figure S23.** 4T1 cells stained with DIO (cell membrane: green) and DAPI (nucleus: blue) after treating with different concentrations of CuZnONPs.


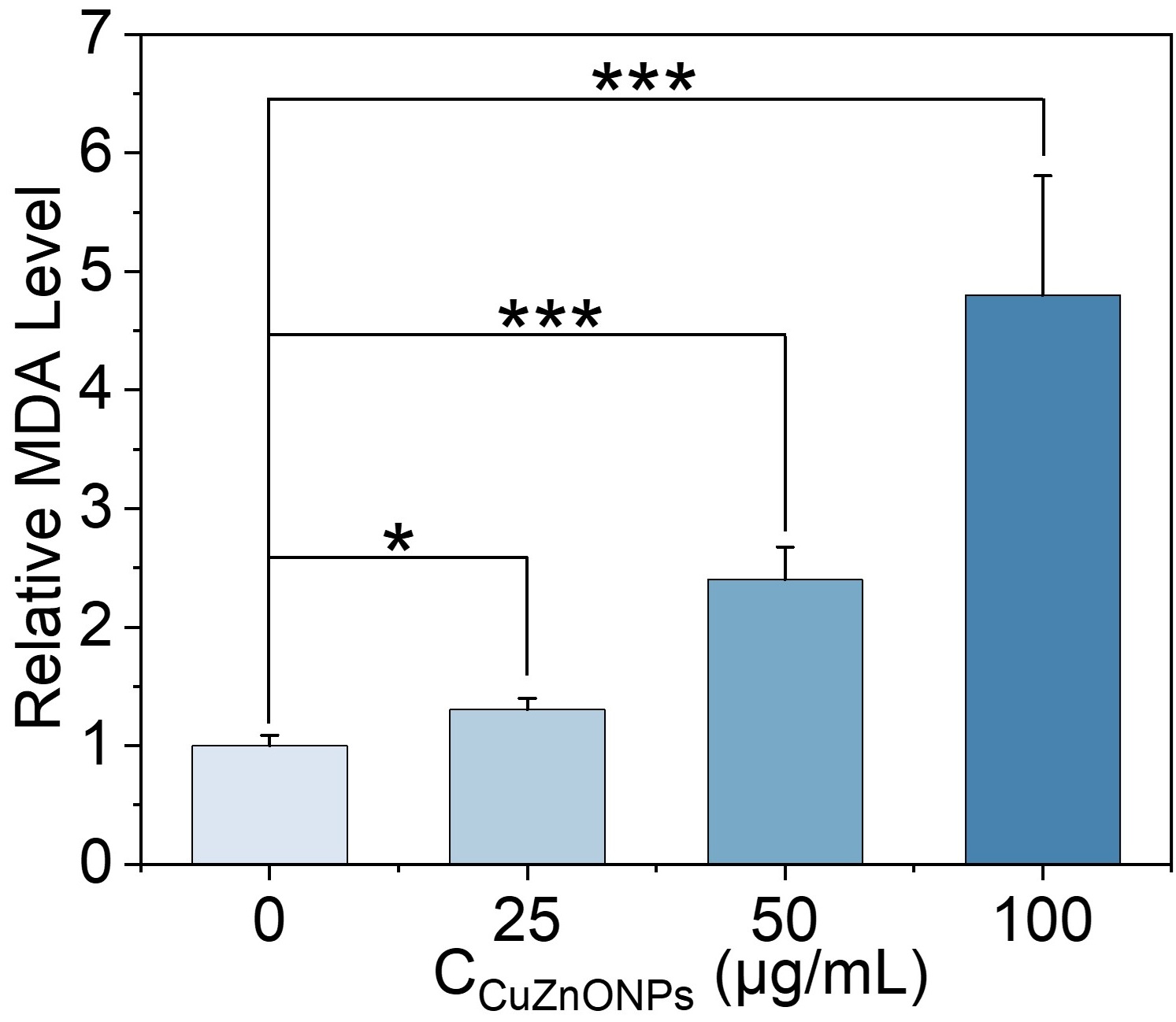


**Figure S24.** Intracellular malondialdehyde (MDA) levels measured by an MDA assay kit. Data are presented as mean ± SD (n = 3), and statistical significance was assessed by a one-way ANOVA. **P* < 0.05, ***P* < 0.01, ****P* < 0.001.


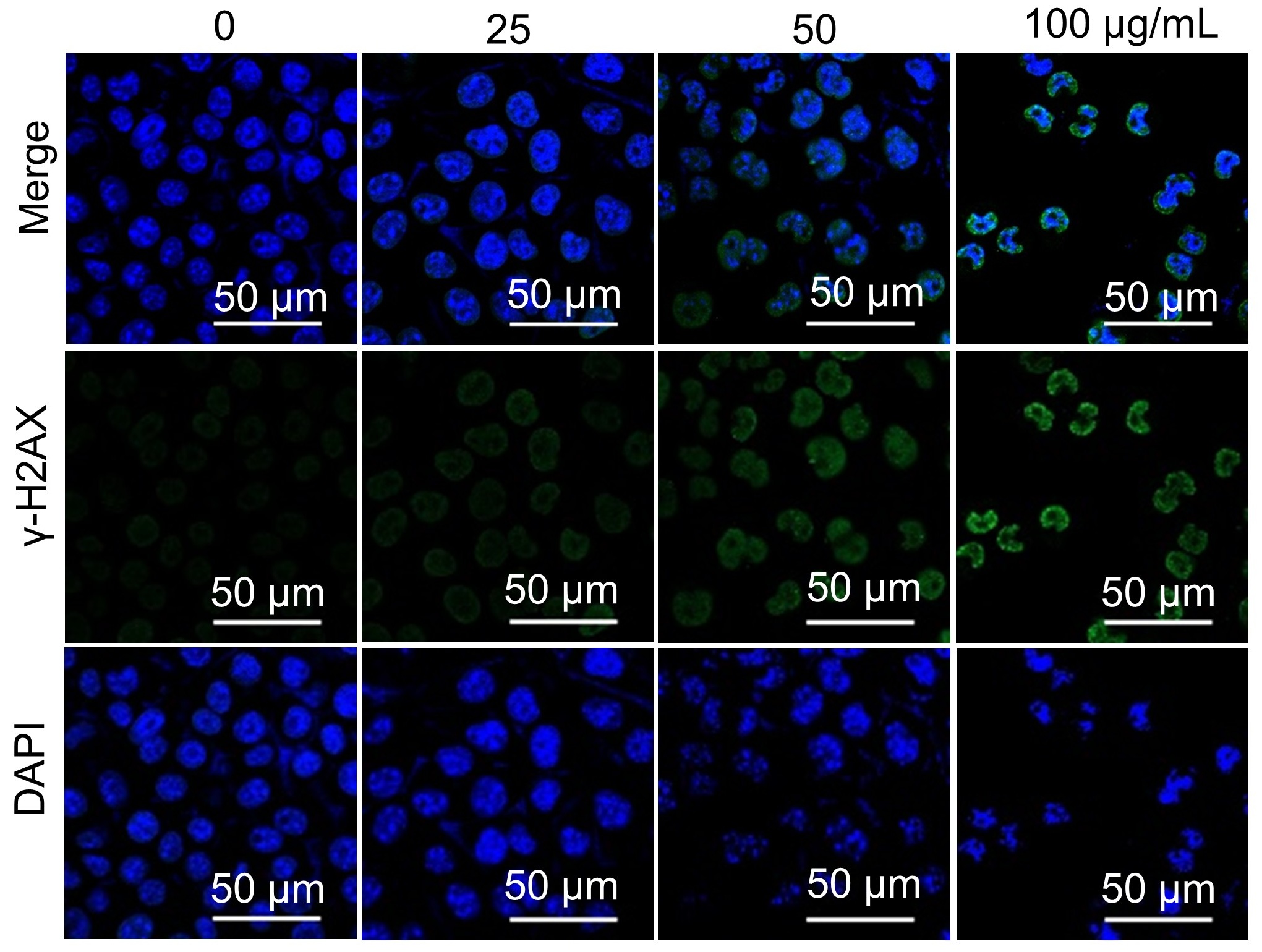


**Figure S25.** Immunofluorescence images of 4T1 cells stained with γ-H2AX (damaged DNA: green) and DAPI (nucleus: blue) after treatment with different concentrations of CuZnONPs.


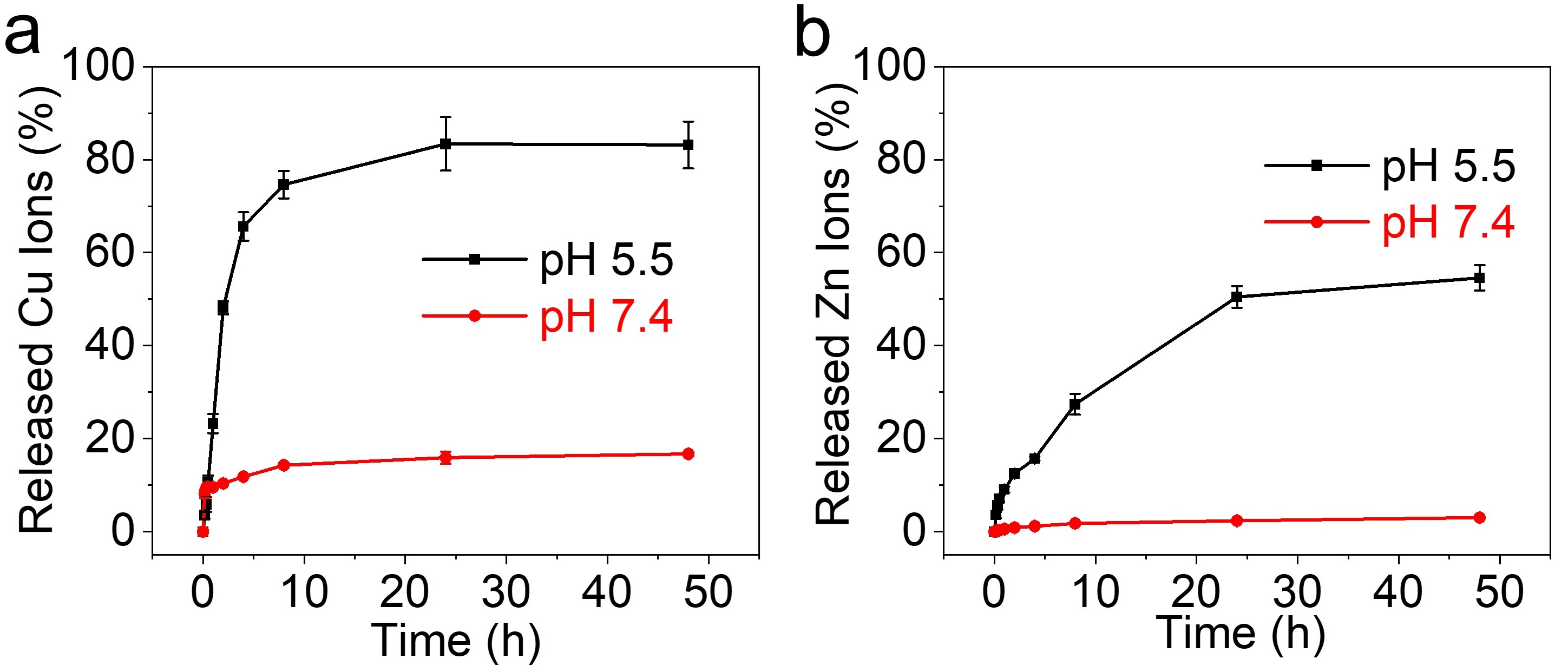


**Figure S26.** Release profiles of (a) Cu^2+^ and (b) Zn^2+^ from CuZnONPs at different pH values (5.5 and 7.4). Data are presented as mean ± SD (n = 3).


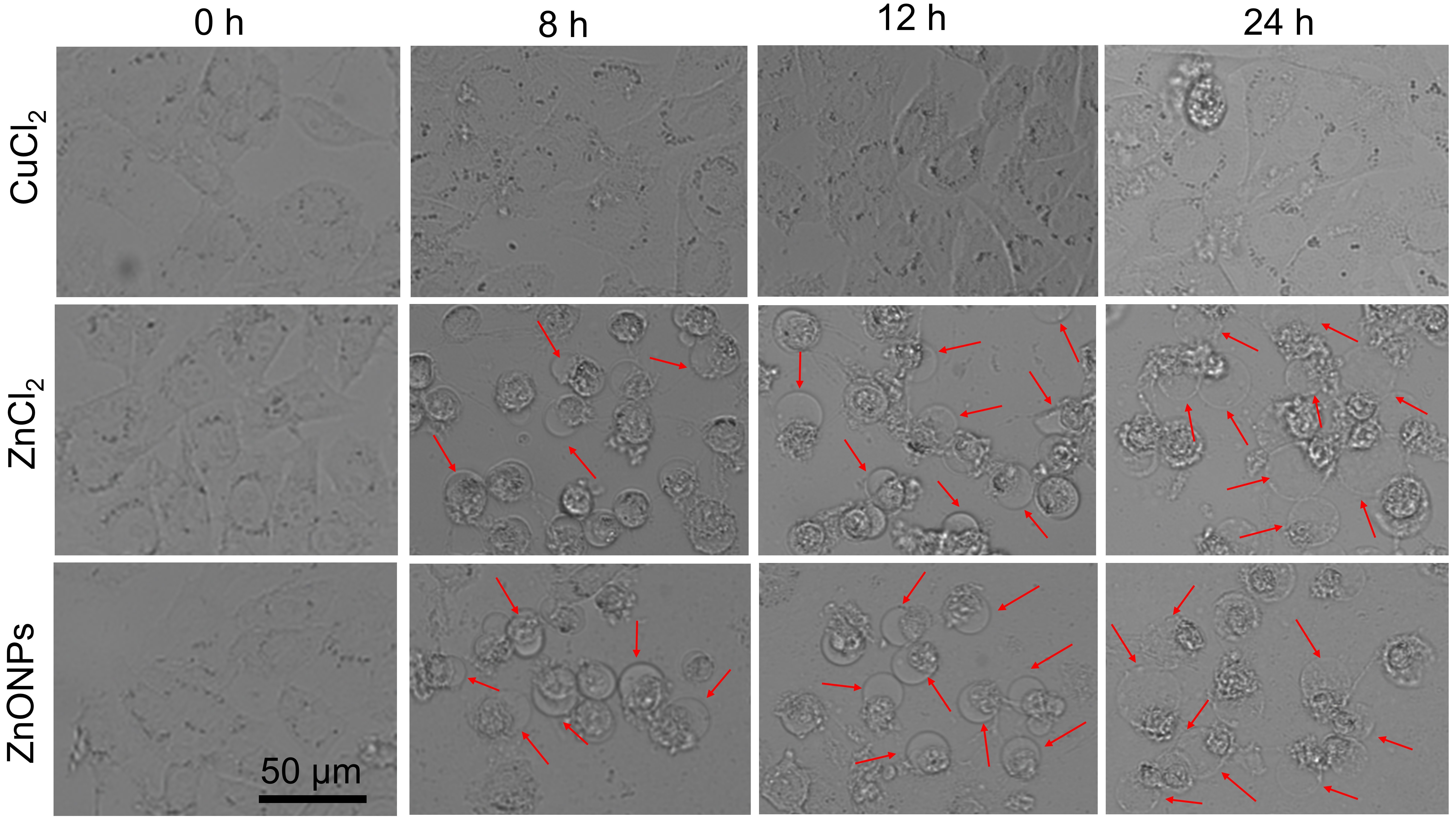


**Figure S27.** Morphology of 4T1 cells after treatment with CuCl_2_, ZnCl_2_ or ZnONPs for different time durations.


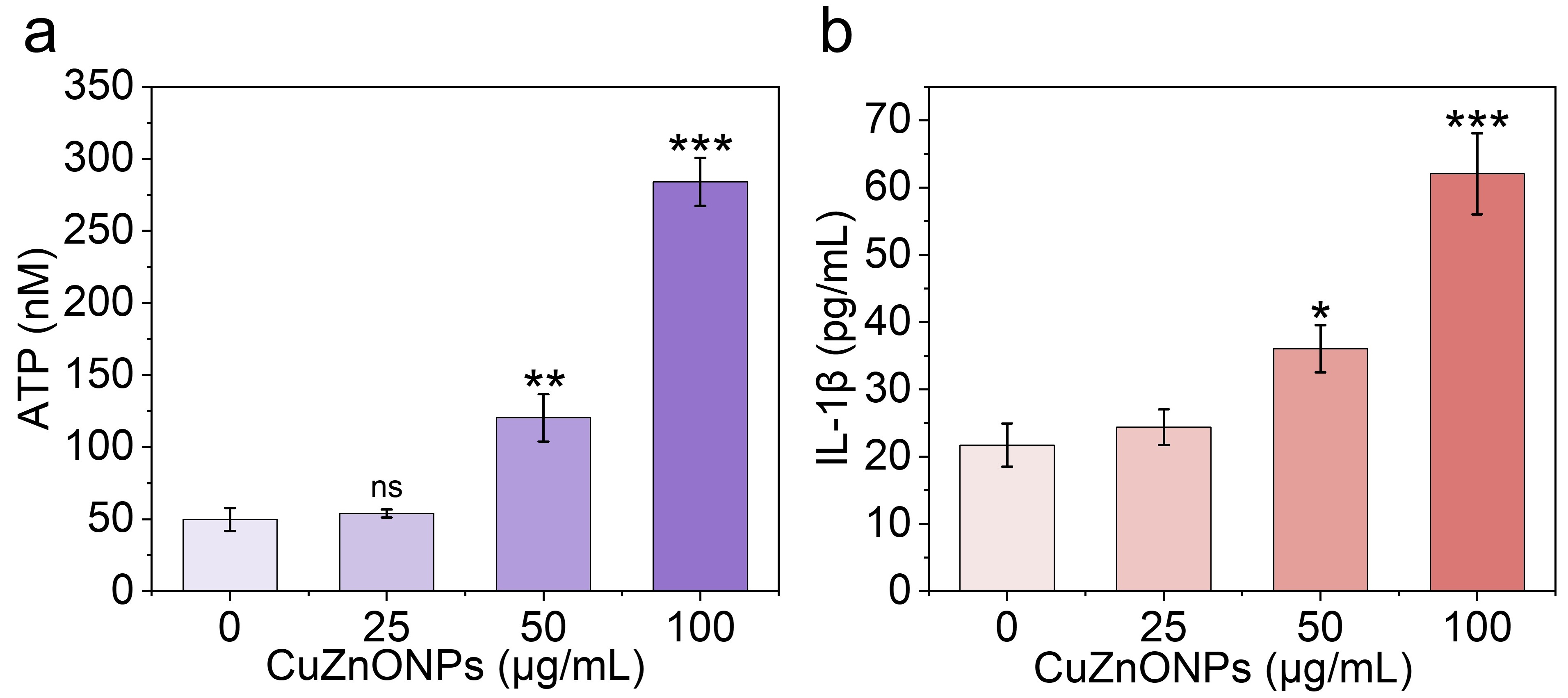


**Figure S28.** Concentration of (a) ATP and (b) IL-1β in the culture medium after treatment with CuZnONPs, measured by ELISA. Data are presented as mean ± SD (n = 3), and statistical significance was assessed by a one-way ANOVA. **P* < 0.05, ***P* < 0.01, ****P* < 0.001.


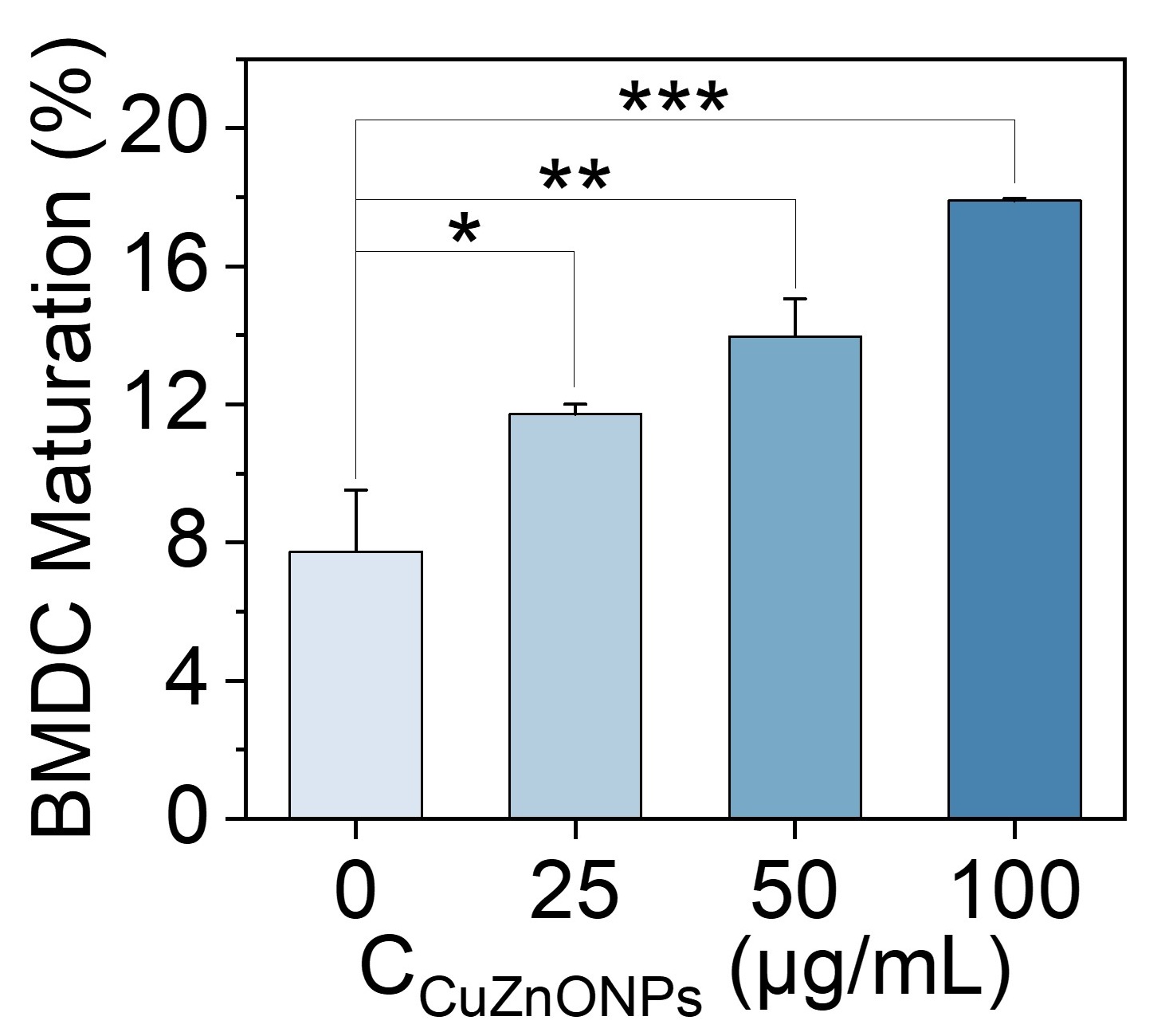


**Figure S29.** Flow cytometric quantification of BMDCs. Data are presented as mean ± SD (n = 3), and statistical significance was assessed by a one-way ANOVA. **P* < 0.05, ***P* < 0.01, ****P* < 0.001.


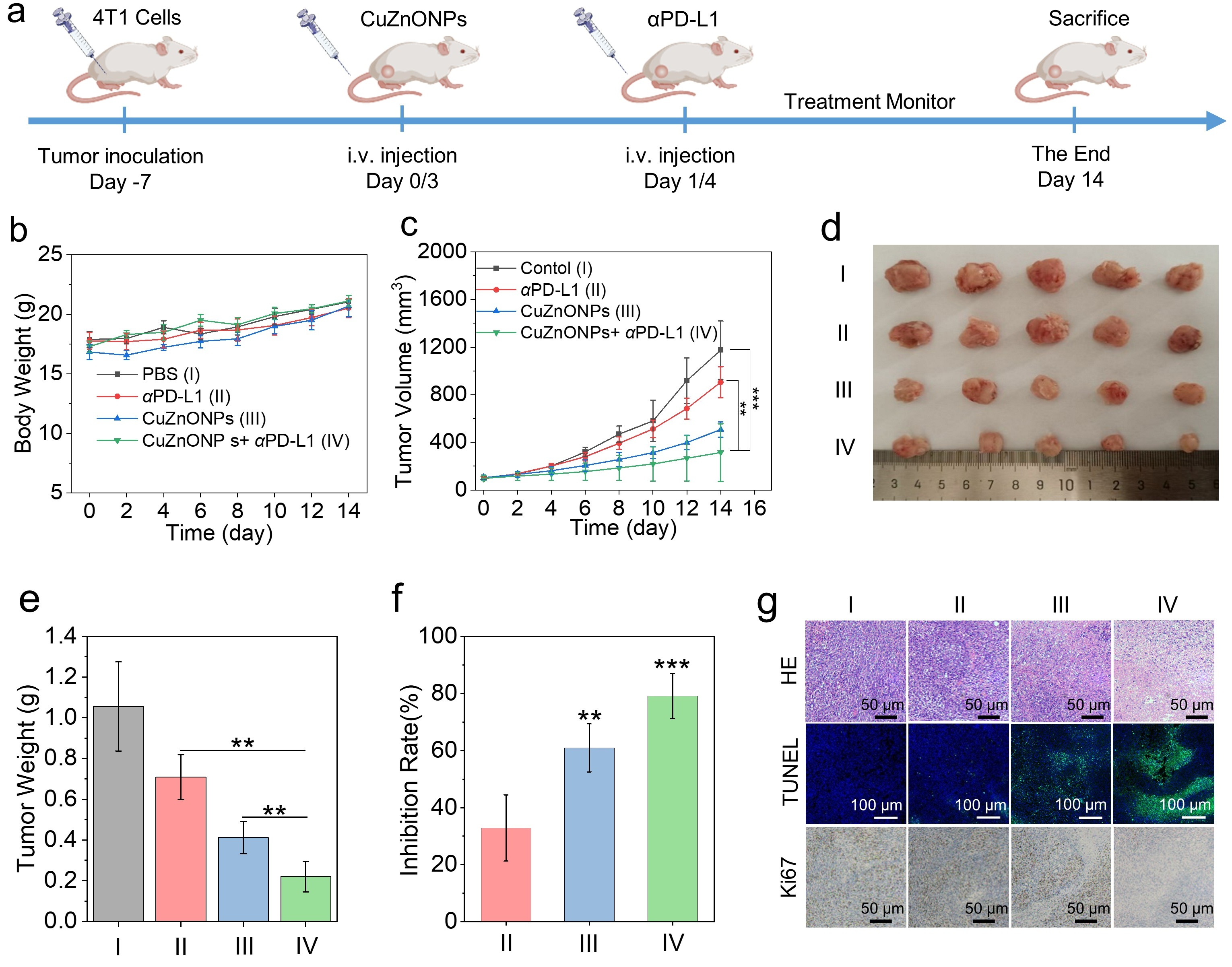


**Figure S30.** (a) Schematic illustration showing the experimental procedure of the anti-tumor effect of CuZnONPs. (b) Body weight and (c) Tumor volume of mice after treatment with (I) PBS, (II) *α*PD-L1, (III) CuZnONPs, or (IV) *α*PD-L1 + CuZnONPs. (d) Photos of tumors after treatment (day 14). (e) Tumor weight and (f) Inhibition rate on day 14. (g) H&E, TUNEL, and Ki67 staining images of tumor slides. Data are presented as mean ± SD (n = 5), and statistical significance was assessed by a one-way ANOVA. ***P* < 0.01, and ****P* < 0.001.


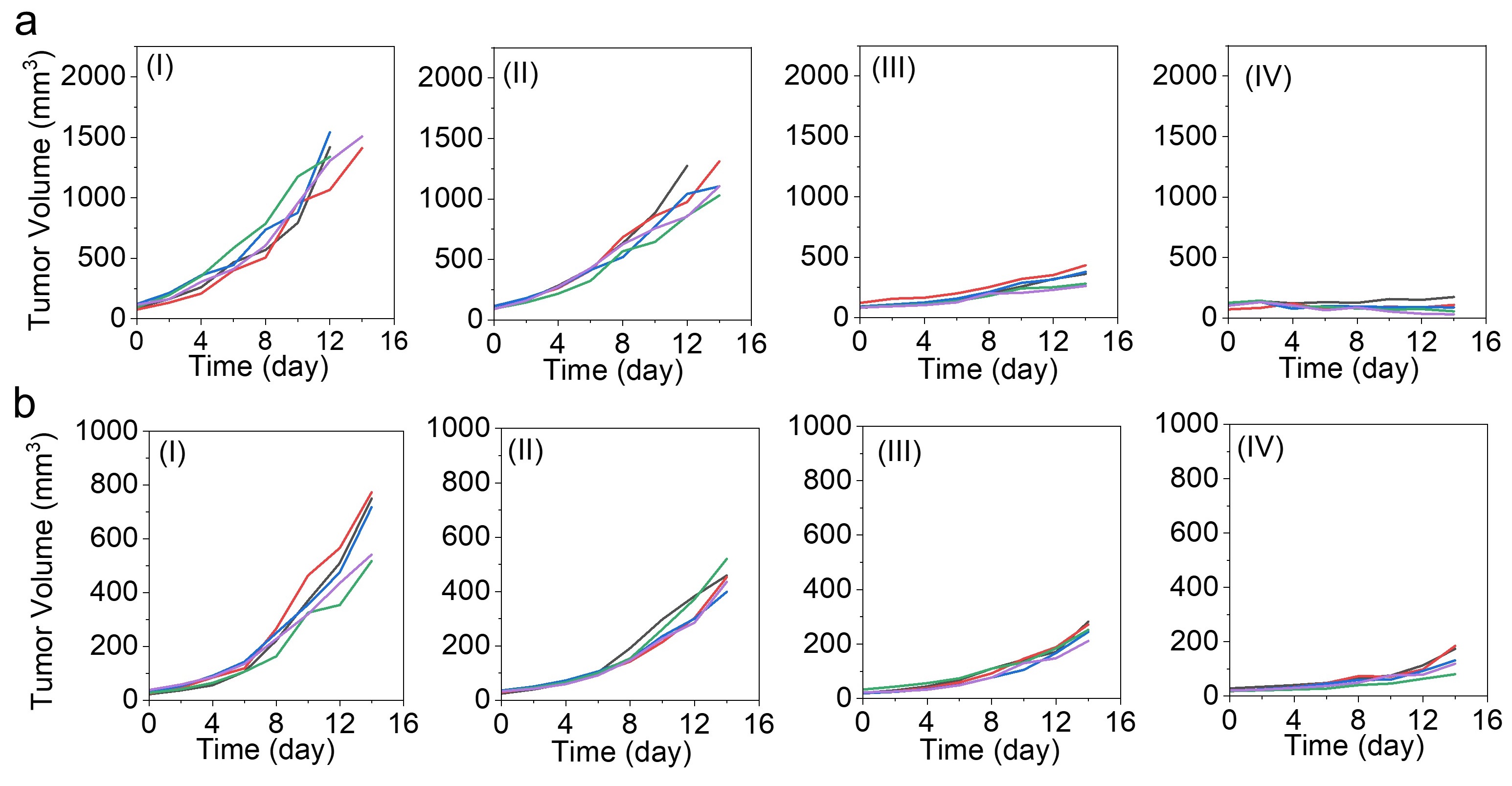


**Figure S31.** Volume curves of (a) primary tumor and (b) distant tumor: (I) PBS, (II) *α*PD-L1, (III) CuZnONPs and (IV) *α*PD-L1 + CuZnONPs.


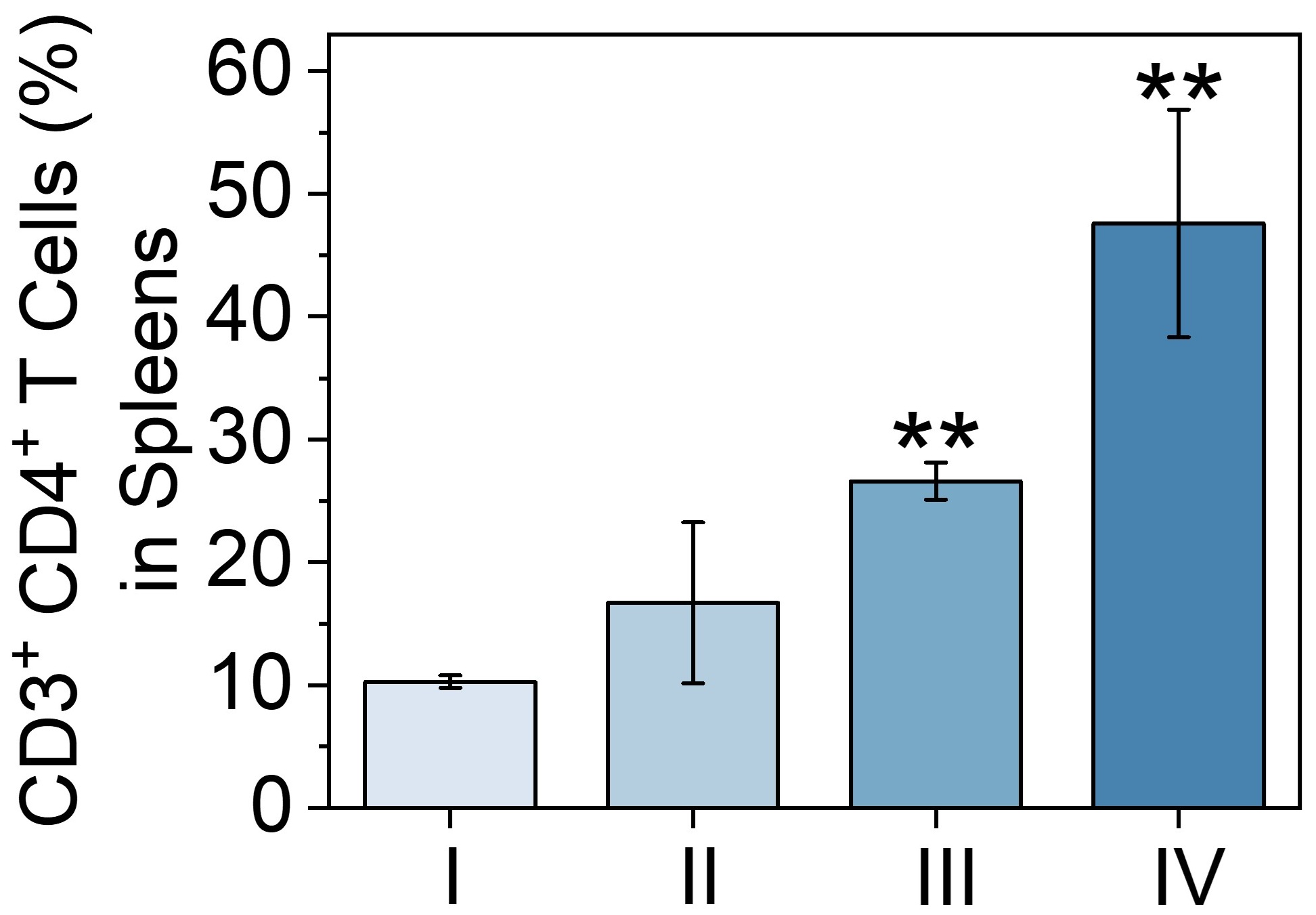


**Figure S32.** Flow cytometric quantification of CD4^+^ T cells in spleens after different treatments: (I) PBS, (II) *α*PD-L1, (III) CuZnONPs and (IV) *α*PD-L1 + CuZnONPs. Data are presented as mean ± SD (n = 3), and statistical significance was assessed by a one-way ANOVA. **P* < 0.05, ***P* < 0.01, ****P* < 0.001.


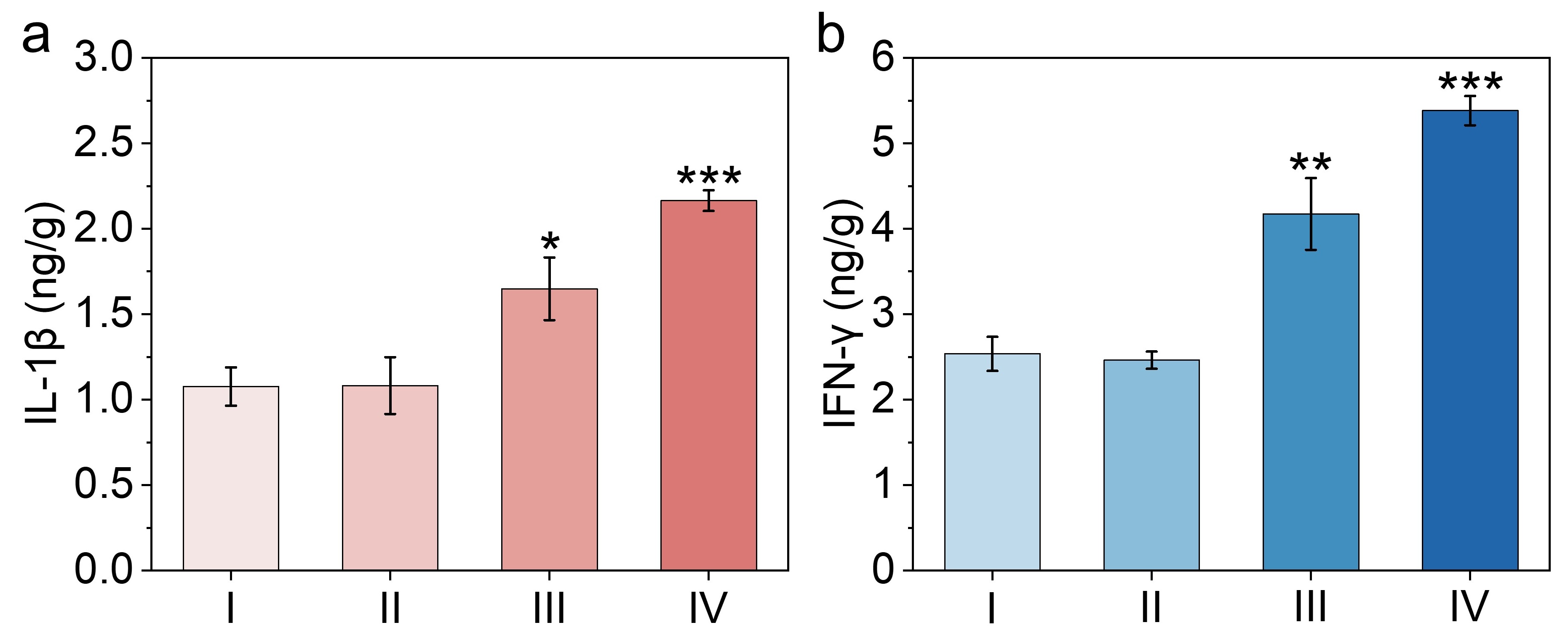


**Figure S33.** The levels of (a) IL-1β and (b) IFN-γ in the primary tumors receiving (I) PBS, (II) αPD-L1, (III) CuZnONPs, and (IV) αPD-L1 + CuZnONPs, detected by ELISA. Data are presented as mean ± SD (n = 3), and statistical significance was assessed by a one-way ANOVA. **P* < 0.05, ***P* < 0.01, and ****P* < 0.001.


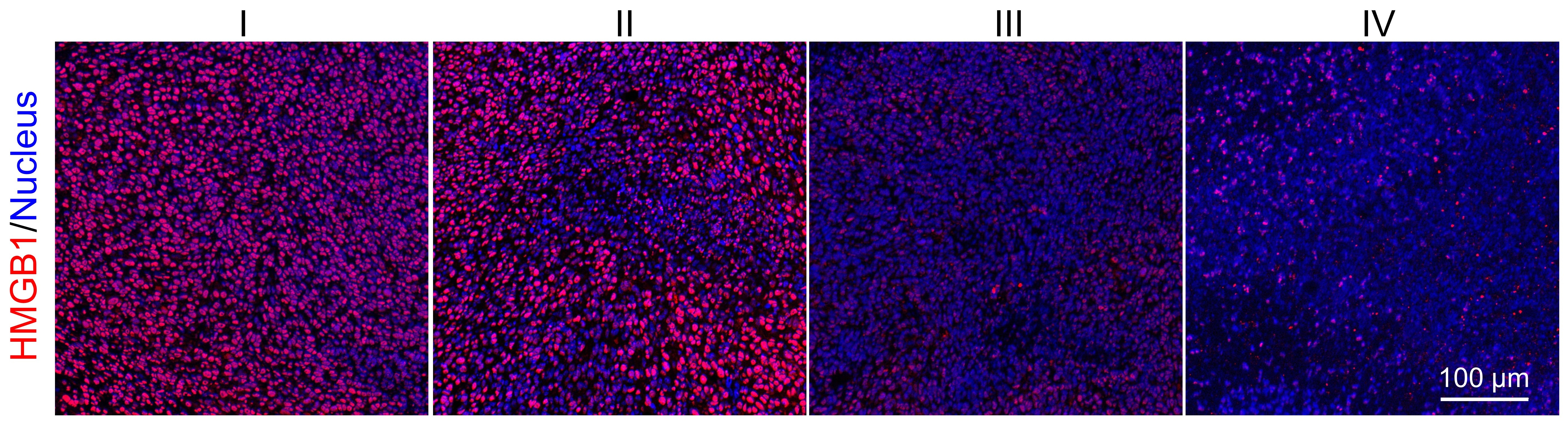


**Figure S34.** Representative immunofluorescence images showing HMGB1 expression in tumor tissues from tumor-bearing mice treated with (I) PBS, (II) αPD-L1, (III) CuZnONPs, and (IV) αPD-L1 + CuZnONPs.


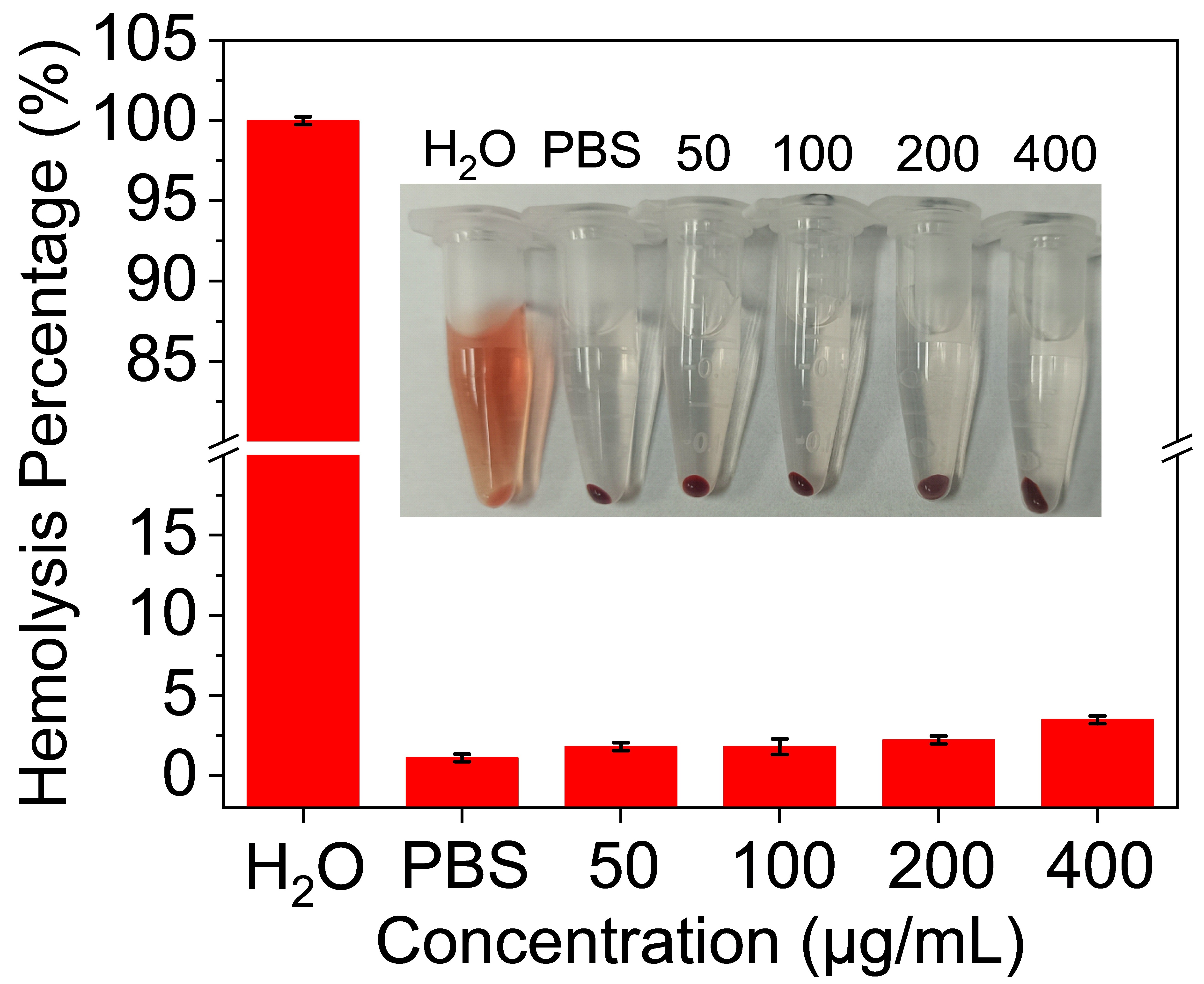


**Figure S35.** Quantitative analysis of hemolysis percentage of H_2_O, PBS, and CuZnONPs (50, 100, 200, or 400 μg/mL). Representative photograph of hemolysis is shown above the corresponding bars. Data are presented as mean ± SD (n = 3).


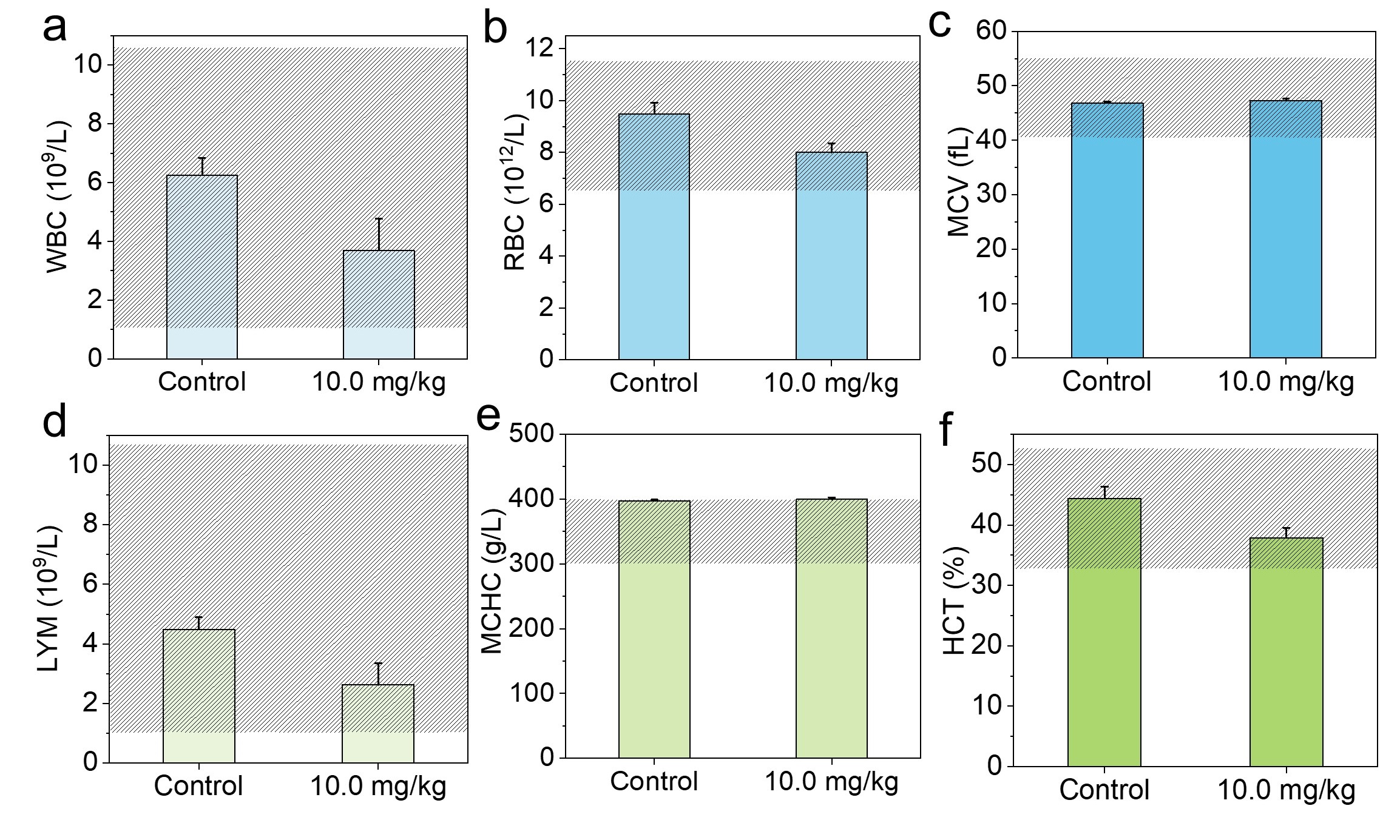


**Figure S36.** Hematological index measurements of mice on the 3^rd^ day after intravenous injection of CuZnONPs with a dosage of 10.0 mg/kg. (a): WBC. (b): RBC. (c): MCV. (d): LYM. (e): MCHC. (f): HCT. Data are presented as mean ± SD (n = 3).


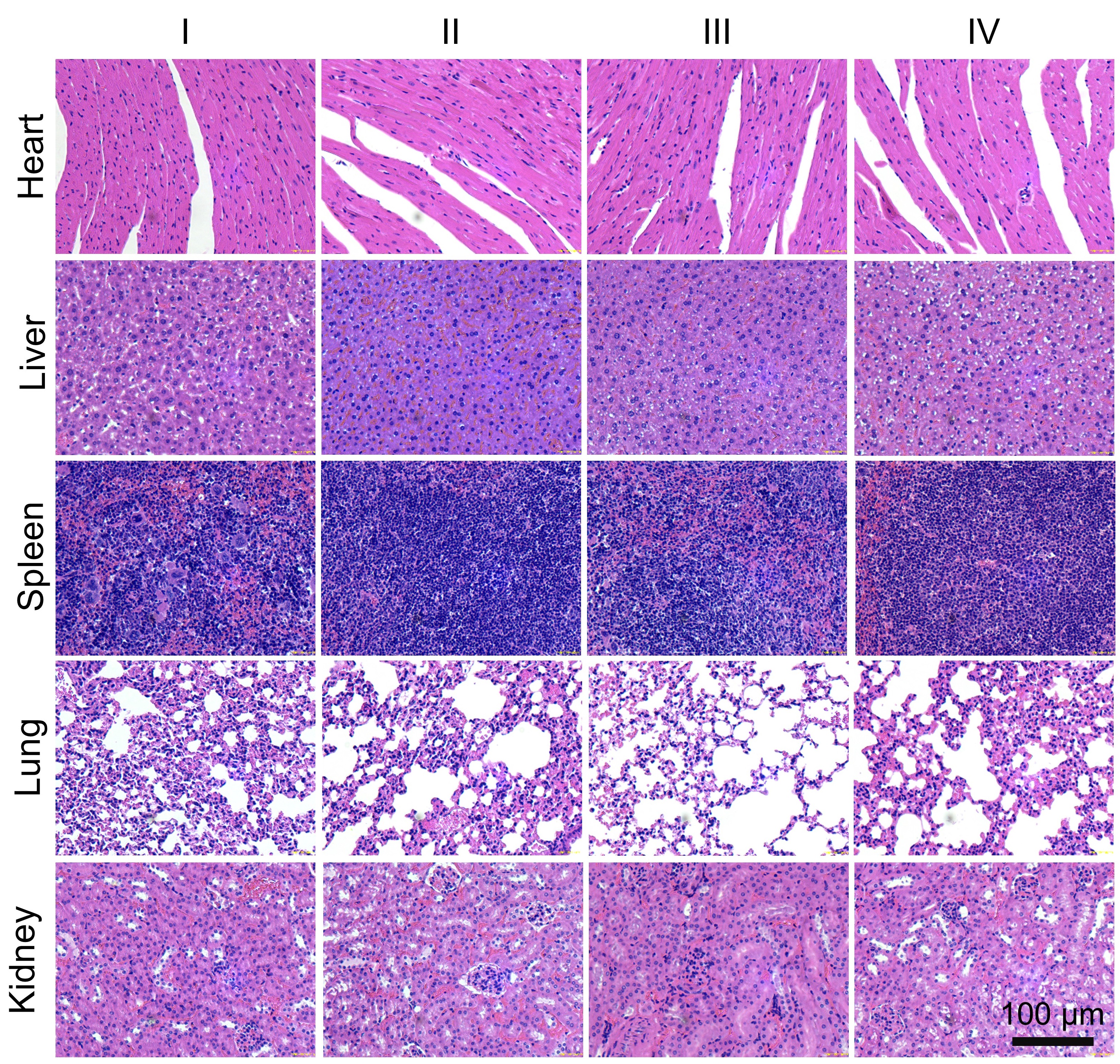


**Figure S37.** H&E-stained images of major organs harvested from mice after treatment with (I) PBS, (II) *α*PD-L1, (III) CuZnONPs, and (IV) *α*PD-L1 + CuZnONPs.
